# Preliminary evaluation of ProSpare^®^, a rectal obturator for prostate bed radiotherapy within the randomised multicentre POPS trial (NCT02978014)

**DOI:** 10.3389/fonc.2026.1758462

**Published:** 2026-10-12

**Authors:** Sarah Stewart, Karen Thomas, Jenni Parmar, Alison Claire Tree, Chris Parker, Helen McNair, Sophie Alexander, Julia Henderson, James Talbot, Christopher Scrase, Chee Goh, Reena Davda, Emma Gray, Peter Hoskin, Sarah Needleman, Jenny Nobes, Kamalram Thippu Jayaprakash, Yvonne Rimmer, Ranachandran Venkitaraman, Anna Wilkins, Vincent Khoo, Julia Murray, David Paul Dearnaley

**Affiliations:** 1 The Institute of Cancer Research, London, United Kingdom; 2 Royal Marsden Hospital, London, United Kingdom; 3 Ipswich Hospital, Ipswich, United Kingdom; 4 Royal Surrey NHS Foundation Trust, Guildford, United Kingdom; 5 University College Hospital, London, United Kingdom; 6 Musgrove Park Hospital, Taunton, United Kingdom; 7 Mount Vernon Cancer Centre, Northwood, United Kingdom; 8 Royal Free London NHS Foundation Trust, London, United Kingdom; 9 Norfolk and Norwich University Hospitals NHS Foundation Trust, Norwich, United Kingdom; 10 Addenbrooke’s Hospital, Cambridge, United Kingdom; 11 Ipswich Hospital NHS Trust, Ipswich, United Kingdom

**Keywords:** image guidance, post-operative, prostate, radiotherapy, toxicity

## Abstract

**Background and purpose:**

The introduction of volumetric modulated arc therapy (VMAT) has enabled dosimetric improvements during prostate bed (PB) radiotherapy; however, the planning target volume (PTV) margins remain substantially greater than for prostate radiotherapy. New approaches are required to further optimise rectal dose and minimise target and organ motion. ProSpare^®^, a self-inserted rectal obturator designed to facilitate daily image guidance, may enhance radiotherapy accuracy and reduce treatment-related morbidity.

**Materials and methods:**

Patients scheduled to receive adjuvant or salvage PB radiotherapy after radical prostatectomy were randomised to either standard-of-care (SOC) PB-IMRT using centre-standard PTV margins and verification protocol (SOC) or PB-IMRT with daily ProSpare^®^-guided radiotherapy using a reduced 5-mm posterior margin. Dose and fractionation schedules were selected by each centre according to protocol recommendations. The primary endpoint is patient-reported EPIC bowel function and bother at 2 years. Secondary endpoints include acute Radiation Therapy Oncology Group (RTOG) and CTCAE toxicity, patient-reported outcomes, device tolerability, and anorectal dosimetry. We report the results from the prespecified analysis of these secondary endpoints.

**Results:**

Between 2017 and 2023, 162 patients were recruited; 158 began radiotherapy and were evaluable at 18 weeks. Acute toxicity data were available for 146 patients (SOC 76/81, ProSpare 70/77). Compared with SOC, ProSpare^®^ was associated with lower 18-week RTOG ≥grade 1 gastrointestinal (GI) and genitourinary (GU) toxicity (39% vs. 31% and 37% vs. 29%, respectively). The incidence of worst CTCAE grade 2 GI and GU toxicity was <6%. Of 51 responders, 50 found ProSpare^®^ tolerable and would recommend its use. Use of ProSpare^®^ resulted in consistently lower ano-rectal dose–volume parameters.

**Conclusion:**

ProSpare^®^-guided prostate bed radiotherapy (PBRT) resulted in lower acute RTOG bowel toxicity at all time points. Patient-reported tolerability was very favourable. Use of ProSpare^®^ improved rectal and anal canal dosimetry.

**Clinical Trial Registration:**

ClinicalTrials.gov, identifier NCT02978014.

## Introduction

VMAT has improved prostate bed radiotherapy dosimetry, but large PTV margins remain, and ProSpare® may further improve treatment accuracy while reducing rectal dose, organ motion, and treatment-related morbidity (1–4). Prostate cancer is the most common male cancer in 112 countries, with 1.4 million men diagnosed annually (5). In the UK, over 9,000 men undergo radical prostatectomy for localised disease each year (6), though international variation persists, with higher rates of surgery relative to external beam radiotherapy (EBRT) in Europe and North America (7). Salvage or adjuvant postoperative radiotherapy to the prostate bed, with or without pelvic lymph node irradiation, is delivered in approximately 20% of cases for recurrent or high-risk disease (8, 9). A meta-analysis found no improvement in event-free survival with adjuvant radiotherapy compared with early salvage radiotherapy (10), with the former being associated with increased bowel and urinary morbidity; as a result, early salvage radiotherapy is generally recommended (9, 11). The Radiation Therapy Oncology Group (RTOG) SPPORT trial also demonstrated benefit from treating pelvic lymph nodes alongside the prostate bed (12).

A persistent challenge in postoperative prostate bed radiotherapy (PBRT) is motion and deformability of the clinical target volume (CTV) and adjacent organs at risk (OARs), contributing to both inter- and intrafraction uncertainty (2, 3). There is no universally accepted consensus on appropriate planning target volume (PTV) margins for PBRT (13). Historically, an isotropic 1-cm margin has been applied (14, 15), but achieving rectal constraints often necessitates compromising PTV coverage.

Approaches used in the prostate-intact setting, such as IMRT and image guidance (16, 17), have shown comparatively limited benefit in PBRT (18). Dose–volume histogram analyses from the RADICALS trial and RTOG consensus constraints demonstrated improved sparing of rectal and bladder tissue with IMRT, yet meeting all OAR dose constraints remains challenging (1, 19). IGRT is desirable in PBRT (20), but fiducial marker insertion into the prostate bed is technically difficult, associated with morbidity, and not routinely adopted (21). Endorectal balloons (ERBs) improve rectal and CTV stability (22, 23) but suffer from reproducibility issues, setup errors, and logistical limitations (24), which have restricted their use. Alternative IGRT solutions are therefore needed to improve accuracy, reduce margins, and spare normal tissues.

ProSpare^®^ (**Figure 1**) is a novel rectal obturator designed for image guidance in PBRT and prostate-intact radiotherapy (4) (Appendix 1; **Figure 1**). The single-use device is self-inserted prior to each treatment fraction. Radio-opaque markers embedded in the anterior and posterior walls delineate the posterior prostate bed CTV border, and venting holes allow passage of rectal gas. Prior work in prostate-intact radiotherapy has shown improved setup accuracy and reduced anorectal dose with ProSpare^®^ (25–27). In PBRT, ProSpare^®^ may permit PTV margin reduction, stabilise prostate bed and anorectal position, and act as a rectal spacer, thereby reducing treatment-related toxicity.

**Figure 1:**
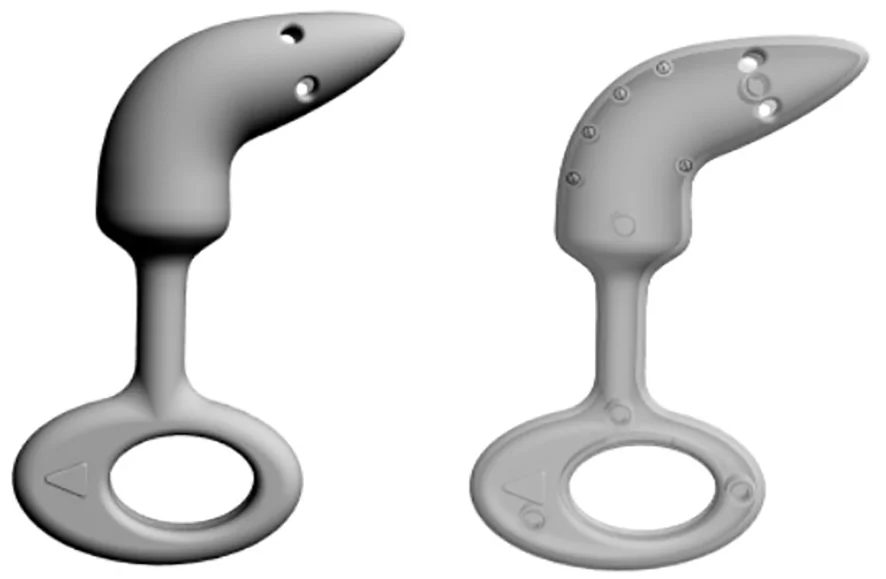
Image of ProSpare^TM^ device.

The POPS randomised controlled trial compares PBRT with and without ProSpare^®^. This preplanned 18-week analysis evaluates clinician-reported acute toxicity, patient-reported outcomes, device tolerability, and anorectal dosimetry.

## Materials and methods

### Study design and patient population

POPS is a randomised, multicentre, open-label phase II clinical trial evaluating the use of a novel rectal obturator, ProSpare^®^, during PBRT (protocol within the Appendix). Eligible participants were men aged ≥18 years, with WHO performance status 0–1 and histologically confirmed prostate cancer, planned to receive adjuvant or salvage PBRT following radical prostatectomy.

Adjuvant radiotherapy (ART) was defined as early postoperative treatment in patients with PSA <0.2 ng/mL who did not meet criteria for biochemical failure. Salvage radiotherapy (SRT) was defined as biochemical recurrence post-prostatectomy, either two consecutive PSA rises with final PSA >0.1 ng/mL, or three consecutive PSA rises.

Participants were randomised 1:1 to either standard-of-care (SOC) PB-IMRT using centre-defined PTV margins and verification protocol, or PB-IMRT with daily ProSpare^®^-guided treatment incorporating a reduced 5-mm posterior PTV margin. Randomisation was managed centrally by the Institute of Cancer Research Clinical Trials and Statistics Unit (ICR-CTSU) using minimisation with a random element. Balancing factors included indication for PBRT (adjuvant vs. salvage), treatment centre, duration of androgen deprivation therapy (ADT), use of a boost to macroscopic disease, and delivery of pelvic lymph node radiotherapy (PLNRT). PLNRT was permitted after a protocol amendment on 21 October 2019.

ProSpare^®^ was approved by the Medicines and Healthcare Products Regulatory Agency (MHRA) for clinical trial use. POPS was conducted in accordance with Good Clinical Practice guidelines. All participants provided written informed consent. The trial is registered on ClinicalTrials.gov (NCT02978014).

### Radiotherapy planning and delivery

Decisions regarding ADT use and duration were made by treating centres prior to randomisation. All patients received to the PBRT using volumetric modulated arc therapy (VMAT) or TomoTherapy delivered daily according to local protocols and all centres had completed radiotherapy quality assurance as in the protocol. Radiotherapy commenced within 8 weeks of randomisation.

Before trial enrolment, ProSpare^®^ tolerability was assessed in clinic, with written consent obtained. Standard preparation followed local policy; patients were instructed to attend with a comfortably full bladder and an empty rectum. Participants randomised to ProSpare^®^ were trained in its correct use and self-inserted the device prior to the planning computed tomography (CT) scan and each radiotherapy fraction. Details on device manufacture, traceability, and training are provided in the protocol.

The CTV was the prostate bed, delineated according to RTOG guidelines (28). For SOC patients, the PTV was defined per centre standards. For the ProSpare^®^ arm, PTV1 was based on previous prostate-intact analyses and defined as CTV + 10-mm isotropic expansion, except posteriorly where a 5-mm margin was used. All centres completed mandatory pre-trial radiotherapy trials quality assurance (RTTQA), including contouring and outlining benchmark cases. The first patient enrolled in each arm at each centre required RTTQA review.

Recommended PBRT doses were 66 Gy in 33 fractions over 6.5 weeks or 55 Gy in 20 fractions over 4 weeks. Boost doses to macroscopic disease and pelvic nodes were permitted. Centres selected dose/fractionation schedules from protocol-approved options. Dose constraints for OARs were mandatory. Additional details regarding target and organ definition, preparation, fractionation, and dose constraints are in the protocol.

### Treatment verification

Patients in the ProSpare^®^ arm underwent daily cone-beam CT (CBCT) after alignment to lasers and skin markers. Automatic reconstruction and registration to the ProSpare^®^ radio-opaque markers were reviewed by treatment radiographers to confirm a satisfactory match. Patients in the SOC arm received centre-standard verification, which was required to meet protocol-defined minimum verification criteria.

### Assessments

Acute toxicity was assessed using RTOG gastrointestinal (GI) and genitourinary (GU) scoring at baseline and weeks 2, 4, 6, 8, 10, and 18. CTCAE v4 GI, GU, and erectile function assessments were performed at baseline and 18 weeks.

Six patient-reported outcome measures (PROMs) were collected using paper questionnaires at baseline and 18 weeks (29):

- EPIC bowel domain
- Vaizey faecal incontinence score
- International Index of Erectile Function (IIEF-5)
- International Prostate Symptom Score (IPSS)
- SF-12 physical and mental health scores
- Modified Inflammatory Bowel Disease Questionnaire (IBDQ)

ProSpare^®^ tolerability was defined as completion of treatment without significant discomfort. Tolerability questionnaires were administered before radiotherapy and at 10 weeks post-treatment.

Dosimetric evaluation of the rectum and anal canal was performed with and without ProSpare^®^, comparing the volume of anorectum receiving 45%–98% of the prescribed dose. The anal canal was defined as extending 3 cm cranially from the anal verge.

### Statistical analysis

The primary endpoint is patient-reported EPIC bowel function and bother at 2 years post-PBRT. The original sample size target of 172 evaluable patients (86 per arm) provided 90% power (two-sided α = 0.05) to detect a mean difference of 5.45 points in EPIC bowel score, based on prior prostate-intact IMRT data (30). Allowing for 30% attrition, the initial target sample size was 245. On 24 August 2022, the target was revised to 160 patients, assuming 20% non-evaluability at 24 months, yielding 80% power with two-sided α = 0.05.

EPIC bowel, IPSS, IIEF-5, IBDQ, Vaizey, and SF-12 scores are summarised as mean (standard deviation) and median (IQR). IPSS QoL score, RTOG GI/GU toxicity, CTCAE GI/GU/erectile toxicity grades, and ProSpare^®^ tolerability are reported as counts and percentages.

Higher EPIC, IIEF-5, IBDQ, and SF-12 scores indicate better function or quality of life; higher IPSS and Vaizey scores indicate greater symptom severity.

This manuscript presents the pre-specified 18-week acute toxicity analysis and are descriptive only. All randomised patients receiving ≥1 fraction of radiotherapy were included. Statistical analyses were conducted using Stata v18.0. Data were extracted on 12 June 2024. The full statistical analysis plan is available in the Appendix.

## Results

Between 8 February 2017 and 22 February 2023, 162 patients were randomised across 10 UK centres to ProSpare^®^-guided IMRT (*n* = 78) or SOC (*n* = 82). Two patients withdrew and had all data removed. In the ProSpare^®^ group, one patient withdrew before radiotherapy due to worsening pre-existing bowel symptoms, and in the SOC group, one patient withdrew for unknown reasons prior to treatment (**Supplementary Appendix Table 1**).

The analysis includes all patients who received at least one radiotherapy fraction (*n* = 158; SOC *n* = 81, ProSpare^®^ *n* = 77). At the time of analysis, median follow-up was 24 months. Eighteen-week assessment data were unavailable for 6/77 (7.7%) patients in the ProSpare^®^ group and 3/81 (3.7%) patients in the SOC group (**Figure 2**).

**Figure 2:**
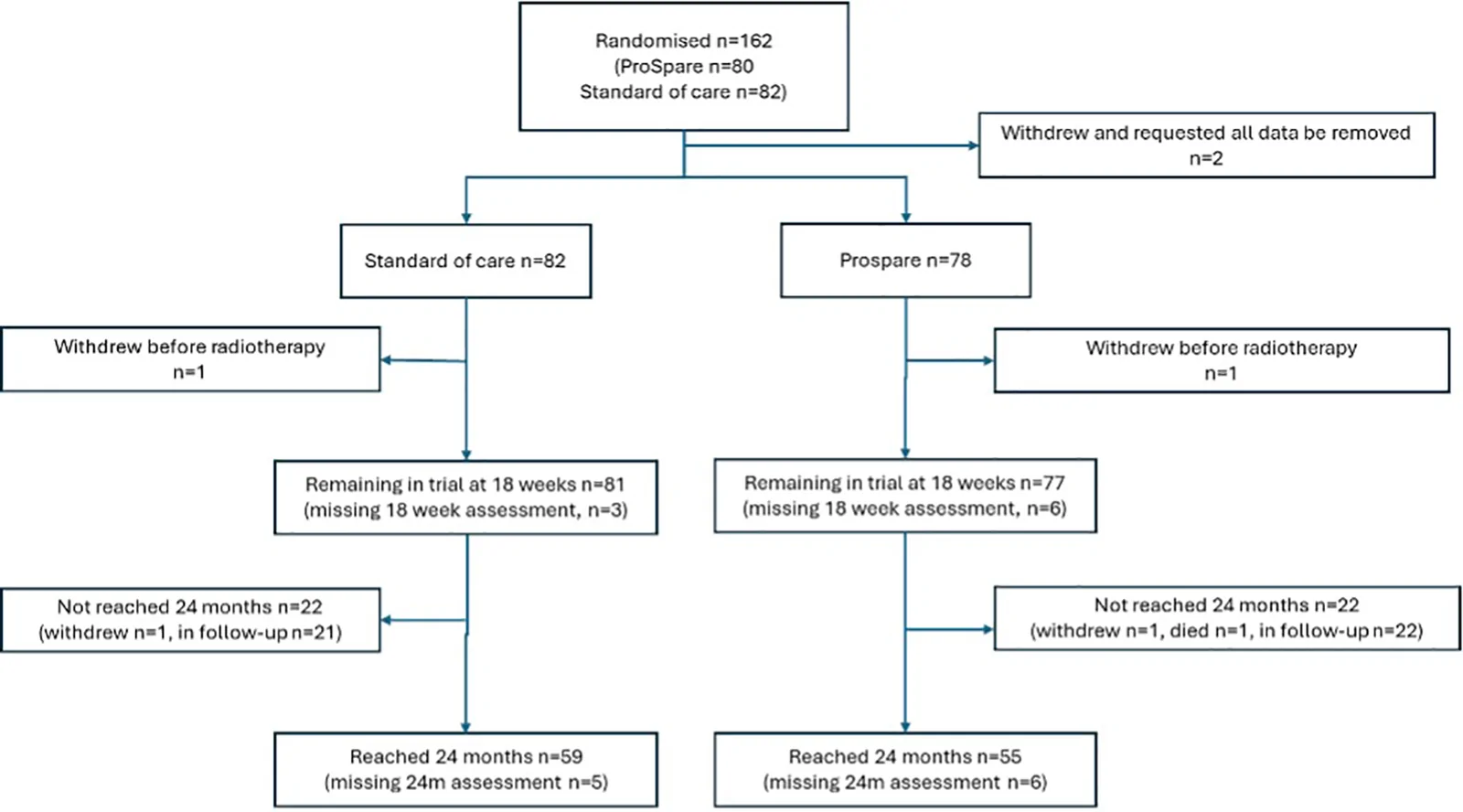
CONSORT diagram. This figure summarises number of patients recruited and randomised to trial, with information on available follow up.

Treatment compliance was high in both arms: 76/77 (98.7%) patients in the ProSpare^®^ group and 79/81 (97.5%) patients in the SOC group received the allocated treatment and planned fractions (**Supplementary Appendix 1, Tables 2**, **3**). The median posterior CTV-to-PTV margin in the SOC group was 10 mm (range: 5–10 mm); these margin data were missing in 3/81 patients. In 33/78 patients, the posterior CTV-to-PTV margin was 7–8 mm, with one patient having a 5-mm posterior CTV-to-PTV margin. Radiotherapy was delivered as planned without modification in 67/77 (87.0%) patients in the ProSpare^®^ arm compared with 65/81 (80.2%) patients in the SOC arm. Dose reductions occurred more frequently in the SOC group (10/81, 12.3%) than in the ProSpare^®^ arm (5/77, 6.5%).

Modest hypofractionation was delivered to 84/158 (53%) patients, and PLNRT was used in 28/158 (17%) patients. Baseline characteristics by treatment arm are summarised in **Table 1**. Stratification factors and concurrent medications were similarly distributed between groups (**Supplementary Appendix Tables 4**, **5**).

**Table 1:** Patient baseline characteristics and treatment.

Characteristics	Randomisation group (patients who received radiotherapy only)					
	Prospare (N=77)		Standard of Care (N=81)		Total	
	N	%	N	%	N	%
Age (years) at radical prostatectomy						
Median (IQR)	63 (59, 68)		65 (59, 69)		64 (59, 68)	
Months from surgery to rand.						
Median (IQR)	21.2 (10.9, 40.3)		26.2 (15.9, 42.8)		23.1 (14, 42.7)	
Gleason grade						
5 or 6	2	2.6	1	1.2	3	1.9
7 (3+4)	37	48.1	38	46.9	75	47.5
7 (4+3)	22	28.6	29	35.8	51	32.3
8 or 9	16	20.8	11	13.6	27	17.1
Not known	0	0.0	2	2.5	2	1.3
WHO Score						
WHO 0	64	83.1	64	79.0	128	81.0
WHO 1	9	11.7	12	14.8	21	13.3
Not available	4	5.2	5	6.2	9	5.7
Positive margins						
Absent	43	55.8	42	51.9	85	53.8
Present	33	42.9	39	48.1	72	45.6
Unknown	1	1.3	0	0.0	1	0.6
Seminal vesicle involvement						
Absent	56	72.7	62	76.5	118	74.7
Present	19	24.7	11	13.6	30	19.0
Not Known	2	2.6	8	9.9	10	6.3
Lymph node involvement						
Absent	53	68.8	59	72.8	112	70.9
Present	10	13.0	3	3.7	13	8.2
Not Known	14	18.2	19	23.5	33	20.9
Lymph node dissection						
Yes	23	29.9	23	28.4	46	29.1
No	47	61.0	50	61.7	97	61.4
Unknown	7	9.1	8	9.9	15	9.5
Previous pelvic surgery*						
Yes	6	7.8	5	6.2	11	7.0
No	66	85.7	64	79.0	130	82.3
Unknown	5	6.5	12	14.8	17	10.8
History of IBD or diverticular disease						
Yes	2	2.6	2	2.5	4	2.5
No	74	96.1	76	93.8	150	94.9
Not available	1	1.3	3	3.7	4	2.5
Symptomatic haemorrhoids in last 12 months:						
Yes	1	1.3	4	4.9	5	3.2
No	75	97.4	74	91.4	149	94.3
Unknown	1	1.3	3	3.7	4	2.5
History of diabetes						
Yes	5	6.5	4	4.9	9	5.7
No	71	92.2	76	93.8	147	93.0
Unknown	1	1.3	1	1.2	2	1.3
Treatment of hypertension						
Yes	23	29.9	24	29.6	47	29.7
No	54	70.1	57	70.4	111	70.3
Anticoagulants taken?						
Yes	11	14.3	8	9.9	19	12.0
No	63	81.8	70	86.4	133	84.2
Unknown	3	3.9	3	3.7	6	3.8
Statin taken?						
Yes	19	24.7	26	32.1	45	28.5
No	56	72.7	52	64.2	108	68.4
Unknown	2	2.6	3	3.7	5	3.2

N = number of patients per group affected. % = percentage of patients affected per group.

WHO, World Health Organisation; IBD, Inflammatory Bowel Disease

*excluding radical prostatectomy +/- pelvic lymphadenectomy

### Acute clinician-reported toxicity

Incidence of RTOG GU and GI toxicity at each assessment point is shown in **Figure 3**. GI grade ≥2 toxicity peaked at week 4 in both groups but remained lower in the ProSpare^®^ arm. Weeks 6 and 8 data were less complete than at other time points.

**Figure 3:**
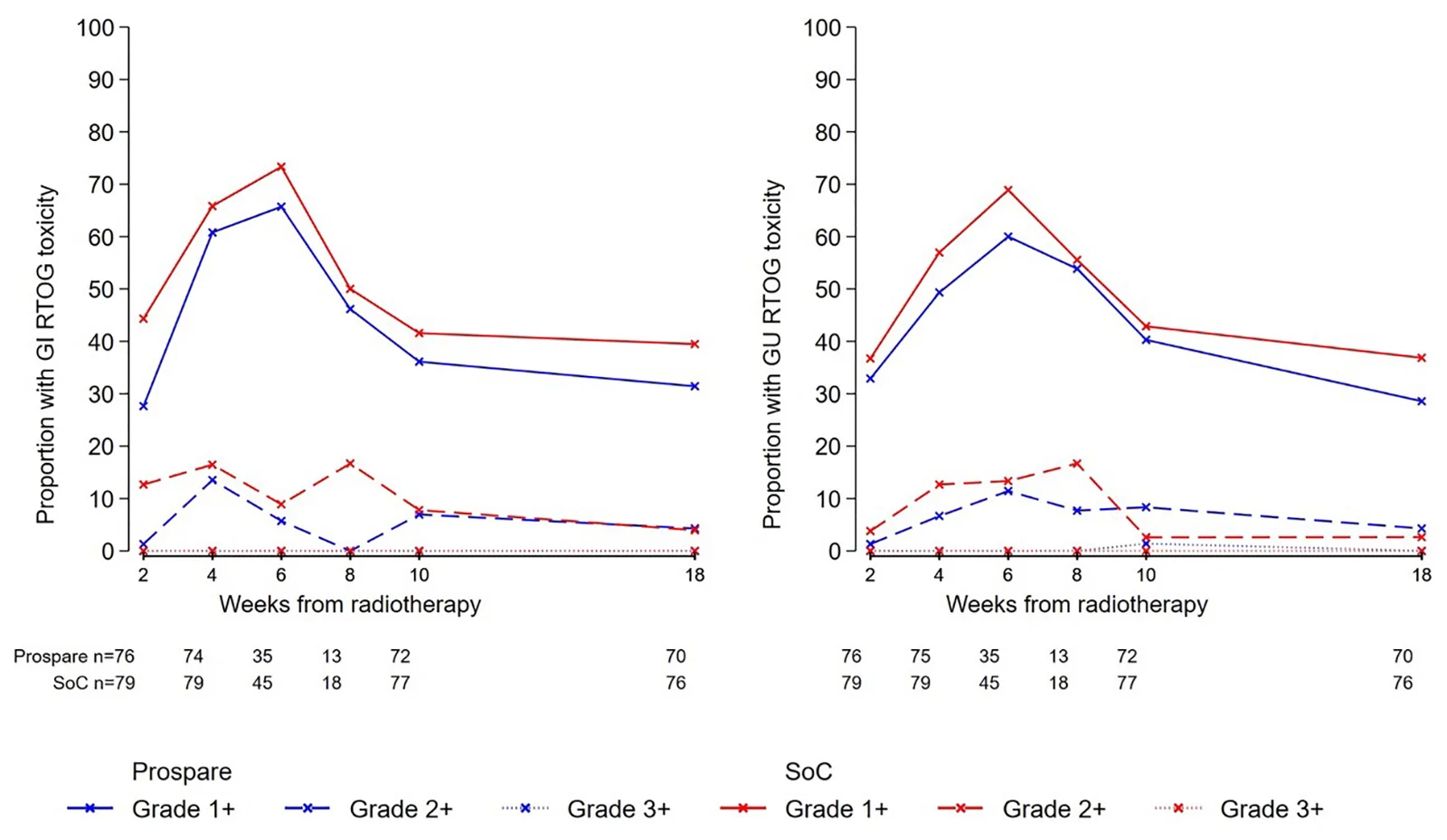
Proportion (percent) of patients with grade 1-3 RTOG GI and GU toxicity.

At 18 weeks (**Table 2**), both GI and GU grade ≥1 toxicity were lower in the ProSpare^®^ group compared with SOC:

**Table 2:** Maximum grade of RTOG GU and GI toxicity at 18 weeks after commencing radiotherapy.

				Randomisation group (patients who received radiotherapy only)						
RTOG	Prospare (N=77, 70 assessed)			Standard of Care (N=81, 76 assessed)			Total			
	N	%	% of assessed	N	%	% of assessed	N	%	% of assessed	
GU score										
Grade 0	50	64.9	71.4	48	59.3	62.3	98	62.0	67.1	
Grade 1	17	22.1	24.2	26	32.1	34.2	43	27.2	29.4	
Grade 2	3	3.9	4.3	2	2.5	2.6	5	3.2	3.4	
Not assessed	7	9.1		5	6.2		12	7.6		
Lower GI score										
Grade 0	48	62.3	68.6	46	56.8	60.5	94	59.5	64.3	
Grade 1	19	24.7	27.1	27	33.3	35.5	46	29.1	31.5	
Grade 2	3	3.9	4.3	3	3.7	4.0	6	3.8	4.1	
Not assessed	7	9.1		5	6.2		12	7.6		

7/77 (9.1%) of patients from ProSpare® arm and 5/81 (6.2%) of patients from SOC arm had missing data.

RTOG, Radiation Therapy Oncology Group; GI, Gastrointestinal; GU, Genito-urinary; SOC, Standard Of Care.

- GI: 22/70 (31.4%) vs. 30/76 (39.4%)
- GU: 20/70 (28.6%) vs. 28/76 (36.8%)

Grade 2 toxicity rates were similar between groups:

- GI: 3/70 (4.3%) ProSpare^®^ vs. 3/76 (4.0%) SOC
- GU: 3/70 (4.3%) ProSpare^®^ vs. 2/76 (2.6%) SOC

No grade ≥3 toxicities were observed at 18 weeks. One grade 3 RTOG bladder toxicity event was recorded at week 10 in the SOC arm.

Worst CTCAE grade 2 GI and GU toxicity at 18 weeks was likewise low. A single CTCAE grade 3 urinary incontinence event occurred in the ProSpare^®^ arm. Erectile dysfunction grade ≥2 was present in 41/56 (73.2%) patients in the ProSpare^®^ group and 46/71 (64.7%) patients in the SOC group. Full CTCAE toxicity summaries for GU, GI, and erectile dysfunction are shown in the **Supplementary Appendix Tables 6**–**8**. There was an imbalance between the groups in baseline rates of erectile dysfunction grade >2. However, the magnitude of change for each arm from baseline to 18 weeks was similar (8.4% in the ProSpare^®^ group versus 8.6% in the SOC group).

### Patient-reported outcomes

**Figure 4** illustrates changes in EPIC bowel, IPSS, Vaizey incontinence, IBDQ, IIEF-5, and SF-12 scores between baseline and 18 weeks post-radiotherapy. Most PROMs demonstrated mild declines from baseline, without meaningful differences between treatment arms. Full mean (SD) and median (IQR) values for each PROM are provided in the **Supplementary Appendix Tables 9**–**16**.

**Figure 4:**
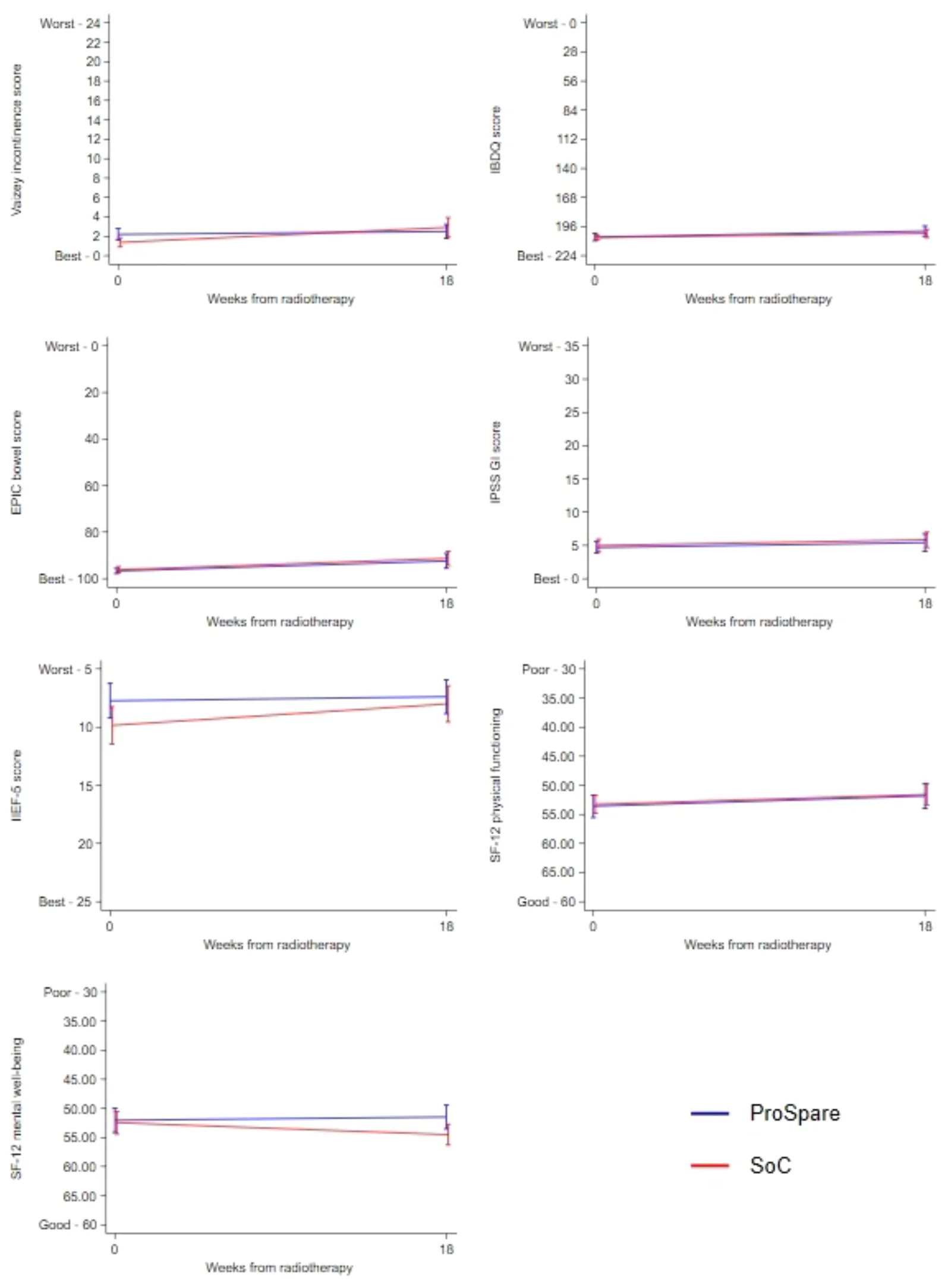
Patient-reported outcome measures at baseline and 18 weeks after commencing radiotherapy. IBDQ, Inflammatory Bowel Disease Questionnaire; EPIC, Expanded Prostate Cancer Index Composite; I-PSS, International Prostate Symptom Score; IIEF-5, International Index of Erectile Function; SF-12, 12 item Short Form health survey.

Baseline IIEF-5 categorical scores were worse in the ProSpare^®^ arm, with 80% of responding patients reporting severe erectile symptoms, compared with 57% in the SOC group. At 18 weeks, this remained unchanged in the ProSpare^®^ group (79%), whereas the SOC group had increased to 74.5%.

### Patient tolerability of ProSpare^®^

All patients randomised to receive radiotherapy with ProSpare^®^ completed all their radiotherapy treatments using ProSpare^®^. Among respondents, 98% (50/51) found ProSpare^®^ tolerable during treatment. Most reported that they would recommend the device to reduce radiotherapy side effects (49/51, 96.1%) or improve accuracy (50/51, 98%). After initial familiarity, 82.8% found ProSpare^®^ very or quite easy to insert, and 98.5% reported no or only minor discomfort. Additional details and free-text comments are provided in Appendix 1 (**Tables 17**, **18**) and Appendix 2.

No serious adverse events or device deficiencies were reported.

### Dosimetric comparison

Dosimetric analyses included 122 patients (*n* = 61 per arm). The volume of anal canal and rectum receiving specified percentages (45%–98%) of the prescribed dose was reduced across all measured dose levels in the ProSpare^®^ arm (**Supplementary Table 18**). This is shown graphically in **Figure 5** and detailed in the **Supplementary Appendix Tables 19**, **20**.

**Figure 5:**
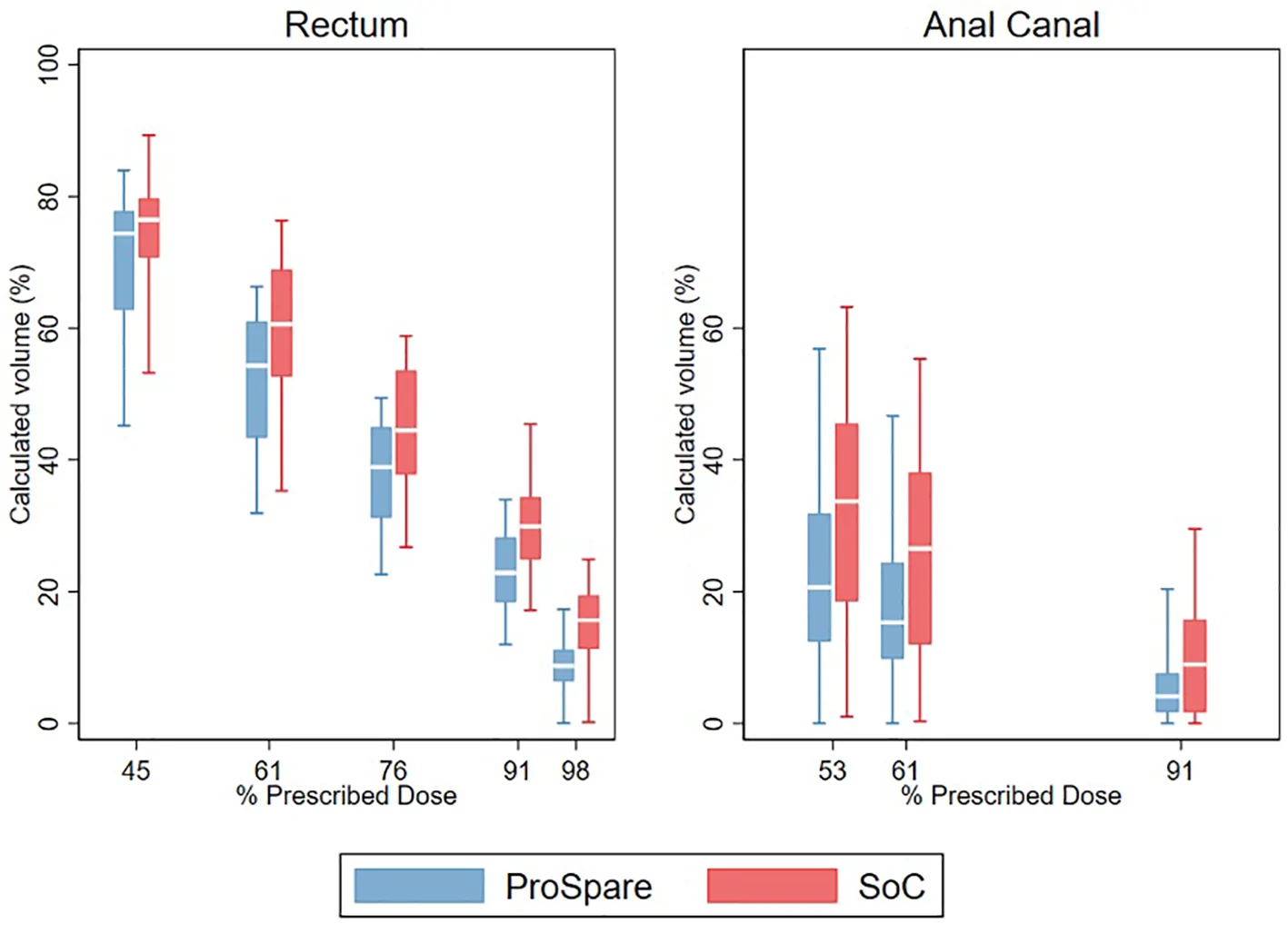
Ano-rectal dosimetry with and without ProSpare® use.

Median dose reductions ranged from 2.7% to 44.6% for rectum and 39.8% to 54.5% for anal canal. Reductions were greatest at higher dose levels.

## Discussion

ProSpare^®^-guided IGRT for PBRT was well tolerated and associated with modest reductions in acute GI and GU toxicity. Improvements in anorectal dosimetry were accompanied by fewer treatment modifications and dose reductions (6%–7% absolute reduction), although the details regarding the reason for the dose modifications were not collected. Our previous single-centre studies in patients with an intact prostate and implanted fiducial markers showed that ProSpare^®^ preserved much of the positional accuracy achieved with fiducials, particularly in the anteroposterior direction. This therefore allowed the reduction of the posterior PTV margin to 5 mm to be adopted in POPS. ProSpare^®^ also stabilised prostate and rectal position, reducing both inter- and intrafraction motion (3, 26). Ano-rectal dose reductions were observed, and modelling studies predicted lower rates of rectal bleeding and anal incontinence (25, 27).

IGRT is desirable for PBRT given the susceptibility of the CTV to positional changes driven by variations in rectal and bladder filling (20, 31, 32). Although IGRT approaches such as fiducial markers (20) or radiopaque hydrogel spacers (33) have potential applications, they carry additional risks in the post-prostatectomy setting and currently no randomised evidence. ERBs may improve organ stability but have limitations including setup variability and inconsistent dosimetric benefit (22–24).

The emergence of MR-guided radiotherapy (MRgRT) offers another potential solution to the challenges of soft-tissue visualisation and motion management in PBRT. MRgRT provides superior soft-tissue contrast compared with CBCT, enabling improved daily localisation of the prostate bed as well as real-time visualisation of intrafraction motion. Early studies of MR-guided stereotactic PBRT have reported promising patient and clinician-reported outcomes (34). Online adaptive workflows allow adjustments for daily anatomical changes, which may be particularly beneficial in PBRT where CTV position can vary considerably. However, the clinical adoption of MRgRT is currently constrained by longer treatment times, resource requirements, and limited MR-linac availability. In this context, ProSpare^®^ may provide a pragmatic alternative for centres without MR-guided technology, delivering improved rectal stabilisation and reproducible delineation of the anterior rectal wall. Comparative evaluation of MRgRT and ProSpare^®^-guided workflows would help clarify the relative benefits and whether a combined approach could further improve precision and reduce adaptation burden.

VMAT is also increasingly used for PBRT due to its advantages in treatment efficiency and dose conformity. VMAT can achieve highly conformal dose distributions with reduced treatment times and potentially improved OAR sparing compared with static-field IMRT. Planning studies have reported comparable or superior target coverage and rectal dose metrics with VMAT in the postoperative setting (35). Nonetheless, VMAT remains similarly affected by prostate bed and rectal motion, and the dosimetric benefits of VMAT alone may be limited when anatomical variability is significant. ProSpare^®^ may complement VMAT delivery by improving prostate bed stability and reducing rectal volume within high-dose regions, thereby enhancing the robustness of VMAT plans. Future studies assessing VMAT in combination with ProSpare^®^, including motion-tracking and dosimetric endpoints, could clarify whether this pairing enables margin reduction or supports broader adoption of hypofractionated PBRT.

Emerging data support stereotactic, hypofractionated PBRT delivered on magnetic resonance imaging (MRI)-guided linacs (34). ProSpare^®^ may be especially advantageous in this context: its line of radio-opaque ball bearings defines the anterior rectal wall, facilitating accurate daily corrections for organ motion and rectal changes. This may allow precise hypofractionated PBRT delivery without requiring MRI-based platforms. Enhanced dosimetric consistency to the CTV may improve tumour control whilst reducing dose variability to the rectum and bladder.

Strengths of POPS include its multicentre design, contemporary treatment delivery with IMRT, and the predominance of salvage PBRT (>95%), consistent with modern international practice (5). Pre-trial QA activities improved consistency in CTV and PTV delineation. Dose constraints were mandatory, and the use of PROMs alongside clinician-reported outcomes strengthened detection of treatment-related effects. As PROMs may better reflect patient experience than clinician assessments (36, 37), the primary endpoint of EPIC bowel function is appropriately patient-centred. POPS represents one of the few randomised studies to evaluate post-prostatectomy IMRT quality-of-life outcomes in detail. The change in bowel domain scores observed in POPS at 18 weeks (4.3–4.9) aligns with those reported in NRG-GU003 at the end of RT (7.28–15.65) and at 6 months (3.07–3.21) (38). The NRG-GU003 showed that for PBRT, a hypofractionated schedule (62.5 Gy in 25 fractions) was not clinically inferior to conventional fractionation (66.6 Gy in 37 fractions) for patient-reported GU or GI symptoms at 2 years.

Limitations include the modest sample size and slow recruitment. The COVID-19 pandemic affected trial activations, reduced radical prostatectomy rates, and limited patient participation (6). Baseline imbalance in patient clinical characteristics such as seminal vesicle involvement (24.7% in ProSpare^®^ arm versus 13.6% in SOC arm) may have some impact on the size of the planned CTV. Centre-defined protocol for SOC arm including a difference in posterior CTV-to-PTV margin between centres may have created a volumetric and dosimetric variation within the SOC arm. However, this consequence would most likely blunt the differences in the ano-rectal dosimetry between the SOC and ProSpare^®^ arms. Evolving data during the trial recruitment period including the RTOG SPPORT trial have highlighted the benefit of pelvic nodal radiotherapy with PBRT (12), albeit with increased acute GI toxicity. PLNRT use may increase, and the POPS protocol was amended to accommodate this; 17% of participants received PLNRT. Modest hypofractionation (32) was delivered to 53% of patients, which may also increase acute GI toxicity (38). Subset analyses of PLNRT and hypofractionation are planned but will be hypothesis-generating due to limited numbers.

In conclusion, ProSpare^®^-guided PBRT is well tolerated and improves anorectal dosimetry without adversely affecting acute toxicity or patient-reported outcomes. Ongoing follow-up will determine whether these acute and dosimetric benefits translate into reductions in patient- and clinician-reported outcomes.

## Data Availability

The original contributions presented in the study are included in the article/**Supplementary Material**. Further inquiries can be directed to the corresponding author.
