# Supplementary material for: Preliminary evaluation of ProSpare®, a rectal obturator for prostate bed radiotherapy within the randomised multicentre POPS trial (NCT02978014)
Source: Front Oncol. 2026 Oct 12;16:1758462. doi: 10.3389/fonc.2026.1758462 (PMC13696223; doi:10.3389/fonc.2026.1758462)
Supplement: Supplementary file 1 [file DataSheet1.pdf]

Protocol Reference:	CCR4359
Version Number & Date:	Version: 2.0 Date: 16/03/2016
Effective Date:	
Review Date:	
Superseded Version Number & Date (If applicable)	1.1 13/08/2015

Revision Chronology:	Effective Date	Reason for Change
Version Number		
2.0	TBA	Updated document footer<br>Inclusion of signature pages (Section 17)<br>Text change (Section 7, ProSpare)<br>Text clarification (Section 7, Radiation Treatment)<br>Text clarification (Section 7, Planning Target Volume)<br>Update to table (Section 7, Planning Target Volume)<br>Update to Contents List

Title: A randomised phase II trial assessing **P**ost-**O**perative use of **P**ro**S**pare™, a rectal obturator in prostate cancer radiotherapy

Short Title: POPS

Chief Investigator: Professor David Dearnaley
Professor of Uro-Oncology, Institute of Cancer Research and Royal Marsden NHS Foundation Trust

Statistician: Karen Thomas

Clinical Trials Unit: Sally Ellis and Linda Wedlake, Royal Marsden Hospital Clinical Trials Unit

Sponsor: The Institute of Cancer Research, 15 Cotswold Road, Sutton, Surrey, SM2 5NG

Protocol writing group: Dr Julia Murray
Dr Emma Hall
Prof David Dearnaley
Karen Thomas

Protocol was developed at the Methods in Clinical Cancer Research Workshop, Flims (June 2014)

Potential Investigator sites:

Barts and the London NHS Trust, The Royal London Hospital, Whitechapel, London, E1 1BB

Brighton and Sussex University Hospitals, Royal Sussex County Hospital, Eastern Road, Brighton, BN2 5BE

Cambridge University Hospitals NHS Foundation Trust, Addenbrookes Hospital, Hills Road, Cambridge, CB2 0QQ

Clatterbridge Centre for Oncology, Clatterbridge Road, Bebington, CH63 4JY

East Kent Hospitals University, Kent & Canterbury Hospital, Ethelbert Road, Canterbury, CT1 3NG

Guy's and St Thomas' NHS Foundation Trust, Guy's Hospital, Great Maze Pond, London, SE1 9RT

Ipswich Hospital NHS Trust, Heath Road, Ipswich, IP4 5PD

Leeds Teaching Hospitals NHS Foundation Trust, St. James' University Hospital Beckett Street, Leeds, LS9 7TF

Royal Devon and Exeter NHS Foundation Trust, Royal Devon & Exeter Hospital, Barrack Road, Exeter, Devon, EX2 5DW

Royal Free Hampstead NHS Trust, Royal Free Hospital, Pond Street, London, NW3 2QG

The Christie NHS Foundation Trust, 550 Wilmslow Road, Manchester, M20 4BX

The Newcastle Upon Tyne Hospitals NHS Foundation Trust, Freeman Hospital, Freeman Road,

The Royal Marsden NHS Foundation Trust, Downs Road, Sutton, Surrey, SM2 5PT

The Royal Surrey County Hospital, St Luke's Cancer Centre, Egerton Road, Guildford, GU2 7XX

University College London Hospitals NHS Foundation Trust, 250 Euston Road, London, NW1 2PG

University Hospital Birmingham, Queen Elizabeth Medical Centre, Edgbaston, Birmingham, B15 2TH

University Hospitals Coventry, Walsgrave General Hospital, Clifford Bridge Road, Coventry, CV2 2DX

United Lincolnshire Hospitals, Lincoln County Hospital, Greetwell Road, Lincoln, LN2 4AX

Velindre Cancer Centre, Velindre Road, Cardiff, CF14 2TL

Table of Contents

1 Protocol synopsis 5
2 List of abbreviations: 7
3 Background 8
Prostate bed radiotherapy 8
ProSpare™ 10
Rationale 11
4 Study Objectives 12
5 Study Design 13
Stratification 13
Randomisation 13
Study Flow Chart 14
6 Study Population 15
7 Study Treatment and Procedures 16
ProSpare™ 16
Hormonal treatment 16
Radiation treatment 16
Patient's positioning and preparation 17
Simulation (patient data acquisition) guidelines 17
Definition of treatment volumes 17
Organs at Risk (OAR) 19
External Beam Equipment and Technique 21
Dose prescription, recording and reporting 21
Dose prescription *21*
Dose recording *23*
Dose volume constraints to organs at risk *23*
Radiotherapy Delays 24
Treatment Verification 24
Clinical evaluation, laboratory tests, follow-up 25
Pre-randomisation investigations 25
Pre-Treatment 25
Assessment During Radiotherapy Treatment 26
Immediate Post-Radiotherapy Safety Follow-Up 26
Week 10 from start of radiotherapy: *26*
Week 18 from start of radiotherapy: *26*
Post-Radiotherapy Treatment Follow-Up 26
Assessment of Disease Recurrence 27
Summary table of clinical evaluation assessments 27
8 Criteria of Evaluation 29
Primary Endpoint 29
Secondary Endpoints 29
Exploratory Endpoints 32
9 Study Organisation, Trial Monitoring and Management Strategy 32
Regulatory issues 33
Patient Confidentiality and Data Sharing 34
Data Acquisition 37
Central Data Monitoring 37
Study Management Structure 37

Independent data monitoring committee (IDMC) 42

10 Safety Reporting 43

10.1 Definitions 43

10.2 Operational definitions for (S)AEs 45

10.3 Reporting of Adverse Events 47

11 Statistical Considerations 49

Sample size 49

Randomization and stratification 50

Statistical Analysis 51

Data monitoring 53

12 Quality of life assessment 54

Rationale 54

QoL instruments 55

Statistical considerations 59

13 Radiotherapy Quality Assurance (QA) 59

Nine step IMRT credentialing program 59

Pre-trial quality assurance programme 60

14 Ethical considerations 60

15 Administrative responsibilities 61

Responsibilities of participating centres 61

Trial Insurance 62

16 References: 63

17 Signature pages: 65

1 Protocol synopsis

Title

A randomised phase II trial assessing **P**ost-**O**perative use of **P**ro**S**pare™, a rectal obturator in prostate cancer radiotherapy

Study Design

A multicentre phase II randomised trial. Patients requiring prostate bed radiotherapy will be randomised to either receive IMRT to the prostate bed with centre standard treatment verification protocol or IMRT to the prostate bed with daily ProSpare™ guided radiotherapy.

Study Objectives:

- Primary
    - To compare the EPIC bowel scores for patients receiving adjuvant or salvage prostate bed radiotherapy treated with ProSpare™ image–guided IMRT versus standard arm of IMRT at 2 years following radiotherapy.

- Secondary
    - To compare acute and late bowel and bladder toxicity and sexual function between the two study arms
    - To compare quality of life in patients randomized to the two trial arms
    - To evaluate biochemical progression free survival
    - To analyse the dosimetric consequences of the two radiotherapy treatment techniques and relate these to toxicity endpoints

Inclusion criteria:

⇒ Patient has undergone radical prostatectomy

⇒ Histologically confirmed prostatic cancer

⇒ Patient due to receive post-operative prostate bed radiotherapy either in the adjuvant or salvage* setting

* Adjuvant radiotherapy defined as planned early post-operative treatment in patients with PSA $\leq$0.2 ng/ml and not meeting the definition of PSA failure for salvage therapy. Salvage radiotherapy defined as post-operative biochemical failure defined as either two consecutive rises in PSA and final PSA >0.1ng/ml or three consecutive rises in PSA.

⇒ WHO performance status 0-1 at randomization

⇒ Normal anal tone assessed on rectal examination

⇒ Age $\geq$ 18 years

⇒ Written informed consent

⇒ Able to independently complete patient questionnaires

Exclusion criteria:

⇒ Lymph node or distant metastases from prostate cancer

⇒ Pelvic lymph node radiotherapy planned

⇒ Prior pelvic radiotherapy

⇒ Previous invasive cancer in the past 3 years, with the exception of non-melanoma skin cancer

⇒ Symptomatic haemorrhoids or other anal conditions which affect adequate insertion and retention of the rectal obturator

⇒ Planned chemotherapy within 2 months of completion of adjuvant or salvage radiotherapy

⇒ Musculoskeletal conditions which limit flexibility or mobility and will make patient self insertion of ProSpare™ difficult

⇒ Comorbid conditions likely to impact on advisability of prostate bed radiotherapy (e.g. inflammatory bowel disease, previous colorectal surgery, significant bladder instability or urinary incontinence)

Number of patients: 245 patients

Trial treatment

All patients within the trial will be treated with post-prostatectomy intensity modulated radiotherapy. Depending on randomization, patients will either receive their centre's standard image guidance technique or daily online-ProSpare™ image guidance with a reduced posterior clinical target volume to planning target volume margin.

Endpoints

Primary Endpoints:

- Patient reported EPIC bowel function and bother 2 years after prostate bed radiotherapy.

Secondary Endpoints:

- Acute bowel and bladder toxicity at week 18 from start of radiotherapy (CTCAEv4, RTOG, Gulliford Rectal scoring, EPIC, IPSS)
- Late (1 and 2 year) bowel and bladder toxicity (EPIC, RTOG, modified IBDQ, IPSS)
- Sexual bother and function and change in patient reported erectile function (EPIC, IIEF-5)
- Faecal incontinence (Vaizey incontinence questionnaire)
- Biochemical progression free survival
- Dosimetric assessment of prostate bed and rectal dose with and without ProSpare

Follow-up

Patients will undergo follow-up as per routine clinical practice
Acute toxicity will be assessed during radiotherapy (RT), at week 10 and at week 18 from the start of RT
Late toxicity will be assessed at 6, 12, 18, 24 months from start of RT
Patient reported outcomes will be assessed at baseline, week 10, week 18, and then at 6, 12, 18, 24, 36, 48 and 60 months from the start of radiotherapy
Routine clinical follow-up after 2 years will be centre dependent

2 List of abbreviations:

AE	Adverse Event
A-P	Anterior-posterior
bPFS	Biochemical progression free survival
CHHiP	Conventional or Hypofractionated High Dose Intensity Modulated Radiotherapy for Prostate Cancer
CTV	Clinical Target Volume
EPIC	Expanded Prostate Cancer Index Composite
ERBs	Endorectal balloons™
IBDQ	Inflammatory Bowel Disease Questionnaire
ICRU	International Commission on Radiation Units and Measurements
IDMC	Independent Data Monitoring Committee
IG-IMRT	Image-Guided, Intensity Modulated Radiotherapy
IGRT	Image-Guided Radiotherapy
IMRT	Intensity Modulated Radiotherapy
IPSS	International Prostate Symptom Score
MRI	Magnetic Resonance Imaging
PARSPORT	Parotid Sparing IMRT Trial
PSA	Prostate Specific Antigen
PTV	Planning Target Volume

3 Background

Prostate cancer is the most common male cancer in the United Kingdom with around 41000 men in the UK diagnosed with prostate cancer and just over 10,000 deaths per annum[1]. In England, there are approximately 5000 radical prostatectomies carried out annually. This is in contrast to France where around 25000 such operations are performed. It is therefore likely that the numbers of men undergoing radical prostatectomy will increase in the future. A very conservative estimate by the British Association of Urological surgeons is that by 2014/2015 the number of operations per year will be 8000-10000[2].

Prostate bed radiotherapy

Recurrence risk after radical prostatectomy is greater among men with adverse pathology such as positive surgical margins, seminal vesicle invasion, extraprostatic extension and higher Gleason scores. Radiotherapy delivered post-prostatectomy to patients at higher risk of recurrence because of adverse pathological features prior to evidence of disease recurrence (ie. undetectable PSA) is known as adjuvant radiotherapy. Whereas, salvage radiotherapy is given to a patient with PSA recurrence (>0.2ng/ml) after surgery, but no evidence of metastatic disease[3].

Three randomised trials have addressed the significance of adjuvant radiotherapy, demonstrating a nearly 20% absolute benefit for biochemical progression-free survival at 5 years after adjuvant radiotherapy compared with a "wait and see" policy for patients with pT3± involved surgical margins [4-6]. In all three trials, adjuvant radiotherapy increased the risk of normal tissue complications including bowel and bladder function.

Intensity modulated radiotherapy (IMRT) allows for better sparing of critical normal structures in radical prostate radiotherapy. Studies comparing IMRT and conformal planning for definitive prostate radiotherapy have shown that the reduction in dose to the bladder and rectum by use of IMRT has reduced the incidence of gastrointestinal and genitourinary toxicity[7]. However, in prostate bed radiotherapy, IMRT does reduce the volume receiving a relatively high percentage of prescription dose, yet tissue complication modeling did not show a significant benefit [8].

There is no clear consensus regarding CTV delineation for postoperative prostate radiotherapy. DVH analysis was performed using four published guidelines for CTV delineation with the constraints proposed in Quantitative Analysis of Normal Tissue Effects in the Clinic (QUANTEC) and RADICALS trial. The RADICALS trial is a large multinational trial investigating the optimal timing of adjuvant versus early salvage radiotherapy and the role of hormonal therapy in patients with high-risk disease. Comparison between 3D

conformal radiotherapy and tomotherapy IMRT showed that the later reduced organs at risk irradiation, however, a significant percentage of cases despite using IMRT did not meet the organ at risk dose constraints [9]. Therefore, other methods to spare organs at risk are needed.
A predominant source of intrafraction error is prostate bed motion and there is an interest in determining the best treatment margin to account for daily setup uncertainty and prostate bed motion. There is a lack of data regarding the best treatment margin to account for daily setup uncertainty and organ motion. Surgical clips have been used as fiducial markers for the prostate bed, with their positional shifts captured by kV images and recorded [10]. Implantation of gold seeds and electromagnetic transponders into the prostate bed has been described and data regarding prostate bed motion published [11], however, implantation of these markers is associated with morbidity [12].

Currently in the UK, most centres use an *offline* imaging protocol assessing bony anatomy displacement as a surrogate for prostate bed displacement. This usually entails orthogonal megavoltage images but some centres may use in room CT devices, such as cone-beam CT (CBCT). Images are taken daily in the first few days or for the first week then the displacements are calculated in each direction and averaged over these days. Appropriate corrections are made retrospectively and then applied, imaging is then continued weekly as a check that the treatment position is stable.
Rectal motion during conformal radiotherapy after radical prostatectomy has shown a systematic anterior shift of the cranial half of the rectal wall with a consequent inclusion of larger portions of the rectum in the treatment field than the one expected from the planning scan[13]. Prostate bed motion represents a comparatively smaller component of the total error in the left-right and superior-inferior direction compared with bony misalignment, being the dominant factor contributing to the shifts. However, in the anterior-posterior motion, the prostate bed motion was significant[14].
In prostate bed radiotherapy the target volume is susceptible to anatomical variations with its borders interfacing with the rectum and bladder. Variation in location of the prostate bed is significantly influenced by the changing shape and volume of the rectum and bladder between and during treatments.
Uncertainties due to patient set-up errors and prostate bed motion require a margin around the CTV and this is in the order of around 1cm in all directions[15]. During radiotherapy after radical prostatectomy, the rectum has been shown to migrate anteriorly consistent with post-surgery settlement effects and/or some modification of rectum mobility due to normal tissue effects of radiation. Published data using image-guided prostate radiotherapy identified at localization, prostate bed displacement relative to bony anatomy exceeded 5mm in 9% of fractions in the anterior-posterior (A-P) direction and 21% of fractions in the superior-inferior direction. In the A-P direction, target motion was more likely to move posteriorly, toward the rectum than anteriorly. Fifteen percent of all treatments were interrupted for repositioning, and 70% of patients were repositioned at least once during their treatment course [14].

ProSpare™

ProSpare™ is a daily image-guidance tool and is a single use self-insertable rectal obturator made from high impact polystyrene, which is inserted by the patient just prior to radiotherapy. It has radio-opaque markers (ball-bearings) encased in the anterior and posterior wall of the device, which allows clear identification of the posterior border of the prostate bed CTV. It has venting holes in the tip of the device and a venting line along the central join of the device to allow the passage of rectal gas through the device and to deflate any rectal air bubbles present on insertion.

ProSpare™ has been developed and evaluated at the Institute of Cancer Research and Royal Marsden Hospital in partnership with Sussex Development Services LLP, who are the named manufacturer of the device. The ProSpare™ device has undergone modifications as part of the development process and the current model, which will be used in the trial is known as ProSpare II. ProSpare II devices will be manufactured by Europlaz Technologies Limited who is a medical device sub-contract manufacturer.

Rectal stabilisers, such as ProSpare™ and endorectal balloons can help reduce this variability and thereby increase treatment accuracy thus allowing reduction in treatment margins[16]. There are several studies, which have shown a significant improvement in prostate positional variability with the use of endorectal balloons during radiotherapy, especially in the important AP direction. However, data also suggests that with ERBs positioning and inflation errors can occur, introducing systematic ± random errors into daily treatments. Tolerability is generally felt to be acceptable but there are different designs and products on the market with variable levels of acceptability to patients and clinical staff. In terms of rectal sparing, favourable rectal dosimetry has been demonstrated by several groups when using 3D conformal techniques, but has yet to be shown significant with the use of IMRT [17]. ERBs have not become widely accepted and they are generally unpopular with clinical staff due to the additional time for staff and patients at the treatment machine for insertion.

ProSpare™ has been developed as a minimally invasive, non-surgical alternative method of verifying the location of the prostate or prostate bed. Other design features and theoretical advantages of this rectal obturator include better sparing of the rectal tissue, by acting as a physical endorectal spacer device. In addition it may also increase the stability of the prostate in terms of inter and intra-fraction motion by reducing variation in rectal diameter and volume changes during treatment. ProSpare™ has excellent tolerability in radical prostate radiotherapy patients and there is a pilot study in post-prostatectomy patients assessing tolerability in this new group of patients. The pilot study is also looking at localization accuracy of ProSpare™ in relation to the prostate bed and along with the results from the prostate intact patients will inform us of the necessary PTV margins of using ProSpare™ online image guidance instead of

standard off-line bony anatomy. In the initial part of the pilot study (n=11), calculation of PTV margins using van Herk formula suggested a 50% reduction in margin size in the AP direction with the use of online ProSpare™ compared with offline bony anatomy. In the final part of the pilot study (n=19), calculation of PTV margins using van Herk formula suggested a 4.8mm margin in the AP direction with the use of online ProSpare™.

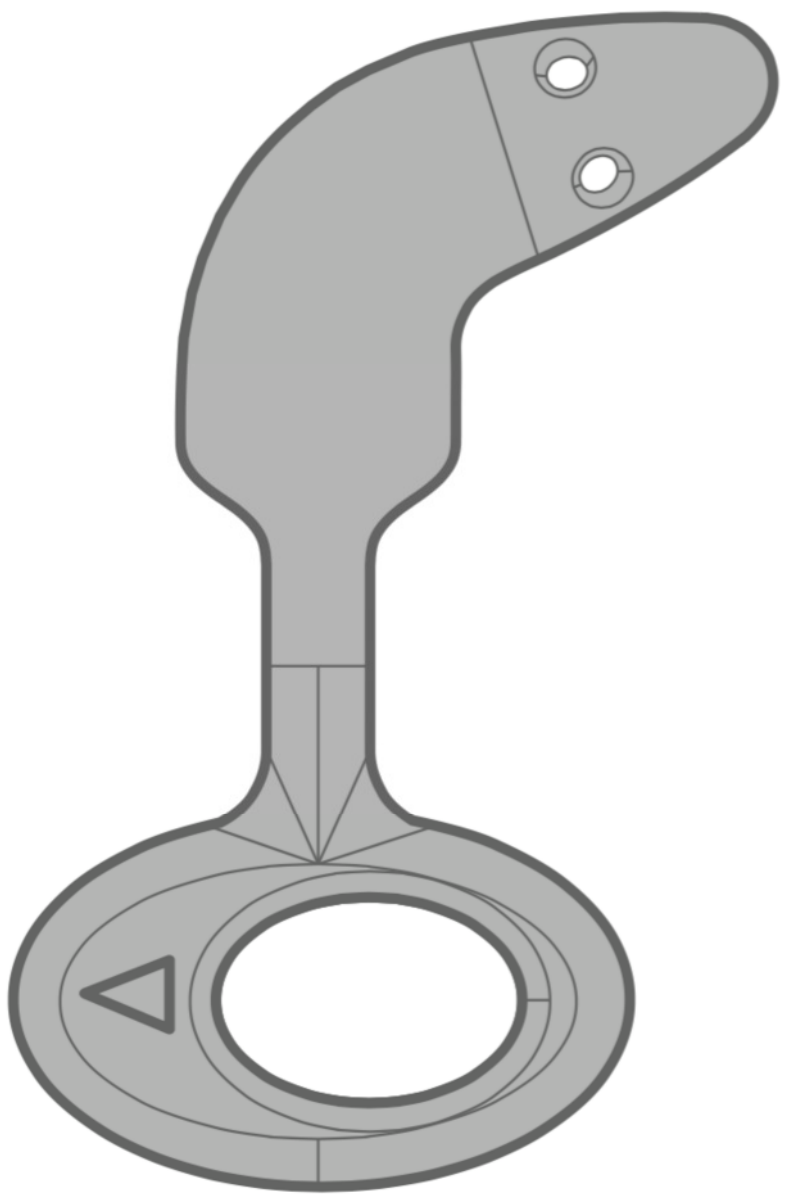

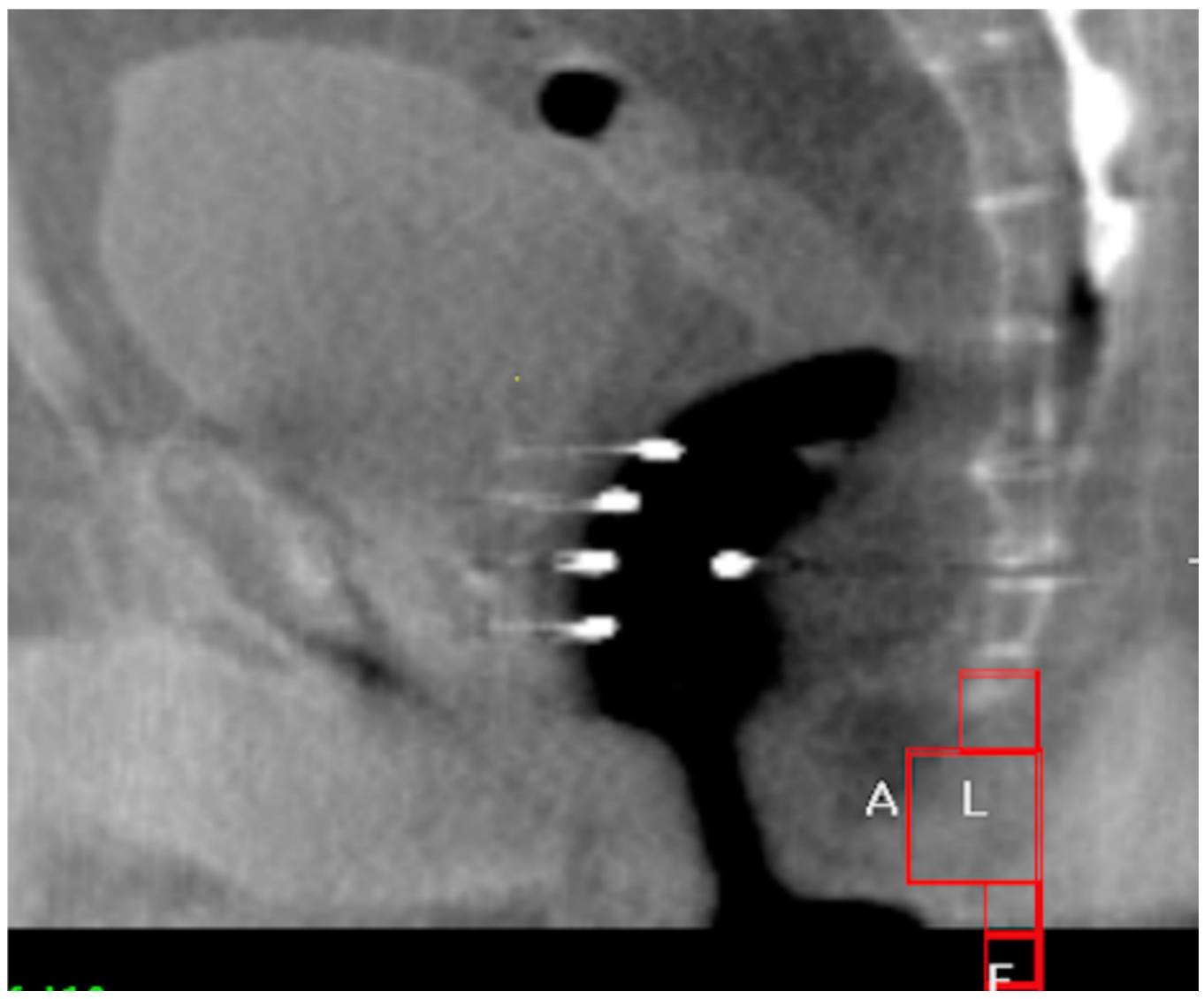

Fig 1. ProSpare™ device

Figure 2: Sagittal view on CBCT of ProSpare™

Rationale

ProSpare™, a novel rectal obturator has been designed to localize the prostate on X-ray image guidance radiotherapy and shown to enable a reduction in mean anorectal dose. Bladder filling and stool and/or air in the rectum are well known factors influencing inter and intra-fraction motion of the prostate and or prostatic bed. In prostate bed radiotherapy, there is anatomical difficulty and morbidity associated with insertion of fiducials into the prostate bed. In addition, prostate bed motion has been shown to be greatest in the A-P position and large rectal volumes are included in the treated volume, as a consequence of the posterior border of the prostate bed CTV being the anterior rectal wall. ProSpare, has three potential methods of improving accuracy and toxicity of prostate bed radiotherapy. These are as a non-invasive daily online image guided tool, hence reduction of PTV margins, a rectal spacer and prostate bed stabilizer. Therefore, we want to investigate the use of ProSpare in this patient population.

Toxicity is an important endpoint when developing a new radiotherapy technique. Patient reported outcome (PRO) is a measurement of a patient's health status that is obtained directly from the patient, without interpretation of the patient's responses by a clinician. The Institute of Medicine, the FDA and AHRQ have called for measuring PROs as primary endpoints of evidenced-based effectiveness research [18-20]. Trials have shown PROs can be more reflective of the patient experience and provide complementary and sometimes more, toxicity and symptom data than physician reported assessments [21,22].

We propose that ProSpare™ online daily image guided IMRT will improve bowel function compared with standard IMRT and therefore, the primary endpoint of this trial is patient reported outcome of bowel function and bother using the EPIC-bowel questionnaire. EPIC is a widely used validated prostate cancer health-related quality of life self-administered instrument and it has been suggested that it is the most appropriate instrument to use after localized treatments for prostate cancer [23,24].

4 Study Objectives

- **Primary**

  To compare the EPIC bowel scores for patients receiving adjuvant or salvage prostate bed radiotherapy treated with ProSpare™ image–guided IMRT versus standard arm of IMRT at 2 years following radiotherapy.

- **Secondary**

  To compare acute and late bowel and bladder toxicity and sexual function between the two study arms

  To compare quality of life in patients randomized to the two trial arms

  To evaluate biochemical progression free survival

  To analyse the dosimetric consequences of the two radiotherapy treatment techniques and relate these to toxicity endpoints

5 Study Design

This is a phase II multi-centre, open-labeled, randomized trial designed to demonstrate a difference in patient reported bowel function and bother using EPIC questionnaire between patients treated with ProSpare™ IG-IMRT with reduced margins and non-IGRT IMRT with standard margins.

Stratification

Patients will be stratified by:

- Clinical indication for prostate bed radiotherapy: adjuvant or salvage
- Treatment centre – as each centre will need to declare their institutional standard daily dose-fractionation and total dose prior to enrolment of the first patient (i.e. it will be the same for all patients), there is no need to stratify separately for dose.
- Hormone therapy
    - No androgen therapy
    - 6 months hormone therapy
    - >6 months but <24 months
    - ≥24months
- Boost given to macroscopic disease (allowed dose up to 74Gy in 2 Gy per fraction) – centres will need to declare if they would propose to give a boost to macroscopic disease at time of their enrolment.

Randomisation

Central randomisation will be performed by the Institute of Cancer Research Clinical Trials and Statistics Unit (ICR-CTSU).

The randomisation process will use minimization with a random element and with stratification factors listed above as balancing factors.

Once written informed consent has been obtained, an eligibility and randomisation checklist must be completed prior to randomisation. The clinician / research nurse should contact RM-CTU to confirm eligibility and obtain a unique trial number and treatment allocation.

The following information will be required at randomisation:

- Name of Hospital, consultant and person randomising patient
- Confirmation that patient is eligible for the trial by completion of the checklist;
- Confirmation that patient has given written informed consent;
- Patient's full name, hospital number, date of birth, postcode and NHS/CHI number
- Clinical indication for prostate bed radiotherapy
- Proposed radiotherapy dose and whether a radiation boost to macroscopic disease is going to be delivered
- Whether patient is going to receive androgen suppression and if so for how long
- Confirmation radiotherapy will commence within 8 weeks following randomisation.

The caller will be given the patient's unique randomisation number (Trial ID). The Trial ID together with the patient's initials, date of birth and hospital number should be used on all Case Report Forms (CRFs) and correspondence relating to the patient.

The treatment planning process can only begin after the patient has been randomized. Radiotherapy should commence within 8 weeks of randomization.

Study Flow Chart

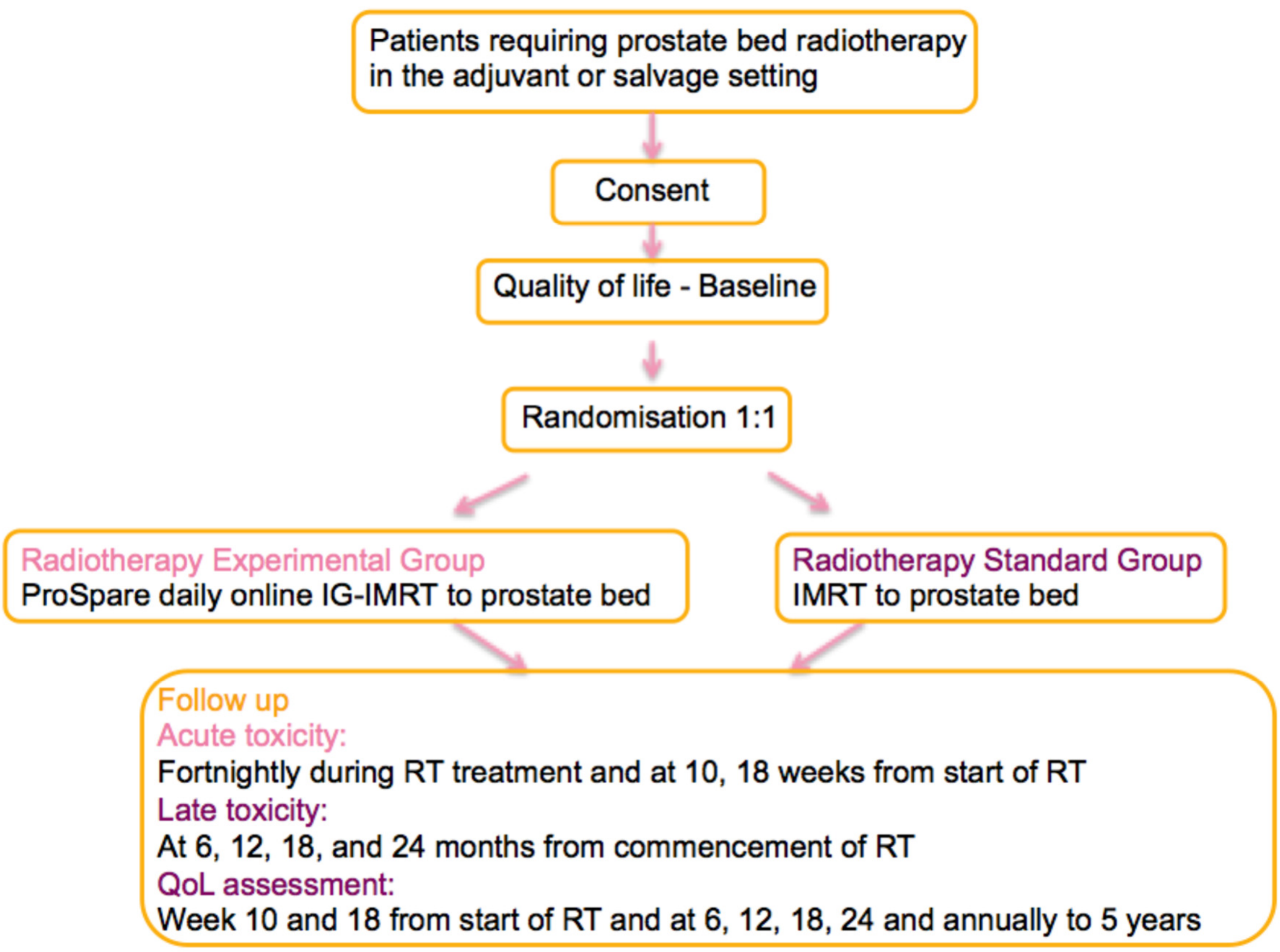

6 Study Population

Inclusion criteria:

⇒ Patient has undergone radical prostatectomy

⇒ Histologically confirmed prostatic cancer

⇒ Patient due to receive post-operative prostate bed radiotherapy either in the adjuvant or salvage* setting

* Adjuvant radiotherapy defined as planned early post-operative treatment in patients with PSA ≤0.2 ng/ml and not meeting the definition of PSA failure for salvage therapy. Salvage radiotherapy defined as post-operative biochemical failure defined as either two consecutive rises in PSA and final PSA >0.1ng/ml or three consecutive rises in PSA.

⇒ WHO performance status 0-1 at randomization

⇒ Normal anal tone assessed on rectal examination

⇒ Age ≥ 18 years

⇒ Written informed consent

⇒ Able to independently complete patient questionnaires

Exclusion criteria:

⇒ Lymph node or distant metastases from prostate cancer

⇒ Pelvic lymph node radiotherapy planned

⇒ Prior pelvic radiotherapy

⇒ Previous invasive cancer in the past 3 years, with the exception of non-melanoma skin cancer

⇒ Symptomatic haemorrhoids or other anal conditions which affect adequate insertion and retention of the rectal obturator

⇒ Planned chemotherapy within 2 months of completion of adjuvant or salvage radiotherapy

⇒ Musculoskeletal conditions which limit flexibility or mobility and will make patient self insertion of ProSpare™ difficult

⇒ Comorbid conditions likely to impact on advisability of prostate bed radiotherapy (e.g. inflammatory bowel disease, previous colorectal surgery, significant bladder instability or urinary incontinence)

7 Study Treatment and Procedures

ProSpare™

ProSpare™ II devices will be manufactured by Europlaz Technologies Limited. Europlaz maintain a quality management system to the requirements of ISO13485:2012, including requirements for product identification and traceability. To ensure traceability of ProSpare™ devices, each batch of ProSpare™ II devices produced will be identified with unique lot numbers, which will be recorded by the centres on case report forms. The first two batches of devices (2000 devices in total) will be delivered from Europlaz to the Royal Marsden, Clinical Trials Unit (Sutton) and released by the CTU to each investigation site following confirmation that all required site approvals are in place. Europlaz deliveries to all sites will be accompanied by a delivery note detailing the batch lot numbers and investigation site, and a copy of this delivery note will be sent to the Royal Marsden CTU. A training programme will be undertaken by investigator sites. The aim of this training programme is to educate centres and patients in the use of ProSpare™ and ensure that ProSpare™ is being used appropriately as a daily online image guidance tool. This training will include written guidelines, contouring atlas, a Webinar and practice cases. This will be co-ordinated by the research radiographer at The Royal Marsden NHS Foundation, who will also be a point of contact for other centres regarding radiotherapy issues, gain feedback on centres experience of using ProSpare™ and revise procedures appropriately.

Hormonal treatment

The decision to treat patients with androgen suppression will be decided by the treating centre. Prior to randomization, this decision and duration of treatment needs to be confirmed.

Radiation treatment

All patients will be treated with inverse planned IMRT to the prostate bed and radiotherapy will start within approximately 8 weeks following randomization, to allow sufficient time for IMRT planning. Patient will either receive daily online ProSpare™ IGRT or centre-standard IGRT. The target volumes are to be outlined according to the RTOG guidelines[25]. Treatment will be daily and the dose given to the prostate bed can be decided by the centre. If there is evidence of macroscopic prostate bed recurrence, a radiation boost can be given within the trial.

Patient's positioning and preparation

⇒ All patients will have their planning CT scan and radiotherapy treatment in the supine position with arms displaced out of the radiotherapy field, using local centre pelvic immobilization techniques.

⇒ Patients will be provided with bladder filling instructions and rectal preparation information as per local unit policy. It is recommended that the bladder should be comfortably full, (patients to drink approximately 350 ml during the hour pre scan) and the rectum should ideally be empty of both faeces and flatus; the routine use of micro enemas (e.g. microlette) is permissible.

⇒ Patients randomized to the ProSpare™ IG-IMRT must self-insert the ProSpare™ device for the planning CT scan.

Simulation (patient data acquisition) guidelines

⇒ A volumetric treatment planning CT will be required to define target volumes and organs at risk and the CT scans will be taken at ≤2.5mm intervals (≤2.5mm slice thickness) from the bottom of the sacro-iliac joints to a point below the level of penile urethra (usually 1cm below ischial tuberosities will be adequate).

⇒ If the anterior-posterior diameter of the rectum is >4cm at any level within the superior and inferior aspect of the pubic symphysis, the patient should be rescanned.

Definition of treatment volumes

As IMRT techniques are to be used in this trial, the definition of volumes and the dose reporting shall be in accordance with the ICRU report 83.

Gross Tumour Volume (GTV): macroscopic prostate bed recurrence identified on functional MRI. The majority of patients will not have a GTV outlined, as either they would not have met the clinical criteria to justify a functional MRI scan or no macroscopic disease recurrence was identified on imaging.

Clinical Target Volume: prostate bed only

The definition of the CTV is based on (not exclusive):

- Pre and postoperative transrectal ultrasound, CT/MRI scans or PET/PET-CT scans, if available.
- The histophathological assessment of the prostate including the size, areas of extraprostatic extension, seminal vesicle invasion or positive margins.
- The surgical clips when they are present.
- The information available at the time of treatment planning, which requires in particular reference to the estimated position of the vesico-urethral anastomosis.

The CTV is defined as all areas considered to harbor microscopic disease and which are at the greatest risk for relapse after prostatectomy using the planning CT images and clinical information.

For this study, CTV is defined according to the RTOG consensus guidelines. The full CT data set with each contoured slice can be found on the RTOG website (www.RTOG.org).

See Appendix A for 2 examples of consensus contours for a) patient with apical positive margins and b) patient with extracapsular extension at base and seminal vesicle involvement.

Anatomical borders of postoperative clinical target volume for prostate cancer (RTOG consensus guidelines[25])

	Below the superior edge of the symphysis pubis	Comments
Anterior	Posterior edge of pubic bone	
Posterior	Anterior rectal wall	May need to be concave around lateral aspects
Inferior	8–12 mm below vesicourethral anastomosis	May include more if concern for apical margin. Can extend to slice above penile bulb if vesicourethral anastomosis not well visualized
Lateral	Levator ani muscles, obturator internus	
	**Above the superior edge of the symphysis pubis**	
Anterior	Posterior 1–2 cm of bladder wall	
Posterior	Mesorectal fascia	
Superior	Level of cut end of vas deferens or 3–4 cm above top of symphysis	Vas may retract postoperatively; include seminal vesicle remnants if pathologically involved
Lateral	Sacrorectogenitopubic fascia	If concern about extraprostatic disease at base may extend to obturator internus

Planning Target Volume

The PTV will provide a margin around the CTV to compensate for the variability of treatment set up and internal organ motion.
For this study, the two arms will have different CTV – PTV1 margins. For patients receiving centre-standard IGRT, CTV to PTV margins will be centre-defined. . In the ProSpare™ daily IGRT arm the PTV1 is defined as CTV using data from analysis in intact prostate patients. These margins may be modified in the future when data from the pilot study in post-prostatectomy patients are available. The ProSpare™ daily IGRT PTV margin is defined as CTV + 10mm superior/inferior/right/left/anterior and 5mm posteriorly.

The ProSpare™ PTV should be grown as follows:

		Sup	Inf	Right	Left	Ant	Post	Excluding structure
CTV to PTVa	Margin (mm)	5	5	5	5	5	0	Rectum
PTVa to PTV1	Margin (mm)	5	5	5	5	5	5	None

For those patients where a GTV has been outlined, the GTV-PTV margin will be a 5mm intraprostatic bed margin.

Organs at Risk (OAR)

Normal Structures to be outlined include:

- Bladder
- Urethra
- Anus
- Rectum
- Sigmoid, Large Bowel and Small bowel
- Right femoral head
- Left femoral head
- Penile Bulb

Bladder

- The outside of the bladder wall should be outlined. The entire bladder should be included.

Urethra

- The urethra will be outlined using information from all available imaging. It will be outlined from bladder neck to start of penile urethra. Median dose to the urethra will be recorded.

Anal Canal

- The anal canal is defined as from the anal verge (approximately bottom of ischium) extending 3 cm cranially. The circumference and contents should be fully outlined.

Rectum

- The rectum is outlined to include the full circumference and rectal contents. Outlining should extend from the anal verge to the rectosigmoid junction. This overlaps with the delineated anal canal but the rectum must be outlined in full as the validated dose-volumes constraints used to restrict toxicity include the lower 1/3 (anorectum). At the rectosigmoid junction, the rectum and sigmoid will be outlined as different structures. The rectosigmoid junction will be defined as the level at which there is an anterior inflection of the bowel – this is usually best appreciated on sagittal reconstructions on the CT planning scan.

Sigmoid and Bowel

- Sigmoid, large and small bowel should be outlined as the same structure “bowel” unless it is felt possible to differentiate. Attention should be paid to following the lumen(s) over consecutive scan slices. Following the recto-sigmoid junction the sigmoid colon becomes increasingly anterior and lies superior to the bladder, sagittal views are often helpful. The combined volume (sigmoid, large bowel and small bowel) will be used for the bowel dose-volume constraint. The superior extent of outlining should be 2cm beyond the superior extent of PTV prostate bed.

Right and Left Femoral Heads

- The femoral heads are outlined to the bottom of the curvature of their heads (femoral necks are not included).

Penile bulb

- The penile bulb is defined as the portion of the bulbous spongiosum of the penis immediately inferior to the genito-urinary diaphragm. The structure should not be extended anteriorly into the shaft or pendulous portion of the penis. The penile bulb is limited by the crura laterally, the corpora spongiosum anteriorly and the levator ani posteriorly. When contouring, the anterior border of the penile bulb in the more caudal slices can be arbitrarily defined as the projection of the penile bulb anterior border of the most cranial slice, where this border is more visible.

External Beam Equipment and Technique

⇒ Megavoltage equipment with nominal photon energies $\geq$6MV are required.

⇒ IMRT will be performed using the local planning system using multiple beams to obtain a uniform coverage of the PTV and satisfy the dose constraints to the OARs.

⇒ Centres may employ a variety of different IMRT delivery techniques including 'step and shoot" or "dynamic' MLC movement or rotational techniques, such as tomotherapy, RapidArc, IMAT, VMAT.

⇒ The method of treatment planning and delivery will vary according to centre and these must be approved in advance by the trial management group.

⇒ The use of multileaf collimators is mandatory for complex field shaping.

Dose prescription, recording and reporting

Dose prescription

Dose examples given, PTV dose dependent on centre, this will be reported prior to first patient randomization.

⇒ **PTV prostate bed only:**

- The recommended prostate bed prescribed dose is 66Gy in 33 fractions over 6.5 weeks. Patients will receive treatment 5 days per week, in a once daily fraction. All fields and the entire PTV must be treated daily. The target doses used within this trial is at the discretion of the local centres, and alternative schedules may be permissible but should be agreed with the Chief Investigator and TMG. Schedules must be the same in each randomised group.
- If the prescription dose is 66Gy, this shall be the dose to the PTV and the following dose objectives will be met: D98% $\geq$ 62.7Gy, D50% = 66Gy ± 1%, D2% $\leq$ 70.6Gy (aim for D2% < 69.3 Gy).

⇒ Dose limits for PTV

% PTV	% Dose
99	>90
95	>95
≤5	105
<2	107

⇒ **PTV prostate bed and macroscopic disease boost***:*

The recommended prostate bed and boost dose is 70Gy in 37 fractions to the prostate bed and 74Gy in 37 fractions over 7.5 weeks to the boost. Patients will receive treatment 5 days per week, in a once daily fraction. All fields and the entire PTV must be treated daily. The target doses used within this trial is at the discretion of the local centres, and alternative schedules may be permissible but should be agreed with the Chief Investigator and TMG. Schedules must be the same in each randomised group.

	**Dose Prescribed (Gy)**	**Minimum Dose (Gy)**	**Fractions (#)**	**Dose per # (Gy)**	**EQD2 (Gy) α/β =3**
**PTV 66**	**66**	**62.7**	**37**	**1.78**	**63.15**
**PTV 70**	**70**	**66.5**	**37**	**1.9**	**68.49**
**PTV 74**	**74**	**70.3**	**37**	**2**	**74**

Minimum dose is defined as the D98% in line with recommended reporting in ICRU 83. The maximum dose is defined as the D02%, with a mandatory constraint of <77.7Gy (105% of 74Gy) and an optimal objective of <76.22Gy (103% of 74Gy) for PTV74. Median dose to PTV boost should be boost dose in Gy ± 1%; for PTV prostate bed the median dose should not be less than the prescription dose for that PTV.

Dose recording

These will be recorded on a case report form.

- Target dose achieved: dose to 98% of PTV, dose to 50% of PTV and dose to 2% of PTV.
- Hot spot outside the PTV.
- Dose to organs at risk.

Dose volume constraints to organs at risk

Dose volume constraints will be as shown in the table. Literature suggests that these will limit the incidence of late toxicity for conventional fractionation.

Organ at risk	Dose-volume constraints		
		Optimal	Mandatory
Anus	Dmean	30Gy	-
	V35	60%	-
	V40	40%	-
	V60	0%	-
Rectum	V30		80%
	V40		70%
	V50	50%	60%
	V60	35%	50%
	V65	30%	30%
	V70	15%	15%
	V75	3%	5%
	D02	-	76 Gy
Bladder	V50		80%
	V60		50%
	V70	5%	35%

	V75	3	25%
	V80	0.2%	15%
Sigmoid, Small & Large Bowel	V45	78cc	158cc
	V50	17cc	110cc
	V55	14cc	28cc
	V60	0.5cc	6cc
	V65	0cc	0cc
Femoral Heads	V50	10%	-
	V50	-	50%

Radiotherapy Delays

Delays in radiotherapy should be avoided. If there is a treatment delay greater than one week due to factors not related to toxicity (e.g. Machine breakdown), then consideration may be given to treating patients at the weekend. If a treatment gap occurs a trial deviation form should be completed to record the details of the delay. Under no circumstances should patients be treated twice in one day.

Treatment Verification

Non-daily IGRT patients

Minimum treatment verification requirements are for appropriate offline imaging protocol e.g. no-action level protocol. This is imaging 3 times in week 1 of treatment and correcting for any systematic error. Subsequently, imaging at least weekly, using offline corrections and a 3mm action level. If the displacement is >3mm then repeat imaging for 3 days and determine new systematic error. An alternative is to use an extended no-action level protocol and determine a new systematic error each imaging day. Treatment centres can use their own standard treatment verification schedule as long as it meets the minimum requirements.

ProSpare™ daily IGRT patients

Treatment verification will take place using ProSpare™ ball bearings and daily online cone beam CT (CBCT). CBCT will be taken following patient set-up to laser and skin markers. The images will be reconstructed and registration to ProSpare™ ball bearings will be automatic. The match will then be reviewed by the treatment radiographers to confirm a satisfactory match. When

satisfied the radiographers will auto-correct the treatment couch to align the patient to the reference image. A radiotherapy treatment work instruction document will be given to each treatment centre.

Clinical evaluation, laboratory tests, follow-up

Pre-randomisation investigations

- Clinical history and physical examination
- Review of prostatectomy histopathology report
- Full blood count and biochemistry to include creatinine, alkaline phosphatase, testosterone
- PSA
- ProSpare™ tolerability assessment
- Symptom assessment of bowel, urinary symptoms and potency should be recorded using the National Cancer Institute Common Terminology Criteria for Adverse Events (NTI CTCAE) version 4 and Gulliford rectal scoring system
- Patient Reported Outcomes should be completed within 2 weeks prior to randomization using the patient completed EPIC, modified IBDQ, Vaizey, IPSS, IIEF-5 and SF-12

Pre-Treatment

Within 2 weeks prior to commencement of radiotherapy:

- PSA value
- Full blood count and biochemistry to include creatinine, alkaline phosphatase, testosterone
- ProSpare™ tolerability questionnaire (pre-treatment)
- Symptom assessment of bowel, urinary symptoms and potency should be recorded using the National Cancer Institute Common Terminology Criteria for Adverse Events (NTI CTCAE) version 4 and Gulliford rectal scoring system
- Patient Reported Outcomes should be completed within 2 weeks prior to randomization using the patient completed EPIC, modified IBDQ, Vaizey, IPSS, IIEF-5 and SF-12

Assessment During Radiotherapy Treatment

Patients will be seen fortnightly during radiotherapy treatment. Acute toxicity will be assessed using the using a modified Radiation Therapy Oncology Group (RTOG) scoring system (Appendix) during:

Week 2 from start of radiotherapy (corresponding to fractions 5-10)

Week 4 from start of radiotherapy (corresponding to fractions 15-20)

Week 6 from start of radiotherapy (corresponding to fractions 25-30)

Week 8 from start of radiotherapy

ProSpare™ tolerability questionnaire will be given at week 8 visit for patients to complete on completion of radiotherapy and to be collected at week 10 visit

Immediate Post-Radiotherapy Safety Follow-Up

Week 10 from start of radiotherapy:

- Toxicity assessment with RTOG scoring system

Week 18 from start of radiotherapy:

- Toxicity assessment with (using RTOG, Gulliford and NCI CTCAE v4 scoring systems)
- Patient Reported Outcomes questionnaire (EPIC, Modified IBDQ, Vaizey, IPSS, IIEF-5 and SF-12).

Post-Radiotherapy Treatment Follow-Up

At 6, 12, 18 and 24 months from commencement of RT:

- PSA test
- Late side effect safety assessment (using RTOG, Gulliford and NCI CTCAE v4 scoring systems)
- Patient Reported Outcomes will be evaluated using the EPIC, modified IBDQ, Vaizey, IPSS, IIEF-5 and SF-12 questionnaires either given to the patient in clinic or sent electronically.
- Testosterone levels will be measured 12 months after completion of androgen suppression and at 6 monthly intervals until normalization of testosterone levels or pre-hormone

treatment level is reached, whichever is lower. If the patient was not on androgen suppression, they will have one measurement at month 12 post radiotherapy.

Assessment of Disease Recurrence

Full assessment of disease will be undertaken if there is significant clinical or biochemical evidence of disease recurrence. This will include CT or MR of the pelvis and bone scan. To trigger re-evaluation there should be clinical evidence of recurrence or PSA levels should be ≥0.2 ng/mL above post-RT nadir.

Summary table of clinical evaluation assessments

Visit				Week from start of radiotherapy						Month			
Time from radiotherapy start date	**Prior to Enrolment**	**Prior to Randomisation**	**Prior to radiotherapy**	**2**	**4**	**6**	**8**	**10**	**18**	**6**	**12**	**18**	**24**
Clinical history & physical examination	X									X	X	X	X
Pathology review	X												
Trial insertion of ProSpare™ in clinic	X												
ProSpare™ tolerability questionnaire	X												
Haematology and biochemistry and NON-fasting glucose[15]	X		X										

Time from radiotherapy start date	**Prior to Enrolment**	**Prior to Randomisation**	**Prior to radiotherapy**	**2**	**4**	**6**	**8**	**10**	**18**	**6**	**12**	**18**	**24**
PSA	X		X					X	X	X	X	X	X
Testosterone [16]	X		X								X		
RTOG toxicity assessment (3)				X	X	X	X	X	X	X	X	X	X
Gulliford Rectal Score (4)	X		X						X	X	X	X	X
NCI CTCAE v4 toxicity assessment (4)	X		X						X	X	X	X	X
Patient Reported Outcomes assessment (5)	X		X						X	X	X	X	X*
ProSpare™ User Questionnaire			X				X						

1. Full blood count and biochemistry to include creatinine, alkaline phosphatase, testosterone.
2. Testosterone levels: pre-hormone testosterone level should be reported if available and testosterone will be measured at 12 months from radiotherapy if not on androgen suppression. If on androgen suppression, testosterone levels will be measured 12 months after completion of androgen suppression.
3. Acute RTOG toxicity scales should be used to assess side effects up to and including week 18.
4. CTCAE v4 and Gulliford Rectal Scores will be added at baseline and at week 18. Late toxicity will be scored from month 6 using RTOG, Gulliford and CTCAE v4 scales.
5. Instruments used will be the EPIC, modified IBQD, IPSS, IIEF and SF-12 questionnaires. Questionnaires will be administered at clinic visits or posted to patients.

*After month 24, patients will receive by post the patient reported outcomes assessments annually at month 36, 48 and 60.

8 Criteria of Evaluation

Primary Endpoint

- Patient reported EPIC bowel function and bother at 2 years after prostate bed radiotherapy.
- This will be presented as the mean difference in scores between the two groups.

Secondary Endpoints

⇒ Acute bowel and bladder toxicity at week 18 from start of radiotherapy

- Prevalence of patients with lower GI/rectal or GU toxicity at week 18 from start of radiotherapy. This will be assessed using RTOG, CTCAE v4, Gulliford Rectal scoring systems, EPIC and IPSS. Focus will be given to occurrence of grade $\geq$2 toxicity.

⇒ Late (1 and 2 year) bowel and bladder toxicity

- Late toxicity will be assessed using EPIC, RTOG, CTCAEv4, Gulliford Rectal Score, EPIC, IPSS. RTOG and CTCAEv4 Grade 2 ≥ will be assessed for both GU and lower GI / rectal. Peak and cumulative levels of toxicity will be measured. Cumulative incidence is defined as proportion of patients that suffered relevant toxicity within the first 12 and 24 months over number evaluable at 12 and 24 months respectively. Prevalence will be defined as the proportion of patients with relevant late toxicity at 12 and 24 months from start of radiotherapy over the number of evaluable at 12 and 24 months respectively. Individual grades of toxicities at each time point will be tabulated.

⇒ Sexual function

- Sexual function will be assessed using physician and patient reported data with CTCAEv4 and IIEF-5 respectively at baseline and at 12 and 24 months.

⇒ Patient reported outcomes

These will be evaluated using the patient completed EPIC, modified IBQD, Vaizey, IPSS and SF-12. Patient reported outcomes will be assessed at baseline (pre-enrolment and pre-radiotherapy treatment), at week 18 and at 6,12,18, 24 months and annually up to 5 years.

- EPIC assesses the disease-specific aspects of prostate cancer and its therapies and comprises four summary domains: Urinary, Bowel, Sexual and Hormonal. Response options for each EPIC item form a Likert scale, and multi-item scale scores are transformed linearly to a 0 -100 scale with higher scores representing better health-related quality of life. For the overall urinary, bowel, and sexual bother questions, they are scored on a 5 point scale (no problems, very small problem, small problem, moderate problem or big problem). The responses will be grouped into 3 clinically relevant categories (no problem, very-small to small problem, and moderate to big problem).

- The total modified IBDQ score is calculated by summing together the scores for each of the 32 items. The lowest possible score is 32 and indicates the most severe symptoms; the highest possible score is 224 and indicates an asymptomatic patient. There are four subscales within the IBDQ; bowel (10 items), emotional (12 items), systemic (5 items) and social (5 items). Median scores will be presented.

- The Vaizey questionnaire consists of 7 questions and will be summarised as a total score ranging from 0 (no problems with bowel continence) to 24 (very severe problems with incontinence); see table 1. At each assessment time point the total Vaizey score will be presented as a median, IQR and range by treatment group. Only patients with complete Vaizey questionnaires will be included in the total score analysis at each time point. The proportion of patients defined as ‘sometimes’, ‘weekly or ‘daily’ will be summarised as

frequency and percentage for each individual question at each time point and by treatment group. Data will also be displayed graphically as stacked bar charts. If clinically significant differences are evident then a statistical comparison may be made at the 2 year time point using a one-sided 1% significance level.

- Urinary symptoms are reported by patients using the International Prostate Symptoms Score (IPSS) questionnaire collected pre-radiotherapy, week 10 (expected peak toxicity), week 18 and then at 6, 12, 18 and 24 months. The IPSS consists of 7 questions each graded from 0 (no problems) to 5 (always a problem) with a total score calculated from these 7 items and coded as mildly symptomatic (0-7); moderately symptomatic (8-19) or severely symptomatic (20-35). Median total score will be reported and frequency of response to each question tabulated.

- Inter-scale correlation between EPIC and Medical Outcomes Study 12-item Short Form Health Survey (SF-12) have indicated that efficient yet comprehensive health related quality of life assessment can be achieved by co-administering EPIC with SF-12. SF-12 produces two summary scales assessing physical function and mental well-being.

⇒ Biochemical progression free survival

- Defined as ≥0.2 ng/mL above post-radiotherapy nadir.

⇒ Dosimetric assessment of prostate bed and rectal dose with and without ProSpare

- Dosimetric assessment of prostate bed and rectal dose with and without ProSpare will take place using dose volume histograms (DVHs) to directly compare the volumes of rectum receiving 20-74 Gy, Collection of dose cube information for the modeling of normal tissue complication effects will include detailed dose volume and surface histogram analysis. Rectal sparing will be

defined as the proportion of patients who have an improvement in DVHs or NTCPs. Further exploratory dosimetric assessments will be made including the assessment of rectal dose-surface maps.

⇒ Tolerability will be defined as the patients being able to complete treatment satisfactorily and without significant discomfort. It will be assessed with a yes /no question in a short questionnaire at the end of treatment.

Exploratory Endpoints

⇒ To compare physician reported toxicity with patient reported data
    - Physician rated symptoms will be paired with comparable and temporally matched patient reported quality of life domains to determine level of agreement.

9 Study Organisation, Trial Monitoring and Management Strategy

Centres wishing to recruit to this study will be asked to provide evidence that they can deliver protocol treatment. This will include the successful completion of the POPS QA program.

Prior to commencing trial recruitment, training will be provided to identify key individuals in each participating network by the Chief Investigator and Trial Management Group. Training will include discussion on the background to the study, evidence for potential benefits and drawbacks of ProSpare daily online IGRT and discussion on the issues of clinical equipoise. Experience developed from successfully recruiting centres and information from associated studies will be provided to participants at their initial training and subsequently on a regular basis. Participating centres will be expected to join regular (≤3 monthly) teleconferences with the Trial Management Group to discuss trial progress and identify any recruitment, treatment planning and delivery difficulties and maintain standards determined by the Quality Assurance Group.

♦ Staff will comply with the Standard Operating Procedures for POPS trial.

- RM-CTU will monitor receipt of CRFs. They will also check incoming CRFs for compliance with the protocol, inconsistent and missing data.
- RM-CTU will contact centres to discuss dates of any proposed monitoring visits. Once a date has been confirmed a list of names of patients whose notes will be monitored during the visit will be sent to the centre. This list will be sent out in advance to give sufficient time for the notes to be made available. Site monitoring will usually be conducted at participating centres at least once during the first year following entry of the first patient. It is likely that a random sample of notes will be selected for limited source document verification.

Regulatory issues

Good Clinical Practice

The study will be conducted in accordance with the conditions and principles of GCP as defined in ISO14155:2011 clinical investigations of medical devices for human subjects. Good clinical practice.

Initial Approval

Before starting the trial, the protocol, patient information sheet, consent form, and any other written information that will be provided to the patients and any advertisements that will be used and details of any patient compensation must be approved by the Royal Marsden/Institute of Cancer Research joint Committee for Clinical Research. Once approved, the study will then be submitted to the relevant Ethics Committee (REC) for their review and approval and Medicines and Healthcare products regulatory agency (MHRA). The trial will not begin until favourable REC opinion and notice of no objection from MHRA.

Prior to the shipment of devices and the enrolment of any patients the Investigator at each site is responsible for any site-specific assessments and obtaining applicable local approvals for the study. All participating sites will be required to sign an agreement with RM-CTU which includes requirement to sign and adhere to the trial protocol.

Approval of Amendments

Any protocol amendment should be agreed with the trial management group (TMG) and be approved by the sponsor prior to submission and review by the relevant Ethics Committee. Once favourable opinion from IEC has been obtained the amendment can be distributed to sites

and implemented. It is the responsibility of the Chief Investigator to submit amendments to Sponsor approval. Amendments requiring REC approval may be implemented only after a copy of the REC/RA's approval letter has been obtained. Amendments that are intended to eliminate an apparent immediate hazard to patients may be implemented prior to receiving Sponsor or REC/RA approval. However, in this case, approval must be obtained as soon as possible after implementation.

Regulatory Authority Approval

The study will be performed in compliance with UK regulatory requirements. A clinical investigation application and notice of no objection from the Medicines and Healthcare products Regulatory Authority (MHRA) will be obtained prior to the start of the study. In addition, the MHRA must approve amendments (as instructed by the Sponsor), receive SAE reports and be notified of the end of the trial.

Insurance and Liability

The Sponsors have secured indemnity from the manufacturer of ProSpare for patients in relation to adverse side effects for device-induced injury. Indemnity for participating hospitals is provided by the usual NHS indemnity arrangements for clinical negligence. A copy of the relevant insurance policy/indemnity scheme or summary shall be provided on request.

Contact with General Practitioner (GP)

It is the Investigator's responsibility to inform the patient's GP by letter that the patient is taking part in the study provided the patient agrees to this, and information to this effect is included in the PIS and ICF. A copy of the letter should be filed in the Site File. A template letter approved by the REC/RA will be provided by the Sponsor to all participating sites.

Patient Confidentiality and Data Sharing

The Chief investigator must ensure that the patient's confidentiality is maintained in compliance with the UK Data Protection Act of 1998. On the e-CRFs or other documents submitted to the RM-CTU, patients should be identified by their initials and a patient study number only.

In compliance with GCP guidelines, it is required that the investigator and institution permit authorised representatives of the sponsor and of the regulatory agency(s) direct access to review the patient's original medical records for verification of study-related procedures and

data. Direct access includes examining, analysing, verifying, and reproducing any records and reports that are important to the evaluation of the study. The investigator is obligated to inform and obtain the consent of the patient to permit named representatives to have access to his/her study-related records without violating the confidentiality of the patient.

Data collection and documentation

It is the Investigator's responsibility to ensure that all relevant data is clearly recorded in the medical records. The Investigator must allow the RM-CTU direct access to relevant source documentation for verification of data entered into the e-CRF, taking into account data protection regulations. The clinical data should be recorded in the e-CRF and the must be verifiable by the source data.

The investigator is not allowed to deviate from the trial protocol, procedures for recording, reporting and analyzing clinical investigation plan deviations, notification requirements and timeframes.

The patients' medical records, and other relevant data, may also be reviewed by appropriate qualified personnel independent from the sponsor appointed to audit the trial, or by REC. Details will remain confidential and patients' names will not be recorded outside the hospital.

The Principal Investigators at each centre are confirming agreement with his/her local NHS Trust to ensure that

- correct storage of the device, ProSpare™ and documentation of the batch lot numbers used by each patient
- sufficient data is recorded for all participating patients to enable accurate linkage between hospital records and e-CRFs
- source data and all trial related documentation are accurate, complete, maintained and accessible for monitoring and audit visits
- original consent forms are dated and signed by both patient and investigator and are kept together in a central log together with a copy of the specific patient information sheet(s) given at the time of consent
- all essential documents must be retained after the trial ends to comply with current legislation

No study document will be destroyed without prior written agreement between the Sponsor and the PI. Should the PI wish to assign the study records to another party or move them to another location, written agreement must be obtained from the Sponsor.

Electronic Recording of Data

Patients' data will be documented on a trial specific e-CRF designed by RM-CTU. Upon signing the informed consent form, the patient is assigned to the next sequential Patient screening number available. Following confirmation of eligibility patient will be randomized and assigned a randomization number.

The Investigator is responsible for ensuring the accuracy, completeness, clarity and timeliness of the data reported in the e-CRFs. Only the Investigator, and those personnel who have completed the Study Team Responsibilities Signature Log/Delegation Log as authorised by the PI, should enter or change data in the e-CRFs. All protocol required investigations must be reported in the e-CRF. The Investigators must retain all original reports, traces and images from these investigations for future reference. The data will be entered in a clinical trials database (Macro V4). If a patient withdraws from the study, the reason must be noted on the e-CRF.

Authorised site personnel must not enter study-specific data directly into e-CRFs and ensure all results are appropriately documented in the patients' medical records. The e-CRF will be signed electronically by the Investigator or by an authorised staff member. Study specific information will be entered into an e-CRF visit by visit. Data that are derived should be consistent with the source documents or the discrepancies should be explained. All e-CRF data should be anonymous, i.e. identified by study patient number only.

Data Management

Data management will be carried out by RM-CTU using an electronic database and in accordance with the data management plan agreed by the RM-CTU and RDSU. Data entry will be carried out by appropriately trained personnel at participating centres. Queries will be raised centrally by the trial manager / trial monitor and sent to the participating centre for resolution.

Data Acquisition

Electronic (e) Case Report Forms (CRF) will be used for the collection of trial data. RM-CTU will provide guidance to sites to aid the completion of the eCRFs. The Trial Management Group reserves the right to amend or add to the eCRF template as appropriate. Such changes do not constitute a protocol amendment, and revised or additional forms should be used by sites in accordance with the guidelines provided by RM-CTU.

Central Data Monitoring

Once data has been entered on the eCRF by the site personnel, RM-CTU will review it for compliance with the protocol, and for inconsistent or missing data. Should any missing data or data anomalies be found, queries will be raised for resolution by the site.

Any systematic inconsistencies identified through central data monitoring may trigger an on-site monitoring visit.

End of Trial

The end of the trial is defined as the last patient's last visit.

Study Management Structure

Delegations of Responsibilities

This trial is sponsored by the Institute of Cancer Research, London. This trial will be conducted in accordance with the professional regulatory standards required for non-commercial research in the NHS under the research governance framework for health and social care and good clinical practice. The following responsibilities have been delegated to:

RM-CTU

RM-CTU has overall responsibility for facilitating and coordinating the conduct of the trial and is also responsible for collating data obtained, and undertaking and reporting all analyses.

The responsibilities of RM-CTU for the day-to-day management of the trial will include the following;

- ensuring an appropriate ethics opinion has been sought, and any amendments have been approved

- giving notice of amendments to protocol, make representations about amendments to the Main REC
- and MHRA as applicable
- notifying sites and Sponsor that the trial has ended
- randomising patients
- raising and resolving queries with local investigators
- keeping records of all serious adverse events (SAEs), reported by investigators
- notifying the Main REC, MHRA and Investigators of related Serious Adverse Events

ProSpare™ manufacturer

Provision of ProSpare™ and product liability insurance

Participating Sites

- Putting and keeping in place arrangements to adhere to the principles of GCP
- Keeping a copy of all 'essential documents' (as defined under the principles of GCP) and ensuring appropriate archiving of documentation once the trial has ended
- Taking appropriate urgent safety measures
- Centres wishing to recruit to this study will be asked to provide evidence that they can deliver protocol treatment.
- Responsibilities are defined in an agreement between an individual participating centre and RM-CTU, which must be signed and in place before recruitment can commence.
- The Investigator is responsible for keeping accurate accountability records for ProSpare™ including the number of devices, lot number and expiry date of each device used for each patient and the amount remaining on site at the conclusion of the trial.
- Upon completion or termination of the study, it is the Investigators responsibility to ensure all expired devices will be destroyed at the site per local guidelines and unused returned the Chief Investigator.

Protocol compliance and amendments

All participating sites will be required to sign an agreement with RM-CTU, which includes requirement to sign and adhere to the trial protocol.

Any protocol amendment should be agreed with the trial management group (TMG) and be approved by the sponsor prior to submission and review by the relevant Ethics Committee and the MHRA where required. Once a favorable opinion from REC and if applicable the MHRA has been obtained the amendment can be distributed to sites and implemented. It is the responsibility of the Principal Investigator to submit amendment to their R&D department for R&D approval.

Trial Management

The RM-CTU will be responsible for the day-to-day coordination and management of the trial. This includes all duties relating to safety reporting. A trial agreement will be signed between the site and RM-CTU. Once all relevant trial approvals are in place an initiation (visit or teleconference) will be conducted. In addition, training and ongoing advice will be provided by trial training workshop(s), site initiation and ongoing site support to each participating site by Trial Management Group (TMG).

Trial Management Group

A Trial Management Group (TMG) will be set up and membership will include Chief Investigator, Chief Co Investigator, Trial Statistician and Trial Manager. Principal Investigators and other key study personnel will be invited to join the TMG as appropriate. The TMG have operational responsibility for the conduct of the trial.

Monitoring

During the trial RM-CTU is responsible for monitoring data quality in accordance with relevant standard operating procedures (SOPs). Incoming data will be monitored for protocol compliance and if any inconsistent or missing data is identified queries will be sent to the site for resolution. Any systematic inconsistencies may trigger an on-site monitoring visit.

The trial statistician will periodically examine the data for anomalies and outliers, such as too few or too many events. Queries will be raised by the trial coordinators in such situations and communication with the clinical teams will take place. In addition statistical monitoring of unusual dates and inconsistent data will take place. Again these will raise queries via the trial coordinators.

If an on-site monitoring visit is required, RM-CTU will contact the site to agree convenient date. The site must ensure that relevant site file and patient notes are available for review. RM-CTU staff conducting onsite monitoring will review the investigator site file and carry out source data verification to confirm compliance with the protocol, trial agreement. If any problems are detected during the course of the monitoring visit, RM-CTU will work with the Principal Investigator or delegated individual to resolve issues and determine appropriate action.

Quality Control and Quality Assurance

Quality Control (QC) will be performed according to RM-CTU internal procedures. The study may be audited by a Quality Assurance (QA) representative of the Sponsor. All necessary data and documents will be made available for audit.

Clinical study report

Clinical data will be presented at the end of the trial based on final data listings. The CI,/designee together with the trial statistician will prepare a brief clinical study report / publication based on the final data listings. A summary of the report must be provided to the Research Ethics Committee and the MHRA within 1 year from the submission of the end of trial notification.

Record retention

Essential documents are documents that individually and collectively permit evaluation of the conduct of the trial and substantiate the quality of the data collected. During the clinical trial and after trial closure the Investigator must maintain adequate and accurate records to enable both the conduct of a clinical trial and the quality of the data produced to be evaluated and verified in accordance with current legislation.

RM-CTU will maintain essential documents to facilitate the management of the trial, audit and inspection in accordance with RM G-SOPs and in compliance with the clinical trial regulatory requirements. The medical files of trial subjects shall be retained in accordance with national legislation and in accordance with the maximum period of time permitted by the hospital, institution or private practice. Records will be retained for the Regulatory Authority (MHRA) for a period of 5 years from the date of manufacture of the last product in accordance with the required regulations.

Reporting and publication

The trial results will be submitted for publication in a relevant medical journal with authorship according to the criteria defined by the ICMJE (http://www.icmje.org). These state that: Authorship credit should be based 1) substantial contributions to conception and design, acquisition of data, or analysis and interpretation of data; 2) drafting the article or revising it critically for important intellectual content; and 3) final approval of the version to be published. Authors should meet conditions 1, 2, and 3.

Draft publications (manuscripts, abstracts, slides and posters) should be submitted to the RM-CTU for circulation to the relevant parties to allow sufficient time for review prior to submission. There will be a fifteen (15) day period to review abstracts or posters and a thirty (30) day period to review slides and manuscripts and respond to the author with any revisions.

The main trial results will be published in a peer-reviewed journal, on behalf of all collaborators. The manuscript will be prepared by a writing group, consisting of members of the TMG and selected participating clinicians. All participating clinicians will be acknowledged in the publication.

Any presentations and publications relating to the trial must be authorised by the TMG. Authorship of any secondary publications e.g. those relating to sub-studies, will reflect the intellectual and time input into these studies.

- No investigator may present or attempt to publish data relating to the POPS trial without prior permission from the TMG.

Trial etiquette

Before starting the trial, the protocol, patient information sheet and consent form must be approved by the RM/ICR joint Committee for Clinical Research. Once approved, the study may then be submitted to the relevant regulatory authorities.

It is the Chief and Principal Investigator's responsibility to update patients (or their authorised representatives, if applicable) whenever new information (in nature or severity) becomes available that might affect the patient's willingness to continue in the trial. The Chief and Principal Investigator must ensure this is documented in the patient's medical records and the patient is re-consented, where appropriate.

The Sponsor and Chief and Principal Investigator must ensure that the trial is carried out in accordance with the principles of GCP outlined in ISO14155, requirements of the Medical Devices Directives and their applicable UK regulatory requirements and The Declaration of Helsinki (http://www.wma.net/en/30publications/10policies/b3/).

Independent data monitoring committee (IDMC)

An independent Data Monitoring Committee will be set up to monitor the progress of the trial. They will meet at regular intervals as they see fit but at least annually. Following each meeting they will report their findings and recommendations to the chair of the Trial Management Group. All interim analyses will be supplied in confidence by the trial statistician to the IDMC together with any other analyses the IDMC request. The IDMC will advise on the frequency of reviews of the data on the basis of accrual and event rates.

The Independent Data Monitoring Committee will review a first interim report to assess recruitment and compliance to IGRT technique 6 months after the first randomisation or when a quarter of the required number of patients will be accrued which ever occurs first.

In a second interim analysis when the median follow-up will reach one year, the IDMC will look

at the first two years toxicity data. The final IGRT analysis will be conducted with recommendation from the IDMC when the minimum follow-up of the sub-study is two years. There will be early stopping rules for toxicity and tolerability of the ProSpare™ device, which will be approved by the independent data monitoring and trial steering committees.

10 Safety Reporting

10.1 Definitions

10.1.1 Adverse Device Event (ADE)

Adverse event related to the use of an investigational medical device.

- This includes any adverse event resulting from insufficiencies or inadequacies in the instructions for use, the deployment, the implantation, the installation, the operation, or any malfunction of the investigational medical device.
- This includes any event that is a result of a use error or intentional misuse.

10.1.2 Adverse Event (AE)

Any untoward medical occurrence, unintended disease or injury or any untoward clinical signs (including an abnormal laboratory finding) in subjects, users or other persons whether or not related to the investigational medical device.

- This includes events related to the investigational device or the comparator.
- This includes events related to the procedures involved (any procedure in the clinical investigation plan).

  For users or other persons this is restricted to events related to the investigational medical device.

10.1.3 Device Deficiency

Inadequacy of a medical device related to its identity, quality, durability, reliability, safety or performance, such as malfunction, misuse or use error and inadequate labelling.

10.1.4 Serious Adverse Device Effect (SADE)

Adverse device effect that has resulted in any of the consequences characteristic of a serious adverse event.

10.1.5 Serious Adverse Event (SAE)

Adverse event that

a) Led to a death,

b) Led to a serious deterioration in health that either:

- Resulted in a life-threatening illness or injury, or
- Resulted in a permanent impairment of a body structure or a body function, or
- Required in-patient hospitalization or prolongation of existing hospitalization, or
- Resulted in medical or surgical intervention to prevent life threatening illness or injury or permanent impairment to a body structure or a body function.

c) Led to foetal distress, foetal death or a congenital abnormality or birth defect.

This includes device deficiencies that might have led to a serious adverse event if

a) Suitable action had not been taken or

b) Intervention had not been made or

c) If circumstances had been less fortunate. These are handled under the SAE reporting system.

A planned hospitalization for pre-existing condition, or a procedure required by the Clinical Investigation Plan, without a serious deterioration in health, is not considered to be a serious adverse event.

10.1.6 Unanticipated Serious Adverse Device Effect (USADE)

Serious adverse device effect which by its nature, incidence, severity or outcome has not been identified in the current version of the risk analysis report. An anticipated effect is one which by its nature, incidence, severity or outcome has been previously identified in the risk analysis report

10.2 Operational definitions for (S)AEs

In the POPS trial, AE / ADE / SAE / SADE / USADE will be collected from the **start date of the patient's radiotherapy until 90 days from the end of treatment**. All events will be recorded in the medical notes and the appropriate section of the CRF and/or AE/ADE log. Events include all grade 4 acute or late bowel or bladder toxicity with the exception of urinary obstruction.

SAE / SADE / USADE will be reported to the sponsor as detailed in section 10.3.

10.2.1 Assessment of Severity

All adverse events will be graded for severity. The investigator will make an assessment of severity for each adverse event and this should be recorded in the CRF according to CTCAE v4.0.

NB: to avoid confusion or misunderstanding of the difference between the terms "serious" and "severe", the following note of clarification is provided: "Severe" is often used to describe

intensity of a specific event, which may be of relatively minor medical significance. "Seriousness" is the regulatory definition supplied in 10.1.5 above.

10.2.2 Assessment of Seriousness

The seriousness of an adverse event is assessed by the investigator using the criteria outlined in section 10.1.5.

10.2.3 Assessment of Causality

All adverse events will be evaluated by the Investigator for potential relationship to the medical device.

- **Unrelated:** where an event is not considered to be related to the device.
- **Possibly related:** the nature of the event, the underlying medical condition, concomitant medication or temporal relationship make it possible that the AE has a causal relationship to the device.

The investigator must endeavour to obtain sufficient information to determine the causality or the AE (i.e. device, other illness, progressive malignancy etc) and must provide his/her opinion of the causal relationship between each AE and the device. This may require instituting supplementary investigations of significant AEs based on their clinical judgment of the likely causative factors and/or include seeking a further opinion from a specialist in the field of the AE.

10.2.4 Assessment of Expectedness

If the AE is judged to be related to the device, the Investigator will make an assessment of expectedness based on knowledge of the reaction and any relevant product information as documented the risk analysis report. The event will be classed as either;

- **Expected:** the reaction is consistent with the effects of the device listed in the risk analysis report.
- **Unexpected:** the reaction is not consistent with the effects listed in the risk analysis report.

10.3 Reporting of Adverse Events

10.3.1 Reportable events

The following events are considered reportable:

- Any SAE
- Any any Investigational Medical Device Deficiency that might have led to a SAE if

a) Suitable action had not been taken or

b) Intervention had not been made or

c) If circumstances had been less fortunate

- New findings/updates in relation to already reported events

For each event, the following information will be collected:

- Full details in medical terms and case description
- Event duration (start and end dates, if applicable)
- Action taken
- Outcome
- Seriousness criteria
- causality (i.e. relatedness to device / investigation), in the opinion of the investigator
- Whether the event would be considered expected or unexpected.

All reportable events must be notified to the Chief Investigator **within 24 hours** of the site staff becoming aware of the event. The responsibility of notifying reportable events to the MHRA and REC has been delegated by the Sponsor to the Chief Investigator.

Any change of condition or other follow-up information should be faxed to the Chief Investigator at least **within 24 hours** of the information becoming available. Events will be followed up until the event has resolved or a final outcome has been reached.

The Chief Investigator will collaborate with the device manufacturer to notify the MHRA and REC of reportable events.

10.3.2 Reporting Timelines

10.3.2.1 All SAE (SADE / USADE) which indicates an imminent risk of death, serious injury, or serious illness and that requires prompt remedial action for other patients / subjects, users or other persons or a new finding to it must be reported to the MHRA and REC immediately, but not later than **2 calendar days** after awareness by Chief Investigator of a new reportable event or of new information in relation with an already reported event.

10.3.2.2 Any other reportable events or a new finding/update to a reportable event must be reported to the MHRA and REC immediately, but not later than **7 calendar days** following the date of awareness by the Chief Investigator of the new reportable event or of new information in relation with an already reported event.

10.3.4 Other Reporting Requirements

POPS is a multi-centre study; the Chief Investigator is responsible for informing Investigators at all participating sites of any reported USADEs and any other arising safety information.

11 Statistical Considerations

Sample size

Analyses of outcome data will be on the basis of intention to treat and therefore include all patients randomized including those who deviate from trial protocol. However, randomized patients who have not received at least one treatment dose will not be included in the toxicity or PRO analysis.

The sample size is based on the primary endpoint which is the change in patient reported EPIC bowel function and bother at baseline and at 2 years after prostate bed radiotherapy between the two groups.

There is no published literature detailing EPIC scores in the prostate bed radiotherapy setting. As the planned target volume is similar to prostate radiotherapy we have extrapolated the data from patient reported outcomes in patients who have received radical prostate radiotherapy using IMRT [26].

The primary endpoint of their study was the mean change in quality of life scores from pre treatment to post treatment in the acute (<90 days), late (12-24 months) time periods.

The clinically meaningful change in health related quality of life is defined as a mean change score exceeding half the standard deviation of the baseline value.

In the study, which included 153 patients, the mean EPIC bowel score at baseline was 94.3, with a standard deviation of 10.9. We therefore plan to use 5.45 as our minimum absolute difference between the two groups at two years to identify a clinically meaningful difference.

172 patients (86 patients per group) are required to detect a 5.45 point difference in the EPIC bowel score, with 90% power, 2-sided 5% alpha.

To allow for a non evaluable rate at 24 months of 30% due non-compliance with the 24 month questionnaire, it is assumed that 70% of patients will be evaluable for the primary endpoint. The target sample size is therefore 245 patients.

The IDMC will assess EPIC questionnaire compliance rates at regular intervals and make recommendations for changes in sample size to ensure 172 evaluable patients as appropriate.

The expected duration of recruitment would be 2 years, this is estimated in view of the RADICALS (ISRCTN 40814031: www.radicals-trial.org) trial recruitment and local hospital data.

The expected duration of follow-up after the end of accrual will be for 5 years, as there is evidence that late toxicity outcomes can continue to between 2 to 5 years. There is limited published long term patient reported outcomes data.

Randomization and stratification

Randomisation process will use minimization with a random element and with stratification factors listed below as balancing factors.

Patients will be stratified by:

- Clinical indication for prostate bed radiotherapy: adjuvant or salvage
- Treatment centre – as each centre will need to declare their institutional standard daily dose-fractionation and total dose prior to enrolment of the first patient (i.e. it will be the same for all patients), there is no need to stratify separately for dose.
- Hormone therapy
    - No androgen therapy
    - 6 months hormone therapy
    - >6 months but <24 months
    - ≥24months

Boost given to macroscopic disease (allowed dose up to 74Gy in 2 Gy per fraction) – centres will need to declare if they would propose to give a boost to macroscopic disease at time of their enrolment.

Treatment allocation will not be masked and clinicians will not be blinded.

Statistical Analysis

All analysis variables, both predictors and outcomes, will be examined carefully prior to any formal statistical analysis. Standard graphical methods, including histograms and boxplots, will be used to examine overall distributions and identify potential outliers, which will be confirmed prior to inclusion in analysis. Each measure will be summarized using standard descriptive statistics, including means and standard deviations, medians, and ranges, for continuous measures and proportions for categorical measures. Confidence intervals (95%) will be included. A full statistical analysis plan will be written and agreed before any analysis is undertaken.

Primary endpoint analysis

The primary endpoint will be compared between randomised groups using a two-sample t-test (or non-parametric Mann-Whitney test if scores are not normally distributed). The primary analysis will include all available data from all patients randomized within the trial. If a significant proportion of patients are not evaluable, the pattern of missing data will be investigated. If it appears data may not be missing at random i.e. there are potential differences in EPIC bowel outcome between evaluable and non-evaluable patients, a sensitivity analysis will be performed assuming all patients with missing 24 month EPIC bowel data have a total EPIC score equal to the lower 25% quartile of all 24 month total scores received.

Analysis of covariance will be used to investigate other patient, treatment and clinical factors which may be associated with change in EPIC bowel score from baseline to 24 months post-treatment. Longitudinal modeling techniques will be used to investigate change in EPIC bowel score over time.

Secondary endpoints

A similar approach to that taken for the primary endpoint will be used to analyse other secondary patient-reported acute and late toxicity and quality of life endpoints (EPIC, IPSS, IIEF-5, IBQD, Vaizey and SF-12 scores) . To make some adjustment for multiple testing, statistical tests on secondary endpoints relating to comparisons based on PROs will be performed at the 1% significance level. In addition proportions and confidence intervals will be reported by timepoint using pre-defined scoring categories (categories will be defined using published definitions for individual scoring tools).

Clinician assessed acute and late toxicity data (RTOG, Gulliford, CTCAE grades) will be analysed by comparing the proportion of patients experiencing a particular toxicity (grade 1 or above) at a time point by randomised treatment group using Chi-squared (or Fisher's exact test as appropriate). The proportions experiencing severe toxicity (grade 2 or worse) will also be compared.

Time to event endpoints will be displayed graphically using Kaplan-Meier plots, with randomized groups compared using a stratified log rank test adjusting for stratification variables (excluding center). Hazard ratio from a Cox proportional hazards models that includes the stratification factors will be reported with 95% confidence interval. Methods to account for non-proportionality will be used if required.

Dosimetric assessments will be summarized by arm using descriptive statistics.

Tolerability will be reported using patient-completed questionnaires as a percentage (with 95% confidence intervals).

Exploratory analysis will be performed to investigate the association of clinician-reported toxicity grades with patient-reported toxicity and quality of life scores. Exact methods used will be fully documented in the SAP prior to analysis, and are likely to include graphic methods such as histograms and box-and-whisker plots to summarize distribution of scores in patients grouped by maximum toxicity grade (0-1 vs 2+), use of nonparametric correlation coefficients to quantify the association between grades and scores, and calculation of area under the ROC curve to assess agreement of patient-reported scores with presence of grade 2+ toxicity.

Timing of analysis

Analysis of trial data will be done in three phases. Each endpoint will be analysed and reported only once (with the exception of biochemical PFS). The first analysis will occur once all patients have completed their 18 week post-radiotherapy assessment, and will include all acute toxicity endpoints, tolerability, patient reported outcomes to 18 weeks only, and dosimetric evaluation. The second analysis will occur once all patients have completed their 24 month post-

radiotherapy assessment, and will include all late toxicity and patients reported outcomes (including sexual function) to 24 months only, and biochemical progression-free survival. The third and final analysis will occur once all patients have reached 60 months post radiotherapy, and will include EPIC scores at 60 months, and updated biochemical PFS.

Data monitoring

⇒ Interim analyses will be undertaken at least annually, more frequently if problems are apparent due to slowness of accrual or toxicity. Interim analyses will be reviewed confidentially and reported upon by an Independent Data Monitoring Committee (IDMC).

⇒ The IDMC will review accrual, safety and efficacy data. The replacement of patients who withdraw from the study or who are not evaluable for reasons deemed not to be treatment related will be based on the advice of this committee.

⇒ As the proposed duration of recruitment is 24 months, it is unlikely that sufficient data on the efficacy i.e. biochemical progression free survival to inform the early stopping rule of the trial for efficacy. Therefore, no formal stopping rule based on the biochemical progression free survival is proposed.

  ⇒ Published data has shown no significant change in biochemical progression free survival in patients treated with image-guided radiotherapy and reduced margins in prostate radiotherapy, therefore, we do not feel that there should be an increase in this trial population.

⇒ There will be early stopping rules for acute toxicity (defined at the time of week 18 post-radiotherapy) and tolerability of the ProSpare™ device throughout the total duration of radiotherapy in those randomized patients to that arm. These will be finalized in liaison with the IDMC.

  ⇒ Acute toxicity: in a study of 176 patients treated with IMRT after radical prostatectomy in the salvage setting, the overall rate of acute grade >2 GI toxicity was 9.8% [27]. The proportion of patients who have no acute grade2+ GI toxicity free should be ≥90%. If this proportion falls to ≤75% it would be desirable to stop the trial with the conclusion that GI toxicity rates are too high. If 42 or fewer of the first 50 patients are GI toxicity free (i.e. 8 or more have GI toxicity G2+) at 18 weeks, consideration will be given to stopping the trial. Given these criteria, there is an 88% chance the trial will not be

stopped if the grade2+ GI toxicity free rate is 90% but only a 5% chance it will not be stopped if the true GI toxicity free rate is 70%.

⇒ Tolerability of ProSpare™ device: if <85% of patients tolerate the device for <85% of all radiotherapy treatments. This has been calculated to take into account the total number of fractions missed due to poor tolerability, which would then cause an end of treatment delay greater than a week.

An independent Data Monitoring Committee will be set up to monitor the progress of the trial. They will meet at regular intervals as they see fit but at least annually. Following each meeting they will report their findings and recommendations to the chair of the Trial Management Group. All interim analyses will be supplied in confidence by the trial statistician to the IDMC together with any other analyses the IDMC request. The IDMC will advise on the frequency of reviews of the data on the basis of accrual and event rates.

The Independent Data Monitoring Committee will review a first interim report to assess recruitment and compliance to IGRT technique 6 months after the first randomisation or when a quarter of the required number of patients will be accrued which ever occurs first.

In a second interim analysis when the median follow-up will reach one year, the IDMC will look at the first two years toxicity data. The final IGRT analysis will be conducted with recommendation from the IDMC when the minimum follow-up of the sub-study is two years. There will be early stopping rules for toxicity and tolerability of the ProSpare device, which will be approved by the independent data monitoring and trial steering committees.

12 Quality of life assessment

Rationale

- Treatments for prostate cancer are associated with specific side effects resulting in considerable impairment in several health-related quality of life domains, therefore it is critical for the inclusion of these assessments for an understanding of which approach is best from the patient's perspective.

- This trial is comparing two techniques, and therefore it is important to know about the potential issues impacting on patient's quality of life.
- Physician documented symptom assessment is valuable, however, the correlation between these and patient reported outcomes are poor. Therefore, it is important for quality of life to be evaluated regularly through treatment and to have regular assessments and long term follow up.
- In our institution, we are familiar with the available quality of life tools and these have been part of large prostate cancer randomized controlled trials.
- These trials have included the use of FACT-P, UCLA/RAND Prostate Cancer Index, EPIC, SF-12, modified IBDQ, Vaizey and IPSS, IIEF-5 questionnaires.

QoL instruments

This trial is trying to determine if a new radiotherapy technique improves patient reported outcomes, particularly in relation to bowel symptoms. Therefore, it is prudent to encompass a number of validated patient reported questionnaires.
EPIC and SF-12 questionnaires have been used as part of the large, multicenter randomized trial and it seemed appropriate and clinically relevant to use them for this study. The EPIC bowel assessment questions do not ask in detail about faecal incontinence and this is the gastrointestinal symptom that causes greatest distress to patients after radiotherapy. Analysis of the dosimetric data from the POPS patients, will include evaluation of the spatial distribution of radiation to the anal canal and rectum. Therefore, the Vaizey incontinence questionnaire, which is brief, taking around 2-3 minutes to complete will enable us to correlate these evaluations with specific bowel incontinence information reported directly by patients.

EPIC

EPIC was developed by researchers at University of Michigan and UCLA to measure health related quality of life among men with prostate cancer. It represents an adaptation of the UCLA Prostate Cancer Index, modified to enhance sensitivity to therapy effects by increasing the number of prostate-targeted items to 50, compared to 20 in the original UCLA-PCI. EPIC has been validated in men with localized prostate cancer who underwent surgery, external beam

radiotherapy or brachytherapy with or without hormone adjuvants. EPIC is sensitive to specific HRQOL effects of these therapies and to HRQOL effects of cancer progression. EPIC assesses the disease-specific aspects of prostate cancer and its therapies and comprises four summary domains: Urinary, Bowel, Sexual and Hormonal. Response options for each EPIC item form a Likert scale, and multi-item scale scores are transformed linearly to a 0 -100 scale with higher scores representing better health-related quality of life. The function and bother questions will be scored together for comparison between groups.

In addition, the overall urinary, bowel, and sexual bother questions, which are scored on a 5 point scale (no problems, very small problem, small problem, moderate problem or big problem) will be grouped into 3 clinically relevant categories (no problem, very-small to small problem, and moderate to big problem) and analysed between the two groups.

Modified IBDQ

The modified IBDQ consists of 32 questions each graded from 1 (worst possible symptom) to 7 (symptom absent or not changed since before radiotherapy). A total score will be calculated by summing together all the individual scores; the highest attainable score of 224 indicates the patient is asymptomatic and the lowest attainable score of 32 indicates severe symptoms. Only patients with answers for all 32 questions will be included in analysis of total score at that time point (Guyatt et al, 1989, Olopade et al, 2005). The distribution of the total score will be displayed as a histogram. Total score will be summarised by the median, IQR and range by treatment group at each time point. The time point and symptom score of primary interest is week 18 from the start of radiotherapy bowel scores. Data will be presented as the proportion of patients at week 18 with no change in bowel symptoms (score 7 = not at all/normal) within each treatment group. If there is at least one bowel symptom question answered then the worst score will be calculated. Data will also be categorised in to a four point scale of 'none' (not at all/normal); 'mild' (slight or some increase in frequency); 'moderate' (moderate increase in frequency); 'severe' (very/frequently and more than ever before frequency). The study has not been designed to formally compare the two treatment groups, however, should potentially clinically significant differences between treatment groups be apparent at 2 years then a statistical comparison will be made. The results of any such comparison will be interpreted with caution and under the assumption that it is hypothesis generating and not powered to detect differences. A 1% significance level will be used. One-sided p-values will be presented. Systemic,

emotional and social symptoms will also be summarised in this way at 2 years and all other time points but no statistical comparisons will be made. Data will also be displayed graphically as barcharts.

The **modified IBDQ** individual questions can be grouped and summarised as symptom scores as follows:

- Bowel symptoms
    - Had your bowel open (Q1); loose bowel movements (Q5); been troubled by pain in bottom (Q9); troubled by cramps in tummy or bottom (Q13); passed large amount of gas (Q17); troubled by bloating (Q20); problem with bleeding from bottom (Q22); bowel open but nothing happening (Q24); troubled by accidental soiling (Q26); felt disgusted by bowel habits (Q29).
- Systemic symptoms
    - Felt tired or worn out (Q2); worried about energy levels (Q6); generally unwell (Q10); waking at night or difficult sleeping (Q14); worried about getting to weight you would like (Q18).
- Emotional symptoms
    - Felt frustrated, impatient or restless (Q3); worried about having to have something done about bowels (Q7); worried about not being able to find a laboratory (Q11); depressed or discouraged (Q15); worried about illness (Q19); relaxed and free from tension (Q21); embarrassed about bowels (Q23); felt fearful or upset (Q25); felt angry due to bowel problems (Q27); felt irritable (Q30); experienced lack of understanding from others (Q31); felt satisfied, happy or pleased with life (Q32).
- Social symptoms
    - Unable to do what you want because of bowels (Q4); had to cancel engagement because of bowels (Q8); prevented from doing leisure or sports by bowels (Q12); not gone somewhere because there is no lavatory nearby (Q16); felt limited in sexual activity because of bowels (Q28).

IPSS

The IPSS questionnaire consists of 7 questions each graded from 0 (no problems) to 5 (always a problem) with total score summarised as: mildly symptomatic 0-7; moderately symptomatic 8-19; and severely symptomatic 20-35. At each time point the total score will be summarised by the median, IQR, range and proportion defined as having symptoms which are mild, moderate or severe, by treatment group. Only patients with complete IPSS questionnaire will be included in the total score analysis at each time point. The overall quality of life due to urinary symptoms (graded from 0 to 6) will be presented as number and frequency of each grade at each time point by treatment group. Data will also be presented as frequency and percentage for each question at each time point by treatment group. Data will also be displayed graphically as stacked barcharts. Although the study is not powered for formal statistical comparisons, if clinically significant differences are evident then a statistical comparison may be made at the 2 year time point using a one-sided 1% significance level.

Vaizey questionnaire

The Vaizey questionnaire consists of 7 questions and will be summarised as a total score ranging from 0 (no problems with bowel continence) to 24 (very severe problems with incontinence); see table 1. At each assessment time point the total Vaizey score will be presented as a median, IQR and range by treatment group. Only patients with complete Vaizey questionnaires will be included in the total score analysis at each time point. The proportion of patients defined as 'sometimes', 'weekly or 'daily' will be summarised as frequency and percentage for each individual question at each time point and by treatment group. Data will also be displayed graphically as stacked barcharts. Although the study is not powered for formal statistical comparisons, if clinically significant differences are evident then a statistical comparison may be made at the 18 week time point using a one-sided 1% significance level.

SF-12

Inter-scale correlation between EPIC and Medical Outcomes Study 12-item Short Form Health Survey (SF-12) have indicated that efficient yet comprehensive health related quality of life assessment can be achieved by co-administering EPIC with SF-12. SF-12 produces two summary scales assessing physical function and mental well-being.

IIEF-5

This involves 5 questions assessing patient's perception of erectile function.

Statistical considerations

- This has been considered in the main part of the protocol and a statistical analysis plan will be formulated prior to quality of life analysis.

The specific QOL scoring manuals advise on whether a questionnaire can be counted or not according to how much data is missing. For example:

- **EPIC**: If ≥20% of the items that comprise a domain summary score or subscale score are missing a response, the corresponding domain summary or subscale score cannot be calculated (EPIC).
- **SF12**: If any missing or out-of-range values are found for those items, the PCS and MCS scores cannot be calculated.

13 Radiotherapy Quality Assurance (QA)

The level of trial-specific QA will depend on the IMRT experience of each centre and whether they have completed the QA for another IMRT trial. All centres that have participated in the CHHiP trial and as such have undergone QA for a prostate radiotherapy trial. A number of centres (RMH, Clatterbridge, Addenbrookes, Ipswich) have also completed the CHHiP or PARSPORT trials IMRT QA process. Working with the National Radiotherapy Trials QA group and with the support of the NCRI CTRRWG WS4, each centre will be assessed against the 9-step IMRT credentialing program as proposed below.

Nine step IMRT credentialing program

Current IMRT QA is based on a 9 step programme initially developed by the Radiotherapy Trial Quality Assurance team. These 9 steps are now providing the basis of an IMRT credentialing programme. The 9 steps are listed below:

1. Outlining exercise

2. Planning exercise

3. Process document

4. Baseline questionnaire and trial specific questionnaire

5. Verification of electronic transfer of data

6. TPS verification

7. Individual field measurement

8. Combined field measurement

9. Dose point measurement

Pre-trial quality assurance programme

The following will need to be completed by participating centres prior to site activation.

1. Facility questionnaire

2. Process document

3. Benchmark outlining case

4. Benchmark planning case

5. IGRT competency programme - on-line training package, practical workshop and independent competency check

14 Ethical considerations

It is the responsibility of the Chief Investigator to obtain a favourable ethical opinion (main REC approval). It is the responsibility of the Principal Investigator at each participating centre to obtain site-specific approval of the trial protocol and any subsequent amendments. All correspondence with the local REC should be filed by the Principal Investigator in the Site Investigator File.

It is the responsibility of the Principal Investigator to give each patient, prior to inclusion in the trial, full and adequate verbal and written information regarding the objective and procedures of the trial and the possible risks involved. Sufficient time (a minimum of 24 hours) should be allowed for the patient to decide on trial entry. Patients must be informed about their right to withdraw from the trial at any time. Written patient information must be given to each patient

before enrolment. The written patient information is an approved patient information sheet according to national guidelines.

It is the responsibility of the Principal Investigator or designated representative, to obtain signed informed consent from all patients prior to inclusion in the trial.

Patient Information

The importance of providing a high level of information to patients is recognised. Local leaflets on radiotherapy should be provided by each centre, but these must be approved by the appropriate local research committees before distribution.

Data Sharing

Data arising from this research will be managed and made available to maximise public benefit. Data sharing will be in a timely and responsible manner. Appropriate regulatory permissions relating to the ethical use of data must be in place before the data can be shared. Requests to use trial data must be submitted in writing to the RM-CTU for approval by the TMG and TSC.

Data Protection Act 1998

RM-CTU will comply with all aspects of the DPA 1998. Any requests from patients for access to data about them held at RM-CTU should be directed to the Trial Manager in the first instance who will refer the request to the Data Protection Officer at the Royal Marsden NHS Foundation Trust.

15 Administrative responsibilities

Responsibilities of participating centres

Centres wishing to recruit to this study will be asked to provide evidence that they can deliver protocol treatment.

This will include:

- Putting and keeping in place arrangements to adhere to the principles of GCP.
- Keeping a copy of all “essential documents” (as defined under the principles of GCP) and ensuring appropriate archiving of documentation once the trial has ended.
- Providing evidence that local practice conforms to the standards set in the NICE

Improving Outcomes Guidance.

- Providing evidence that radiotherapy quality assurance is available.
- Treatment centres are not allowed to deviate from

Responsibilities are defined in an agreement between an individual participating centre and The Royal Marsden NHS Foundation Trust.

Trial Insurance

The Institute of Cancer Research has Clinical Trials Insurance, currently with Arthur J. Gallagher International. Standard NHS indemnity policy applies for clinical care and negligence indemnity.

Inclusion of private patients will be subject to the site ensuring appropriate insurance and indemnity arrangements are in place.

16 References:

1. CRUK Prostate Cancer, Cancer Statistics Key Stats. November 2014 [http://publications.cancerresearchuk.org/downloads/product/CS_KF_PROSTATE.pdf]
2. Prostate Cancer Advisory Group. Advice on the Development of Robotic Assisted Radical Prostatectomy in England. updated 2012. http://www.baus.org.uk/_userfiles/pages/files/Publications/PCAGRoboticProstatectomyinEngland.pdf
3. Valicenti, R.K., et al., *Adjuvant and salvage radiation therapy after prostatectomy: American Society for Radiation Oncology/American Urological Association guidelines.* Int J Radiat Oncol Biol Phys, 2013. **86**(5): p. 822-8.
4. Thompson, I.M., et al., *Adjuvant radiotherapy for pathological T3N0M0 prostate cancer significantly reduces risk of metastases and improves survival: long-term followup of a randomized clinical trial.* J Urol, 2009. **181**(3): p. 956-62.
5. Bolla, M., et al., *Postoperative radiotherapy after radical prostatectomy for high-risk prostate cancer: long-term results of a randomised controlled trial (EORTC trial 22911).* Lancet, 2012. **380**(9858): p. 2018-27.
6. Wiegel, T., et al., *Phase III postoperative adjuvant radiotherapy after radical prostatectomy compared with radical prostatectomy alone in pT3 prostate cancer with postoperative undetectable prostate-specific antigen: ARO 96-02/AUO AP 09/95.* J Clin Oncol, 2009. **27**(18): p. 2924-30.
7. Ashman, J.B., et al., *Whole pelvic radiotherapy for prostate cancer using 3D conformal and intensity-modulated radiotherapy.* Int J Radiat Oncol Biol Phys, 2005. **63**(3): p. 765-71.
8. Koontz, B.F., et al., *Dosimetric and radiobiologic comparison of 3D conformal versus intensity modulated planning techniques for prostate bed radiotherapy.* Med Dosim, 2009. **34**(3): p. 256-60.
9. Malone, S., et al., *Postoperative radiotherapy for prostate cancer: a comparison of four consensus guidelines and dosimetric evaluation of 3D-CRT versus tomotherapy IMRT.* Int J Radiat Oncol Biol Phys, 2012. **84**(3): p. 725-32.
10. Huang, K., et al., *Inter- and intrafraction uncertainty in prostate bed image-guided radiotherapy.* Int J Radiat Oncol Biol Phys, 2012. **84**(2): p. 402-7.
11. Canter, D., et al., *Implantation of electromagnetic transponders following radical prostatectomy for delivery of IMRT.* Can J Urol, 2010. **17**(5): p. 5365-9.
12. Langenhuijsen, J.F., et al., *Postprostatectomy ultrasound-guided transrectal implantation of gold markers for external beam radiotherapy. Technique and complications rate.* Strahlenther Onkol, 2013. **189**(6): p. 476-81.
13. Fiorino, C., et al., *Rectal and bladder motion during conformal radiotherapy after radical prostatectomy.* Radiother Oncol, 2005. **74**(2): 187-95
14. Klayton, T., et al., *Prostate Bed Motion during Intensity-Modulated Radiotherapy Treatment.* Int J Radiat Oncol Biol Phys, 2012.
15. Croke, J., et al., *Proposal of a post-prostatectomy clinical target volume based on pre-operative MRI: volumetric and dosimetric comparison to the RTOG guidelines. Radiat Oncol*. 2014;**9**(1):303.
16. de Leon, J.F., et al., *Superior target volume and organ stability with the use of endorectal balloons in post-prostatectomy radiotherapy.* J Med Imaging Radiat Oncol, 2015.

17. Smeenk, R.J., et al., *Endorectal balloon reduces anorectal doses in post-prostatectomy intensity-modulated radiotherapy.* Radiother Oncol, 2011.
18. Institute of Medicine. Initial National Priorities for Comparative Effectiveness Research: Report Brief. (http://www.iom.edu/~/media/Files/Report%20Files/2009/ComparativeEffectivenessResearchPriorities/CER%20report%20brief%2008-13-09.ashx) June 2009
19. Food and Drug Administration. Guidance for Industry Patient-Reported Outcome Measures: Use in Medical Product Development to Support Labeling Claims. Draft Guidance. February, 2009.
20. AHRQ Effective Health Care Program. Clinical and Comparative Effectiveness Research Methods Symposium. AHRQ Conference Center. June 1 - 2, 2009.
21. Sonn G.A. et al., *Differing perceptions of quality of life in patents with prostate cancer and their doctors.* J Urol 2009. 182(5): 2296-2303
22. Litwin M.S. et al., *Differences in urologist and patient assessments of health related quality of life in men with prostate cancer: results of the CAPSURE database.* J Urol 1998. 159(6) 1988-1992
23. Wei J.T., et al. *Development and validation of the expanded prostate cancer index composite (EPIC) for comprehensive assessment of health-related quality of life in men with prostate cancer.* Urology 2000. 56; 899-905
24. Martin N.E. et al., *Defining a standard set of patient-centred outcomes for men with localised prostate cancer.* Eur Urol 2015 67(3) 460-467
25. Michalski, J.M., et al., *Development of RTOG consensus guidelines for the definition of the clinical target volume for postoperative conformal radiation therapy for prostate cancer.* Int J Radiat Oncol Biol Phys, 2010. **76**(2): p. 361-8.
26. Gray P.J. et al., *Patient-Reported Outcomes After 3-Dimensional Conformal, Intensity-Modulated, or Proton Beam Radiotherapy for Localised Prostate Cancer.* Cancer 2013. 119:1729-35
27. Goenka, A., et al., *Improved toxicity profile following high-dose postprostatectomy salvage radiation therapy with intensity-modulated radiation therapy.* Eur Urol. 2011;60(6): 1142–1148.

17 Signature pages:

The Institute of Cancer Research

The Royal Marsden NHS Foundation Trust

CHIEF INVESTIGATOR SIGNATURE PAGE

As Chief Investigator I confirm that this protocol reference: CCR 4359 entitled 'A randomised phase II trial assessing **P**ost-**O**perative use of **P**ro**S**pare™, a rectal obdurator in prostate cancer radiotherapy' will be conducted in compliance with the requirements outlined in BS EN ISO 14155:2011 'Clinical investigation of medical devices for human subjects – Good clinical practice (ISO 14155:2011)' and all other relevant clinical trial regulations including the Sponsor's Standard Operating Procedures (SOPs).

Further, I confirm that the protocol has been designed in accordance with EMEA ICH Topic E9 'Statistical Principles for Clinical Trials' and that the findings of the study will be made publically available without unnecessary delay and will constitute an honest, accurate and transparent account of the study with any discrepancies from the agreed protocol explained.

Study Title: A randomised phase II trial assessing **P**ost-**O**perative use of **P**ro**S**pare™, a rectal obdurator in prostate cancer radiotherapy ('POPS')

Sponsor Protocol Number: CCR 4359

Name and Title of Chief Investigator: Professor David Dearnaley

Signed: ______________________

Date: 16/03/16

CCR 4359 Signatory Page 1 of 2

Principal Investigator Signature Page

Study Title: A randomised phase II trial assessing **P**ost-**O**perative use of **P**ro**S**pare^{TM}, a rectal obdurator in prostate cancer radiotherapy ('POPS')

Sponsor protocol number: CCR 4359

I have received and reviewed the current protocol reference: CCR 4359 entitled 'A randomised phase II trial assessing **P**ost-**O**perative use of **P**ro**S**pare^{TM}, a rectal obdurator in prostate cancer radiotherapy' and am in agreement with my responsibilities as an Investigator under the UK Clinical Trials Regulations[1], the guidelines of Good Clinical Practice (GCP) the Declaration of Helsinki and the applicable regulations of the relevant NHS Trusts and the trial protocol. I therefore agree to conduct the trial in accordance with the protocol and accompanying regulations and guidelines. This will include directing and assisting the staff under my control who will be involved in the trial and ensuring that all staff members are aware of their clinical trial responsibilities.

Site Address: THE ROYAL MARSDEN NHS FOUNDATION TRUST
DOWNS ROAD, SUTTON. SURREY SM2 5PT

Name of Investigator: DR. ALISON TREE

Title: DR

Signed: [signature]

Date: 16.3.16

1The Medicines for Human Use (Clinical Trials) Regulations (S.I. 2004/1031) and any subsequent amendments to it.

CCR 4359 Signatory Page 2 of 2
