# Supplementary material for: Preliminary evaluation of ProSpare®, a rectal obturator for prostate bed radiotherapy within the randomised multicentre POPS trial (NCT02978014)
Source: Front Oncol. 2026 Oct 12;16:1758462. doi: 10.3389/fonc.2026.1758462 (PMC13696223; doi:10.3389/fonc.2026.1758462)
Supplement: Supplementary file 2 [file DataSheet2.docx]

# POPS trial appendix (1)

Table of Contents

[POPS trial appendix (1)	1](#_Toc215450512)

[Figure 1a – Sagittal images of CTV (green contour), with trial PTV margins (red contour) with and without the ProSpare® device.	2](#_Toc215450513)

[Figure 1b - In situ positioning on cone beam CT.	2](#_Toc215450514)

[Table 1- Reason for withdrawal from trial	3](#_Toc215450515)

[Table 2 – Adherence to randomised allocation and planned radiotherapy treatment	3](#_Toc215450516)

[Table 3 – Proposed and received radiotherapy dose and fractionation	4](#_Toc215450517)

[Table 4 – Stratification variables	4](#_Toc215450518)

[Table 5 - Medications recorded for hypertension, diabetes, hypercholesterolaemia and anti-coagulation	5](#_Toc215450519)

[Table 6 – CTCAE Genitourinary (GU) toxicity at 18 weeks	6](#_Toc215450520)

[Table 7 – CTCAE Gastrointestinal (GI) toxicity at 18 weeks	8](#_Toc215450521)

[Table 8 – CTCAE sexual dysfunction at 18 weeks	9](#_Toc215450522)

[Table 9 – EPIC bowel score at pre-radiotherapy and 18 weeks	9](#_Toc215450523)

[Table 10 – IPSS symptom scores pre-radiotherapy and at 18 weeks	10](#_Toc215450524)

[Table 11 – IPSS QoL scores pre-radiotherapy and at 18 weeks	11](#_Toc215450525)

[Table 12 – IIEF-5 score (continuous) pre-radiotherapy and at 18 weeks	11](#_Toc215450526)

[Table 13 – IIEF-5 score (categorical) pre-radiotherapy and at 18 weeks	12](#_Toc215450527)

[Table 14 – IBDQ score pre-radiotherapy and at 18 weeks	12](#_Toc215450528)

[Table 15 – Vaizey score pre-radiotherapy and at 18 weeks	13](#_Toc215450529)

[Table 16 – SF-12 scores pre-radiotherapy and at 18 weeks	13](#_Toc215450530)

[Table 17 – Patient reported ProSpare tolerability before treatment	14](#_Toc215450531)

[Table 18 – Patient reported ProSpare tolerability after treatment	15](#_Toc215450532)

[Table 19 – Rectal dose characteristics with and without ProSPare® use	16](#_Toc215450533)

[Table 20 – Anal Canal dose characteristics with and without ProSPare® use	16](#_Toc215450534)

## Figure 1a – Sagittal images of CTV (green contour), with trial PTV margins (red contour) with and without the ProSpare® device.

## Figure 1b - In situ positioning on cone beam CT.

## Table 1- Reason for withdrawal from trial

| Arm | Timing of withdrawal | Reason |
| --- | --- | --- |
| Prospare | Before radiotherapy | Increasing bowel symptoms before radiotherapy |
| Prospare | 22 month post radiotherapy | Terminal prognosis |
| Standard of care | Before radiotherapy | None given |
| Standard of care | 12 months post radiotherapy | None given |

## Table 2 – Adherence to randomised allocation and planned radiotherapy treatment

|  | Randomisation arm | | | | | |
| --- | --- | --- | --- | --- | --- | --- |
|  | Prospare (N=77) | | Standard of Care (N=81) | | Total | |
|  | N | % | N | % | N | % |
| Received allocated treatment |  |  |  |  |  |  |
| No | 1 | 1.3 | 1 | 1.2 | 2 | 1.3 |
| Yes | 76 | 98.7 | 80 | 98.8 | 156 | 98.7 |
|  |  |  |  |  |  |  |
| Radiotherapy treatment |  |  |  |  |  |  |
| As planned | 67 | 87.0 | 65 | 80.2 | 132 | 83.5 |
| Changed from plan | 10 | 13.0 | 16 | 19.8 | 26 | 16.5 |
|  |  |  |  |  |  |  |
| Patient adherence to planned RT (fractions) | | | | | | |
| Complete adherence | 76 | 98.7 | 79 | 97.5 | 155 | 98.1 |
| Less than complete adherence | 1 | 1.3 | 2 | 2.5 | 3 | 1.9 |
|  |  |  |  |  |  |  |
| Patient adherence to planned RT (dose) | | | | | | |
| Complete adherence | 69 | 89.6 | 67 | 82.7 | 136 | 86.1 |
| Dose administered less than planned | 5 | 6.5 | 10 | 12.3 | 15 | 9.5 |
| Dose administered greater t44han planned | 3 | 3.9 | 4 | 4.9 | 7 | 4.4 |
|  |  |  |  |  |  |  |
| Patient adherence to planned RT (boost) | | | | | | |
| Adherent (with boost) | 1 | 1.3 | 0 | 0.0 | 1 | 0.6 |
| Adherent (without boost) | 75 | 97.4 | 79 | 97.5 | 154 | 97.5 |
| Boost not given as planned | 0 | 0.0 | 2 | 2.5 | 2 | 1.3 |
| Unplanned boost given | 1 | 1.3 | 0 | 0.0 | 1 | 0.6 |
|  |  |  |  |  |  |  |
| Number of missed fractions |  |  |  |  |  |  |
| 1 | 1 | 100.0 | 1 | 50.0 | 2 | 66.7 |
| 3 | 0 | 0.0 | 1 | 50.0 | 1 | 33.3 |

## Table 3 – Proposed and received radiotherapy dose and fractionation

PS = ProSpare®, SOC = Standard of Care

| Number of planned fractions | Number of fractions administered | | | | | | | | | | |  | |  |
| --- | --- | --- | --- | --- | --- | --- | --- | --- | --- | --- | --- | --- | --- | --- |
|  | 20 | | 30 | | | 32 | | | 33 | | | Total | | |
|  | PS | SOC | PS | SOC | | PS | SOC | | PS | SOC | | PS | | SOC |
| 20 | 44 | 40 | 0 | | 0 | 0 | | 0 | 0 | | 0 | 44 | 40 | |
| 33 | 0 | 0 | 0 | | 1 | 1 | | 1 | 32 | | 39 | 33 | 41 | |
| Total | 44 | 40 | 0 | | 1 | 1 | | 1 | 32 | | 39 | 77 | 81 | |

## Table 4 – Stratification variables

|  | Randomisation group for patients receiving radiotherapy | | | | | |
| --- | --- | --- | --- | --- | --- | --- |
|  | Prospare (N=77) | | Standard of Care (N=81) | | Total | |
|  | N | % | N | % | N | % |
| Radiotherapy indication |  |  |  |  |  |  |
| Adjuvant | 4 | 5.2 | 3 | 3.7 | 7 | 4.4 |
| Salvage | 73 | 94.8 | 78 | 96.3 | 151 | 95.6 |
|  |  |  |  |  |  |  |
| Radiotherapy boost planned |  |  |  |  |  |  |
| Yes | 1 | 1.3 | 2 | 2.5 | 3 | 1.9 |
| No | 76 | 98.7 | 79 | 97.5 | 155 | 98.1 |
|  |  |  |  |  |  |  |
| Planned duration of hormones |  |  |  |  |  |  |
| No Androgen Therapy | 10 | 13.0 | 11 | 13.6 | 21 | 13.3 |
| 6 months | 45 | 58.4 | 45 | 55.6 | 90 | 57.0 |
| > 6 months and < 24 months | 9 | 11.7 | 12 | 14.8 | 21 | 13.3 |
| > or = 24 months | 13 | 16.9 | 13 | 16.0 | 26 | 16.5 |
|  |  |  |  |  |  |  |
| Planned RT of lymph nodes |  |  |  |  |  |  |
| Yes | 12 | 15.6 | 16 | 19.8 | 28 | 17.7 |
| No | 65 | 84.4 | 65 | 80.2 | 130 | 82.3 |

## Table 5 - Medications recorded for hypertension, diabetes, hypercholesterolaemia and anti-coagulation

|  | Randomisation group for patients receiving radiotherapy | | | | | |
| --- | --- | --- | --- | --- | --- | --- |
|  | Prospare (N=77) | | Standard of Care (N=81) | | Total | |
|  | N | % | N | % | N | % |
| Hypertension medication | 23 | 29.8 | 24 | 29.6 | 47 | 29.7 |
| ACE inhibitors (e.g. Ramipril) | 5 | 21.7 | 4 | 16.7 | 9 | 19.1 |
| Angiotensin II receptor antagonists (e.g. Candesartin) | 3 | 13 | 2 | 8.3 | 5 | 10.6 |
| Beta-Blockers (e.g. Propranalol, Atenolol) | 1 | 4.3 | 2 | 8.3 | 3 | 6.4 |
| Ca Channel blockers (e.g. Amlodipine) | 7 | 30.4 | 10 | 41.7 | 17 | 36.2 |
| Diuretic (e.g. thiazide) | 1 | 1.3 | 1 | 4.2 | 1 | 2.1 |
| Other | 6 | 26.1 | 5 | 20.8 | 11 | 23.1 |
| Not known | 1 | 4.3 | 0 | 0.0 | 1 | 2.1 |
|  |  |  |  |  |  |  |
| Diabetes medication | 5 | 6.5 | 4 | 4.9 | 9 | 5.7 |
| None (diet controlled) | 1 | 20 | 1 | 25 | 2 | 22.2 |
| Biguanides (Metformin) | 1 | 20 | 1 | 25 | 2 | 22.2 |
| Other | 3 | 60 | 2 | 50 | 5 | 55.6 |
|  | 13 | 16.9 | 13 | 16.0 | 26 | 16.5 |
|  |  |  |  |  |  |  |
| Anticoagulation medication | 11 | 14.3 | 8 | 9.9 | 19 | 12.0 |
| Aspirin | 3 | 27.3 | 3 | 37.5 | 6 | 31.6 |
| Other | 8 | 72.7 | 5 | 62.5 | 13 | 68.4 |
|  |  |  |  |  |  |  |
| Statin medication | 19 | 24.7 | 26 | 32.1 | 45 | 28.5 |
| Atorvastatin | 14 | 73.7 | 19 | 73.1 | 33 | 73.3 |
| Pravastatin | 1 | 5.3 | 1 | 3.8 | 2 | 4.4 |
| Simvastatin | 2 | 10.5 | 5 | 19.2 | 7 | 15.6 |
| Other | 1 | 5.3 | 1 | 3.8 | 2 | 4.4 |
| Not known | 1 | 5.3 | 0 | 0.0 | 1 | 2.2 |

## Table 6 – CTCAE Genitourinary (GU) toxicity at 18 weeks

|  | Randomisation group (patients who received radiotherapy only) | | | | | |
| --- | --- | --- | --- | --- | --- | --- |
|  | Prospare (N=77) | | Standard of Care (N=81) | | Total | |
|  | N | % | N | % | N | % |
| Haematuria |  |  |  |  |  |  |
| Grade 0 | 69 | 89.6 | 75 | 92.6 | 144 | 91.1 |
| Grade 1 | 1 | 1.3 | 2 | 2.5 | 3 | 1.9 |
| Not evaluated | 7 | 9.1 | 4 | 4.9 | 11 | 7.0 |
|  |  |  |  |  |  |  |
| Urinary frequency |  |  |  |  |  |  |
| Grade 0 | 49 | 63.6 | 53 | 65.4 | 102 | 64.6 |
| Grade 1 | 19 | 24.7 | 23 | 28.4 | 42 | 26.6 |
| Grade 2 | 2 | 2.6 | 1 | 1.2 | 3 | 1.9 |
| Not evaluated | 7 | 9.1 | 4 | 4.9 | 11 | 7.0 |
|  |  |  |  |  |  |  |
| Urinary incontinence |  |  |  |  |  |  |
| Grade 0 | 53 | 68.8 | 59 | 72.8 | 112 | 70.9 |
| Grade 1 | 15 | 19.5 | 17 | 21.0 | 32 | 20.3 |
| Grade 2 | 1 | 1.3 | 1 | 1.2 | 2 | 1.3 |
| Grade 3 | 1 | 1.3 | 0 | 0.0 | 1 | 0.6 |
| Not evaluated | 7 | 9.1 | 4 | 4.9 | 11 | 7.0 |
|  |  |  |  |  |  |  |
| Urinary retention |  |  |  |  |  |  |
| Grade 0 | 67 | 87.0 | 76 | 93.8 | 143 | 90.5 |
| Grade 1 | 3 | 3.9 | 0 | 0.0 | 3 | 1.9 |
| Grade 2 | 0 | 0.0 | 1 | 1.2 | 1 | 0.6 |
| Not evaluated | 7 | 9.1 | 4 | 4.9 | 11 | 7.0 |
|  |  |  |  |  |  |  |
| Urinary urgency |  |  |  |  |  |  |
| Grade 0 | 52 | 67.5 | 59 | 72.8 | 111 | 70.3 |
| Grade 1 | 18 | 23.4 | 18 | 22.2 | 36 | 22.8 |
| Not evaluated | 7 | 9.1 | 4 | 4.9 | 11 | 7.0 |
|  |  |  |  |  |  |  |
| Worst CTCAE Bladder score |  |  |  |  |  |  |
| Grade 0 | 34 | 44.2 | 36 | 44.4 | 70 | 44.3 |
| Grade 1 | 32 | 41.6 | 38 | 46.9 | 70 | 44.3 |
| Grade 2 | 3 | 3.9 | 3 | 3.7 | 6 | 3.8 |
| Grade 3 | 1 | 1.3 | 0 | 0.0 | 1 | 0.6 |
| Not evaluated | 7 | 9.1 | 4 | 4.9 | 11 | 7.0 |

## Table 7 – CTCAE Gastrointestinal (GI) toxicity at 18 weeks

|  | Randomisation group (patients who received radiotherapy only) | | | | | |
| --- | --- | --- | --- | --- | --- | --- |
|  | Prospare (N=77) | | Standard of Care (N=81) | | Total | |
|  | N | % | N | % | N | % |
| Abdominal pain |  |  |  |  |  |  |
| Grade 0 | 66 | 85.7 | 73 | 90.1 | 139 | 88.0 |
| Grade 1 | 4 | 5.2 | 4 | 4.9 | 8 | 5.1 |
| Not evaluated | 7 | 9.1 | 4 | 4.9 | 11 | 7.0 |
|  |  |  |  |  |  |  |
| Constipation |  |  |  |  |  |  |
| Grade 0 | 68 | 88.3 | 72 | 88.9 | 140 | 88.6 |
| Grade 1 | 2 | 2.6 | 3 | 3.7 | 5 | 3.2 |
| Grade 2 | 0 | 0.0 | 1 | 1.2 | 1 | 0.6 |
| Not evaluated | 7 | 9.1 | 5 | 6.2 | 12 | 7.6 |
|  |  |  |  |  |  |  |
| Diarrhoea |  |  |  |  |  |  |
| Grade 0 | 60 | 77.9 | 66 | 81.5 | 126 | 79.7 |
| Grade 1 | 7 | 9.1 | 11 | 13.6 | 18 | 11.4 |
| Grade 2 | 3 | 3.9 | 0 | 0.0 | 3 | 1.9 |
| Not evaluated | 7 | 9.1 | 4 | 4.9 | 11 | 7.0 |
|  |  |  |  |  |  |  |
| Proctitis |  |  |  |  |  |  |
| Grade 0 | 63 | 81.8 | 67 | 82.7 | 130 | 82.3 |
| Grade 1 | 6 | 7.8 | 8 | 9.9 | 14 | 8.9 |
| Grade 2 | 1 | 1.3 | 2 | 2.5 | 3 | 1.9 |
| Not evaluated | 7 | 9.1 | 4 | 4.9 | 11 | 7.0 |
|  |  |  |  |  |  |  |
| Rectal haemorrhage |  |  |  |  |  |  |
| Grade 0 | 67 | 87.0 | 76 | 93.8 | 143 | 90.5 |
| Grade 1 | 3 | 3.9 | 1 | 1.2 | 4 | 2.5 |
| Not evaluated | 7 | 9.1 | 4 | 4.9 | 11 | 7.0 |
|  |  |  |  |  |  |  |
| Rectal pain |  |  |  |  |  |  |
| Grade 0 | 68 | 88.3 | 71 | 87.7 | 139 | 88.0 |
| Grade 1 | 1 | 1.3 | 5 | 6.2 | 6 | 3.8 |
| Grade 2 | 1 | 1.3 | 1 | 1.2 | 2 | 1.3 |
| Not evaluated | 7 | 9.1 | 4 | 4.9 | 11 | 7.0 |
|  |  |  |  |  |  |  |
| Worst CTCAE GI score |  |  |  |  |  |  |
| Grade 0 | 49 | 63.6 | 52 | 64.2 | 101 | 63.9 |
| Grade 1 | 17 | 22.1 | 22 | 27.2 | 39 | 24.7 |
| Grade 2 | 4 | 5.2 | 3 | 3.7 | 7 | 4.4 |
| Not evaluated | 7 | 9.1 | 4 | 4.9 | 11 | 7.0 |

## Table 8 – CTCAE sexual dysfunction at 18 weeks

|  | Randomisation group (patients who received radiotherapy only) | | | | | |
| --- | --- | --- | --- | --- | --- | --- |
|  | Prospare (N=77) | | Standard of Care (N=81) | | Total | |
|  | N | % | N | % | N | % |
| Erectile dysfunction |  |  |  |  |  |  |
| Grade 0 | 2 | 2.6 | 9 | 11.1 | 11 | 7.0 |
| Grade 1 | 13 | 16.9 | 16 | 19.8 | 29 | 18.4 |
| Grade 2 | 25 | 32.5 | 37 | 45.7 | 62 | 39.2 |
| Grade 3 | 16 | 20.8 | 9 | 11.1 | 25 | 15.8 |
| Not evaluated | 21 | 27.3 | 10 | 12.3 | 31 | 19.6 |

## Table 9 – EPIC bowel score at pre-radiotherapy and 18 weeks

|  | Randomisation group | | |
| --- | --- | --- | --- |
|  | Prospare (N=77) | Standard of Care (N=81) | Total |
| EPIC score at 18 weeks post RT |  |  |  |
| Mean (sd) | 92.4 (11.9) | 91.3 (11.8) | 91.8 (11.8) |
| Median (IQR) | 96 (91, 100) | 95.5 (89, 100) | 96 (89,100) |
| N nonmissing values | 56 | 60 | 116 |
| EPIC score pre-RT |  |  |  |
| Mean (sd) | 96.7 (4.5) | 96.2 (5.2) | 96.4 (4.9) |
| Median (IQR) | 98 (96, 100) | 98 (96, 100) | 98 (96, 100) |
| N nonmissing values | 65 | 70 | 135 |

## Table 10 – IPSS symptom scores pre-radiotherapy and at 18 weeks

*The International Prostate Symptom Score (IPSS) is summarised below. The overall score is calculated as the sum of 7 questions regarding individual symptoms, and is on a scale from 0 to 35, with a higher score indicating more severe symptoms. The overall score is summarised, and counts shown for patients categorised by overall score as mild (0-7), moderate (8-19), or severe (20-35). Answers to an additional 8th question regarding overall symptom-related quality of life, are also tabulated.*

|  |  | Randomisation group | | | | | |
| --- | --- | --- | --- | --- | --- | --- | --- |
|  |  | Prospare (N=77) | | Standard of Care (N=81) | | Total | |
|  | Symptom score at 18 wks post RT | | |  | |  | |
|  | Mean (sd) | 5.4 (5.0) | | 5.8 (4.5) | | 5.6 (4.7) | |
|  | Median (IQR) | 4 (2, 8) | | 4 (3, 8) | | 4 (3, 8) | |
|  | N nonmissing values | 55 | | 59 | | 114 | |
|  |  | N | % | N | % | N | % |
|  | Mild (0-7) | 41 | 74.5 | 43 | 72.9 | 84 | 73.7 |
|  | Moderate (8-19) | 13 | 23.6 | 14 | 23.7 | 27 | 23.7 |
|  | Severe (20-35) | 1 | 1.8 | 2 | 3.4 | 3 | 2.6 |
|  | Symptom score pre RT |  | |  | |  | |
|  | Mean (sd) | 4.7 (3.5) | | 5.0 (4.0) | | 4.8 (3.8) | |
|  | Median (IQR) | (2,7) | | (2,7) | | (2,7) | |
|  | N nonmissing values | 65 | | 68 | | 133 | |
|  | | N | % | N | % | N | % |
| Mild (0-7) | | 54 | 83.1 | 53 | 77.9 | 107 | 80.5 |
| Moderate (8-19) | | 11 | 16.9 | 15 | 22.1 | 26 | 19.5 |
| Severe (20-35) | | 0 | 0 | 0 | 0 | 0 | 0 |

## Table 11 – IPSS QoL scores pre-radiotherapy and at 18 weeks

| If you were to spend the rest of your life with your urinary condition just the way it is now, how would you feel about that? | Randomisation group | | |
| --- | --- | --- | --- |
|  | Prospare (N=77) | Standard of Care (N=81) | Total |

| 18 wks | N | % | N | % | N | % |
| --- | --- | --- | --- | --- | --- | --- |
| Delighted | 11 | 19.3 | 13 | 21.7 | 24 | 20.5 |
| Pleased | 13 | 22.8 | 14 | 23.3 | 27 | 23.1 |
| Mostly satisfied | 17 | 29.8 | 14 | 23.3 | 31 | 26.5 |
| Mixed | 10 | 17.5 | 9 | 15.0 | 19 | 16.2 |
| Mostly dissatisfied | 2 | 3.5 | 7 | 11.7 | 9 | 7.7 |
| Unhappy | 4 | 7.0 | 3 | 5.0 | 7 | 6.0 |
| Pre RT |  |  |  |  |  |  |
| Delighted | 17 | 25.4 | 14 | 20.0 | 31 | 22.6 |
| Pleased | 17 | 25.4 | 17 | 24.3 | 34 | 24.8 |
| Mostly satisfied | 17 | 25.4 | 19 | 27.1 | 36 | 26.3 |
| Mixed | 10 | 14.9 | 12 | 17.1 | 22 | 16.1 |
| Mostly dissatisfied | 2 | 3.0 | 3 | 4.3 | 5 | 3.6 |
| Unhappy | 4 | 6.0 | 5 | 7.1 | 9 | 6.6 |

## Table 12 – IIEF-5 score (continuous) pre-radiotherapy and at 18 weeks

*The International Index of Erectile Function (IIEF-5) score at 18 weeks post radiotherapy is summarised below. Scores are on a scale from 5 to 25, with a higher score indicating better function. The overall score is summarised, and counts shown for patients categorised by overall score.*

|  | Randomisation group (patients who received radiotherapy only) | | |
| --- | --- | --- | --- |
|  | Prospare (N=77) | Standard of Care (N=81) | Total |
| IIEF-5 score at 18 wks |  |  |  |
| Mean (sd) | 7.4 (5.2) | 8.0 (5.6) | 7.7 (5.4) |
| Median (IQR) | 5 (5,5) | 5 (5,10) | 5 (5,6) |
| N nonmissing values | 49 | 51 | 100 |
| IIEF-5 score pre-RT |  |  |  |
| Mean (sd) | 7.7 (5.7) | 9.9 (6.3) | 8.8 (6.1) |
| Median (IQR) | 5 (5,6) | 5 (5,14) | 5 (5,12) |
| N nonmissing values | 57 | 61 | 118 |

## Table 13 – IIEF-5 score (categorical) pre-radiotherapy and at 18 weeks

|  | Randomisation group (patients who received radiotherapy only) | | |
| --- | --- | --- | --- |
|  | Prospare (N=77) | Standard of Care (N=81) | Total |

| IIEF-5 at 18 wks | N | % | N | % | N | % |
| --- | --- | --- | --- | --- | --- | --- |
| Severe (5-7) | 39 | 79.6 | 38 | 74.5 | 77 | 77.0 |
| Moderate (8-11) | 2 | 4.1 | 2 | 3.9 | 4 | 4.0 |
| Mild to moderate (12-16) | 3 | 6.1 | 6 | 11.8 | 9 | 9.0 |
| Mild (17-21) | 3 | 6.1 | 1 | 2.0 | 4 | 4.0 |
| No dysfunction (22-25) | 2 | 4.1 | 4 | 7.8 | 6 | 6.0 |
| IIEF-5 pre-RT |  |  |  |  |  |  |
| Severe (5-7) | 46 | 80.7 | 35 | 57.4 | 81 | 68.6 |
| Moderate (8-11) | 1 | 1.8 | 5 | 8.2 | 6 | 5.1 |
| Mild to moderate (12-16) | 2 | 3.5 | 8 | 13.1 | 10 | 8.5 |
| Mild (17-21) | 4 | 7.0 | 9 | 14.8 | 13 | 11.0 |
| No dysfunction (22-25) | 4 | 7.0 | 4 | 6.6 | 8 | 6.8 |

## Table 14 – IBDQ score pre-radiotherapy and at 18 weeks

*The Irritable Bowel Disease Questionnaire (IBDQ) score at 18 weeks post radiotherapy is summarised below. Scores are on a scale from 0 to 224, with a higher score indicating better health related quality of life.*

|  | Randomisation group | | |
| --- | --- | --- | --- |
|  | Prospare (N=77) | Standard of Care (N=81) | Total |
| IBDQ score at 18 wks |  |  |  |
| Mean (sd) | 200.3 (18.8) | 202.6 (15.5) | 201.5 (17.1) |
| Median (IQR) | 205.5 (193, 213) | 206 (191, 214.5) | 205.5 (192.5, 214) |
| N nonmissing values | 56 | 60 | 116 |
| IBDQ score pre RT |  |  |  |
| Mean (sd) | 206.1 (14.8) | 206.4 (10.3) | 206.3 (12.6) |
| Median (IQR) | 210.5 (198, 216) | 207.5 (210, 213.8) | 209 (199, 215) |
| Number of nonmissing values | 66 | 69 | 135 |

## Table 15 – Vaizey score pre-radiotherapy and at 18 weeks

*The Vaizey faecal incontinence questionnaire is scored on a scale of 0 to 24, with high scores representing worse symptoms.*

|  | Randomisation group | | |
| --- | --- | --- | --- |
|  | Prospare (N=77) | Standard of Care (N=81) | Total |
| Vaizey score at 18 wks |  |  |  |
| Mean (sd) | 2.5 (2.8) | 2.9 (3.9) | 2.7 (3.4) |
| Median (IQR) | 1 (0, 4) | 1 (0,4) | 1 (0,4) |
| N nonmissing values | 56 | 59 | 115 |
| Vaizey score pre RT |  |  |  |
| Mean (sd) | 2.2 (2.4) | 1.4 (2.0) | 1.8 (2.3) |
| Median (IQR) | 2 (0,4) | 0 (0,2) | 1 (0,4) |
| N nonmissing values | 65 | 69 | 134 |

## Table 16 – SF-12 scores pre-radiotherapy and at 18 weeks

*The SF-12 questionnaire scores health related quality of life with summary scores over two domains, physical functioning and mental well-being. Scores were standardized against the US general population circa 1990, using the Stata module sf12, so that a score of 50 matches the expected population mean, scores above 50 represent better than average quality of life, and scores below 50 represent below-average health.*

|  | Randomisation group | | |
| --- | --- | --- | --- |
|  | Prospare (N=77) | Standard of Care (N=81) | Total |
| Physical functioning at 18wks |  |  |  |
| Mean (sd) | 51.8 (8.4) | 51.6 (6.9) | 51.7 (7.6) |
| Median (IQR) | 54.8 (49.0, 57.5) | 53.8 (47.4, 56.4) | 54.4 (47.6, 56.7) |
| N nonmissing values | 58 | 58 | 116 |
| Mental well-being at 18wks |  |  |  |
| Mean (sd) | 51.5 (7.9) | 54.5 (6.9) | 53.0 (7.5) |
| Median (IQR) | 53.5 (46.6, 57.7) | 55.1 (50.8, 60.1) | 54.2 (49.5, 58.2) |
| N nonmissing values | 58 | 58 | 116 |
| Physical functioning pre RT |  |  |  |
| Mean (sd) | 53.6 (7.9) | 53.3 (6.6) | 53.4 (7.2) |
| Median (IQR) | 56.2 (51.4, 58.3) | 55.1 (51.7, 57.2) | 55.9 (51.7, 57.8) |
| Number of nonmissing values | 64 | 68 | 132 |
| Mental well-being pre RT |  |  |  |
| Mean (sd) | 52.1 (8.4) | 52.5 (8.0) | 52.3 (8.2) |
| Median (IQR) | 53.5 (49.1, 57.2) | 54.6 (48.8, 57.5) | 54.2 (49.1, 57.2) |
| Number of nonmissing values | 64 | 68 | 132 |

## Table 17 – Patient reported ProSpare tolerability before treatment

*For patients randomised to ProSpare only , the following table summarise patient-reported tolerability before radiotherapy treatment.*

|  | Prospare (N=77) | |  |
| --- | --- | --- | --- |
|  | N | % (of all patients) | % (of responders) |
| Instructions easy to understand |  |  |  |
| Very easy | 43 | 55.8 | 67.2 |
| Quite easy | 17 | 22.1 | 26.6 |
| A little difficult | 3 | 3.9 | 4.7 |
| Very difficult | 1 | 1.3 | 1.6 |
| No response | 13 | 16.9 |  |
|  |  |  |  |
| Easy to insert first time |  |  |  |
| Very easy | 13 | 16.9 | 20.0 |
| Quite easy | 28 | 36.4 | 43.1 |
| A little difficult | 20 | 26.0 | 30.8 |
| Very difficult | 4 | 5.2 | 6.2 |
| No response | 12 | 15.6 |  |
|  |  |  |  |
| Painful/uncomfortable first time |  |  |  |
| Not at all | 31 | 40.3 | 47.7 |
| A little | 29 | 37.7 | 44.6 |
| Quite a bit | 3 | 3.9 | 4.6 |
| Very much | 2 | 2.6 | 3.1 |
| No response | 12 | 15.6 |  |
|  |  |  |  |
| Easy to insert after practise |  |  |  |
| Very easy | 26 | 33.8 | 40.6 |
| Quite easy | 27 | 35.1 | 42.2 |
| A little difficult | 10 | 13.0 | 15.6 |
| Very difficult | 1 | 1.3 | 1.6 |
| No response | 13 | 16.9 |  |
|  |  |  |  |
| Painful/uncomfortable after practise |  |  |  |
| Not at all | 41 | 53.2 | 64.1 |
| A little | 22 | 28.6 | 34.4 |
| Quite a bit | 1 | 1.3 | 1.6 |
| No response | 13 | 16.9 |  |

## Table 18 – Patient reported ProSpare tolerability after treatment

*For patients randomised to ProSpare only , the following table summarise patient-reported tolerability before radiotherapy treatment.*

|  | Prospare (N=77) | |  |
| --- | --- | --- | --- |
|  | N | % (of all patients) | % (of responders) |
| Would recommend if resulted in  fewer side effects from treatment |  |  |  |
| Yes | 49 | 63.6 | 96.1 |
| No | 1 | 1.3 | 2.0 |
| Don't know | 1 | 1.3 | 2.0 |
| No response | 26 | 33.8 |  |
|  |  |  |  |
| Would recommend if treatment more accurate  (same side effects) | | |  |
| Yes | 50 | 64.9 | 98.0 |
| No | 1 | 1.3 | 2.0 |
| No response | 26 | 33.8 |  |
|  |  |  |  |
| Tolerable during treatment |  |  |  |
| Yes | 50 | 64.9 | 98.0 |
| No | 1 | 1.3 | 2.0 |
| No response | 26 | 33.8 |  |
|  |  |  |  |
| Easy to insert during treatment |  |  |  |
| Very easy | 16 | 20.8 | 31.4 |
| Quite easy | 30 | 39.0 | 58.8 |
| A little difficult | 4 | 5.2 | 7.8 |
| Very difficult | 1 | 1.3 | 2.0 |
| No response | 26 | 33.8 |  |
|  |  |  |  |
| Painful/uncomfortable during treatment |  |  |  |
| Not at all | 27 | 35.1 | 54.0 |
| A little | 22 | 28.6 | 44.0 |
| Quite a bit | 1 | 1.3 | 2.0 |
| No response | 27 | 35.1 |  |

## Table 19 – Rectal dose characteristics with and without ProSPare® use

|  | ProSpare® | No ProSpare® |
| --- | --- | --- |
| % Prescribed Dose | Median (IQR) calculated volume (%) | Median (IQR) calculated volume (%) |
| 45 | 74.4 (62.8-77.8) | 76.5 (70.7-79.7) |
| 61 | 54.3 (43.4-61) | 60.6 (52.7-68.9) |
| 76 | 38.9 (31.2-45) | 44.5 (37.8-53.6) |
| 91 | 22.8 (18.4-28.2) | 29.9 (24.9-34.3) |
| 98 | 8.7 (6.4-11.1) | 15.7 (11.3-19.4) |

## Table 20 – Anal Canal dose characteristics with and without ProSPare® use

|  | ProSpare® | No ProSpare® |
| --- | --- | --- |
| % Prescribed Dose | Median (IQR) calculated volume (%) | Median (IQR) calculated volume (%) |
| 51 | 20.6 (12.4-31.8) | 33.7 (18.6-45.5) |
| 61 | 15.3 (9.8-24.4) | 26.5 (12-38.1) |
| 91 | 4.1 (1.7-7.6) | 9 (1.7-15.8) |
