# Supplementary material for: Preliminary evaluation of ProSpare®, a rectal obturator for prostate bed radiotherapy within the randomised multicentre POPS trial (NCT02978014)
Source: Front Oncol. 2026 Oct 12;16:1758462. doi: 10.3389/fonc.2026.1758462 (PMC13696223; doi:10.3389/fonc.2026.1758462)
Supplement: Supplementary file 3 [file DataSheet3.doc]

**POPS**

**A randomised phase II trial assessing Post-Operative use of ProSpare™, a rectal obturator in prostate cancer radiotherapy**

Statistical Analysis Plan

Version 2.0, 21.07.2023

| **Protocol Title and CCR number:** | A randomised phase II trial assessing Post-Operative use of ProSpare™, a rectal obturator in prostate cancer radiotherapy |
| --- | --- |
| **Protocol Version and Date:** | Version: 9.0 Date: 28/12/2022 |
| **REC/MHRA Approval Date:** |  |
| **Chief Investigator (or Delegate):** | Dr Julia Murray |
| **Sponsor:** | The Institute of Cancer Research, 15 Cotswold Road, Sutton, Surrey, SM2 5NG |
| **Document Version:** | V2.0 |
| **Trial Statistician:** | Karen Thomas |

Contents

1	Trial Summary	7

2	Study objectives	7

2.1	Research hypothesis	7

2.2	Study objectives	7

2.2.1	Primary Objective:	7

2.2.2	Secondary Objectives:	7

2.2.3	Exploratory Objective:	7

3	Study endpoints	7

3.1	Primary endpoint:	7

3.2	Secondary endpoints:	8

3.3	Exploratory endpoint:	8

4	Study Methods	8

4.1	Study design	8

4.2	Randomisation and Stratification	8

4.3	Sample Size	8

4.4	Framework	9

4.5	Statistical Interim Analysis and stopping guidance	9

4.6	Timing of Analysis	10

4.7	Timing of outcome assessment	10

5	Statistical principles	10

5.1	Confidence intervals and p values	10

5.2	Adherence and protocol deviations	10

5.3	Analysis population	10

6	Trial Population	11

6.1	Screening Data	11

6.2	Recruitment	11

6.3	Withdrawal/ Follow up – level of withdrawal	11

6.4	Baseline patient characteristics	12

7	Analysis	12

7.1	Outcome definitions	12

7.2	Analysis Methods	14

7.3	Statistical Packages	17

**List o**f Abbreviations

| Abbreviation or special term | Explanation |
| --- | --- |
| CTCAE | Common Terminology Criteria for Adverse Events |
| IDMC | Independent Data Monitoring Committee |
| QOL | quality of life |
| SAP | Statistical Analysis Plan |
| IMRT | << >> |

**Amendment** history

| **Date** | **Version** | **Timing in relation to analysis** | **Brief description of change** | **Justification for change** |
| --- | --- | --- | --- | --- |
| 26.10.2021 | 1.0 | First draft prior to interim analysis | N/A | N/A |
| **21.07.2023** | **2.0** | **Following end of recruitment, prior to end of follow-up** | **Added details and clarification for primary analysis** |  |

# 1 Trial Summary

Patients planned to undergo post-prostatectomy intensity modulated radiotherapy (IMRT) are randomised between IMRT with vs without a rectal obdurator (ProSpare), in order to reduce long term side effects from radiotherapy.

# 2 Study objectives

## 2.1 Research hypothesis

The objective of this trial is to determine if a new radiotherapy device improves patient reported outcomes, particularly in relation to bowel symptoms. We propose that ProSpare™ online daily image-guided IMRT will improve bowel function compared with standard IMRT.

## 2.2 Study objectives

### 2.2.1 Primary Objective:

To compare the EPIC bowel scores for patients receiving adjuvant or salvage prostate bed radiotherapy treated with ProSpare™ image-guided IMRT versus the standard arm of IMRT with centre standard image-guidance at 2 years following radiotherapy.

### 2.2.2 Secondary Objectives:

- To compare acute and late bowel and bladder toxicity and sexual function between the two study arms
- To compare quality of life in patients randomised to the two study arms
- To evaluate biochemical progression free survival

### 2.2.3 Exploratory Objective:

To analyse the dosimetric consequences of the two radiotherapy treatment techniques and relate these to toxicity endpoints

# 3 Study endpoints

## 3.1 Primary endpoint:

Patient reported outcome EPIC bowel function and bother using the EPIC-bowel questionnaire at 2 years following prostate bed radiotherapy.

## 3.2 Secondary endpoints:

- Acute bowel and bladder toxicity at week 18 from start of radiotherapy (CTCAEv4, RTOG, Gulliford Rectal scoring, EPIC, IPSS)
- Late (1 and 2 year) bowel and bladder toxicity (EPIC, RTOG, modified IBDQ, IPSS)
- Sexual bother and function and change in patient reported erectile function (EPIC, IIEF-5)
- Faecal incontinence (Vaizey incontinence questionnaire)
- Biochemical (PSA) progression free survival

## 3.3 Exploratory endpoint:

Dosimetric assessment of prostate bed and rectal dose with and without ProSpare^TM^ to correlate dose distribution with toxicity

# 4  Study Methods

## 4.1  Study design

This is a phase II multi-centre, open-label, randomised trial designed to demonstrate a difference in patient reported bowel function and bother using the EPIC questionnaire between patients treated with ProSpare™ IG-IMRT with reduced margins and those treated with IMRT, centre standard image-guidance and standard margins.

## 4.2 Randomisation and Stratification

Central randomisation will be performed by the Institute of Cancer Research Clinical Trials and Statistics Unit (ICR-CTSU). The randomisation process will use minimisation with a random element and with stratification factors listed below as balancing factors.

Patients will be stratified by:

- Clinical indication for prostate bed radiotherapy: adjuvant or salvage
- Centre
- Hormone therapy:
- No androgen therapy
- 6 months hormone therapy
- >6 months but <24 months
- ≥24months
- Boost given to macroscopic disease: yes or no.
- Irradiation of pelvic lymph nodes: yes or no.

## 4.3 Sample Size

The sample size is based on the primary endpoint which is the difference in the mean change between groups in patient reported EPIC bowel function and bother from baseline to 2 years after prostate bed radiotherapy.

There is no published literature detailing EPIC scores in the prostate bed radiotherapy setting. As the planned target volume is similar to prostate radiotherapy we have extrapolated the data from patient reported outcomes in patients who have received radical prostate radiotherapy using IMRT.

The primary endpoint of their study was the mean change in quality of life scores from pre-treatment to post treatment in the acute (<90 days), late (12-24 months) time periods. The clinically meaningful change in health related quality of life is defined as a mean change score exceeding half the standard deviation of the baseline value. In this study, which included 153 patients, the mean EPIC bowel score at baseline was 94.3, with a standard deviation of 10.9.

We therefore plan to use 5.45 as our minimum absolute difference between the two groups at two years to identify a clinically meaningful difference. 172 patients (86 patients per group) are required to detect a 5.45 point difference in the EPIC bowel score, with 90% power, 2-sided 5% alpha. To allow for a non-evaluable rate at 24 months of 30% due non-compliance with the 24 month questionnaire, it is assumed that 70% of patients will be evaluable for the primary endpoint. The target sample size is therefore 245 patients. An IDMC will assess EPIC questionnaire compliance rates at regular intervals and make recommendations for changes in sample size to ensure 172 evaluable patients as appropriate.

## 4.4 Framework

The primary endpointwill be compared between arms for superiority.

## 4.5 Statistical Interim Analysis and stopping guidance

The IDMC will review accrual, safety and efficacy data. The replacement of patients who withdraw from the study or who are not evaluable for reasons deemed not to be treatment related will be based on the advice of this committee.

As the proposed duration of recruitment is 24 months, it is unlikely that sufficient data on the efficacy i.e. biochemical progression free survival (PFS) to inform the early stopping rule of the trial for efficacy. Therefore, no formal stopping rule based on the biochemical PFS is proposed.

- Published data has shown no significant change in biochemical PFS in patients treated with image-guided radiotherapy and reduced margins in prostate radiotherapy; therefore, we do not feel that there should be an increase in this trial population.

There will be close monitoring of acute toxicity (defined at the time of week 18 post-radiotherapy) and tolerability of the ProSpare™ device throughout the total duration of radiotherapy in patients randomised to that arm. Early safety stopping rules will be finalised in liaison with the IDMC and will be based on the following assumptions:

- Acute toxicity: in a study of 176 patients treated with IMRT after radical prostatectomy in the salvage setting, the overall rate of acute grade ≥2 (CTCAE v.3) GI toxicity was 9.8% ^[27]^_._ Therefore the proportion of patients, who have no acute grade 2+ GI toxicity, as assessed on CTCAE v.4, should be ≥90%. If this proportion falls to ≤75% - equivalent to 8 or more of the first 50 patients exhibiting GI toxicity ≥G2 at 18 weeks - the IDMC would be asked to advise on whether the trial should be stopped on safety grounds. Given these criteria, there is an 88% chance the trial will not be stopped if the grade 2+ GI toxicity free rate is 90% but only a 5% chance it will not be stopped if the true GI toxicity free rate is 70%.
- Tolerability of ProSpare™ device: if <85% of patients tolerate the device for <85% of all radiotherapy treatments, assessed using the ProSpare^TM^ safety assessment questionnaire at 10 weeks (-1/+ 2 weeks) from start of radiotherapy. This has been calculated to take into account the total number of fractions missed due to poor tolerability, which would extend treatment by more than one week.

## 4.6 Timing of Analysis

Analysis of trial data will be done in two phases. Each endpoint will be analysed and reported only once (with the exception of biochemical PFS). The first analysis will occur after all patients have completed their 18 week post-radiotherapy assessment, and will include all acute toxicity endpoints, tolerability, patient reported outcomes to 18 weeks only, and dosimetric evaluation. The second analysis will occur after all patients have completed their 24 month post-radiotherapy assessment, and will include all late toxicity and patients reported outcomes (including sexual function), and biochemical PFS.

## 4.7 Timing of outcome assessment

The schedule of study procedures is given in the protocol along with the expected visit dates and visit windows. Patients are followed up for a maximum of five years and a minimum of two years after radiotherapy, with follow-up for all patients finished once the last patient to be treated has reached two years post radiotherapy.

# 5 Statistical principles

## 5.1 Confidence intervals and p values

All statistical tests will be two-sided using a 0.05 significance level. Confidence intervals will be 95% and two-sided.

## 5.2 Adherence and protocol deviations

Counts and percentages of patients who completed all radiotherapy as planned (with respect to number of fractions, total dose and boost dose given) will be presented by arm. For patients who did not complete all radiotherapy as planned, counts and percentages of patients by the total number of missed fractions will be presented by arm. Cross-tabulations showing number of patients with planned vs actual boost doses given will be shown by arm.

## 5.3 Analysis population

The ProSpare devise is hypothesized to reduce long-term toxicity by reducing the radiation dose to normal tissue. Therefore, although the analysis will be on an intention to treat basis insofar as patients randomised to the ProSpare arm will be analysed as belonging to that arm whether or not they did use the experimental device during their treatment, patients who did not receive any dose of radiotherapy will be excluded from the analysis (the number and percentage of such patients will be reported by arm). The following analysis population are defined:

Primary analysis set: all randomised patients who received at least one dose of radiotherapy

# 6 Trial Population

## 6.1 Screening Data

Screening logs have not been collected for this trial.

## 6.2 Recruitment

A modified CONSORT diagram will be presented showing the number of patients at each of the following stages, by arm:

- Randomised
- Withdrew prior to radiotherapy
- Commencing radiotherapy
- Withdrew after commencing radiotherapy but before 2 years
- Remained on trial at 2 years post-radiotherapy (eligible to complete primary endpoint data)
- Completed primary endpoint data
- Withdrew after 2 years post radiotherapy but before end of follow-up at 5 years post radiotherapy*
- Remained in the trial until 5years post radiotherapy*

*this information will be presented at the final analysis only

## 6.3 Withdrawal/ Follow up – level of withdrawal

The numbers of patients and reasons for withdrawal will be reported by arm, grouped according to timepoint (pre-radiotherapy, before 2 years post radiotherapy, after 2 years post radiotherapy).

## 6.4 Baseline patient characteristics

Baseline characteristics of patients in the primary analysis population will be presented by arm, including all stratification variables. No statistical tests will be done to compare baseline characteristics between arms. Continuous variables will be summarised using median and interquartile ranges (and/or means and standard deviations depending on distribution), categorical variables will be reported using counts and percentages. The following baseline characteristics will be presented:

Patient characteristics

Age at randomisation

History of: IDB or diverticular disease; symptomatic haemorrhoids in previous 12 months; treated hypertension; anticoagulant therapy; statins (yes/no to each)

WHO PS (categorical)

Previous pelvic surgery

Disease characteristics

Gleason score (total score, as a categorical variable)

PSA pre-prostatectomy, pre-hormone therapy (or pre-radiotherapy if not planned for hormones)

Testosterone (pre-hormone therapy, or pre radiotherapy if not planned for hormones)

Extraprostatic extension

Positive margins at prostatectomy

Seminal vesicle involvement

Lymph node involvement

Treatment

Previous chemotherapy given (yes/no)

Types of hormone therapy (LHRH agonists/antagonists with short course anti-androgens; maximal androgen blockade; other)

Planned duration of hormone therapy (<=6 months; between 6 and 24 months; >=24 months)

Type of prostate bed radiotherapy (adjuvant or salvage)

Planned RT boost (yes/no)

Planed RT to pelvic lymph nodes (yes/no)

# 7 Analysis

## 7.1 Outcome definitions

Primary outcome

The primary outcome of the study is the EPIC bowel score at two years post radiotherapy. Full instructions for scoring the EPIC questionnaire, including the details for calculating the bowel domain summary score (the primary endpoint of this study), are available in a separate document to this SAP which has been downloaded from <https://medicine.umich.edu/dept/urology/research/epic>.

Secondary outcomes

Other EPIC summary scores will be calculated using standard published methodology as referenced above :

EPIC urinary summary score

EPIC sexual summary score

EPIC hormonal summary score

The IPSS questionnaire contains 7 symptom related questions which are each answered on an ordinal scale ranging from 0 (no symptoms) to 5 (worst possible case). The answers are added together to calculate an overall symptom score ranging from 0 to 35. One additional stand-alone question collects the overall quality of life due to urinary symptoms on an ordinal scale from 0 (best case) to 6 (worst).

The IIEF-5 questionnaire contains five questions each answered on an ordinal scale from 1 (worst case) to 5 (best case). A single overall score is calculated using the sum of all answers, with resulting score ranging from a minimum of 5 (severe erectile dysfunction) to a maximum of 25 (no erectile dysfunction)

The IBDQ questionnaire, measuring irritable bowel disease specific quality of life, contains a total of 32 questions which are each answered on an ordinal scale ranging from 1 for the worst case answer to 7 for the best case. A single summary score is calculated as the sum of answers to each of the 32 questions. Possible scores range from a worst score of 32 to the best of 224.

The Vaizey incontinence questionnaire has a total of 7 questions, the first 4 relate to frequency of incontinence and are answered using an ordinal scale from 0 (never) to 4 (daily). Questions 5 and 6 relate to use of pads/medicines and are answered as a binary yes (scoring 2) or no (scoring 0). The final question relates to ability to defer defecation and is also answered using binary yes (scoring 0) or no (scoring 4). A single overall score is calculated as the sum of all 7 answers.

The SF-12 questionnaire is used to generate two scores, measuring physical function and mental well-being.

Radiotherapy toxicity is measured using RTOG scores (one score each for bladder and lower GI toxicity, with grades given from 0 for no toxicity to grade 5 for fatal toxicity), CTCAE scores which are collected both for specific targeted GI, renal & urinary toxicities, and erectile dysfunction, and for any other toxicity), and using the Gulliford rectal scoring system which categorises five defined rectal symptoms and any rectal symptoms overall as either not present, mild, moderate or severe. RTOG scores are collected from 2 weeks and up to 24 months after the start of radiotherapy, CTCAE and Gulliford scores commence at 18 weeks after the start of radiotherapy and continue to 24 months.

Biochemical progression free survival is measured from the date of randomization until the first date of biochemical progression, which is defined as a rise of at least 0.2 ng/ml above the post-radiotherapy nadir, if confirmed by a second consecutive values obtained 4 or more weeks (28 days) later. Patients without confirmed biochemical progression will be censored at the date of the last recorded PSA value.

Tolerability is assessed using one pre-treatment and one post treatment questionnaire. Each questionnaire contains five questions with answers given either on a binary or ordinal scale, no summary score is available – answers to each questions will be reported separately. In addition, any device related safety incidents (bleeding, pain, allergic reaction, other trauma or tissue damage) are logged as adverse events.

Exploratory dosimetric comparison between arms will be performed separately to the main analysis, using data not collected in the main trial database, and is not covered by this SAP.

## 7.2 Analysis Methods

Primary endpoint

Mean and standard deviation of EPIC Bowel score at two years will be presented by arm. A linear mixed-effects model will be fitted to predict EPIC bowel score at two years as a function of allocated treatment arm, baseline score, and the following pre-specified clinical factors which were used as balancing factors in the randomisation schema :

- Clinical indication for prostate bed radiotherapy: adjuvant or salvage
- Centre (as a random effect)
- Hormone therapy:
- No androgen therapy
- 6 months hormone therapy
- >6 months but <24 months
- ≥24months
- Boost given to macroscopic disease: yes or no.
- Irradiation of pelvic lymph nodes: yes or no.
- Biochemical recurrence with regional lymph node involvement: yes or no

A likelihood ratio test will be performed to compare the full model with a reduced model removing the effect of allocated treatment arm, with a significance threshold of 0.05. The primary effect estimate will be the model coefficient for treatment arm with a 95% confidence interval, which measures the estimated difference in score between arms after adjustment. As a sensitivity analysis, an unpaired t-test will also be done to compare unadjusted change from baseline to two years between arms.

Where available, questionnaires completed immediately before radiotherapy will be used as the baseline. Where this is missing but a questionnaire has been completed at screening, the screening values will be used as baseline data. Patients with missing scores at 24 months may have their 18 month score used instead for the primary analysis, where available. The number and percentage of patients who have missing data at either baseline or two years will be reported by arm, if this is unbalanced across the arms or the overall percentage of patients with missing data is more than the expected rate of 30%, then multiple imputation with chained equations will be used (5 datasets will be produced, with results combined using Rubin’s rules) for the primary endpoint, and a sensitivity analysis will also be performed using non-missing patients only. Patients who have missing data caused by death prior to two years will not have their data imputed.

Mean and standard deviation of EPIC bowel score will be presented graphically by arm, for all available timepoints.

Secondary endpoints

The following quality of life scores will be treated as continuous outcomes, and presented graphically as mean and standard deviation (or as median and interquartile range if their distribution appears markedly non-normal) at each timepoint, by arm: EPIC urinary, sexual, and hormonal summary scores; IPSS symptom score; IIEF score; IBDQ summary score; Vaizey score; SF-12 physical and mental scores.

The following categorical outcomes will be reported as counts and percentages by arm, at two years and graphically (using stacked bar charts) over all timepoints: IPSS overall quality of life score; worst bladder RTOG grade; worst bowel RTOG grade; worst GI CTCAE grade; worst renal/urinary CTCAE grade; CTCAE erectile dysfunction grade; Gulliford severity scores; patient reported tolerability. No formal statistical comparisons between arms will be done for these outcomes.

Biochemical PFS will be summarised graphically by arm using a Kaplan-Meier plot. Cox regression will be used to estimate the hazard ratio with 95% confidence interval (adjusted using the same clinical factors as listed above for the primary endpoint) between arms.

## 7.3 Statistical Packages

The analysis will be carried out using Stata.
